# Flow Hydrodediazoniation of Aromatic Heterocycles

**DOI:** 10.3390/molecules24101996

**Published:** 2019-05-24

**Authors:** Liesa Röder, Alexander J. Nicholls, Ian R. Baxendale

**Affiliations:** 1 Department of Biology, Chemistry, and Pharmacy, Freie Universität Berlin, 14195 Berlin, Germany; liesa.roder@durham.ac.uk; 2 Department of Chemistry, University of Durham, South Road, Durham DH1 3LE, UK; alexander.j.nicholls@durham.ac.uk

**Keywords:** flow chemistry, continuous processing, deamination, hydrodediazoniation, heterocylic, isopentyl nitrite

## Abstract

Continuous flow processing was applied for the rapid replacement of an aromatic amino group with a hydride. The approach was applied to a range of aromatic heterocycles, confirming the wide scope and substituent-tolerance of the processes. Flow equipment was utilized and the process optimised to overcome the problematically-unstable intermediates that have restricted yields in previous studies relying on batch procedures. Various common organic solvents were investigated as potential hydride sources. The approach has allowed key structures, such as amino-pyrazoles and aminopyridines, to be deaminated in good yield using a purely organic-soluble system.

## 1. Introduction

The use of diazotisation as a regioselective functionalisation strategy for aromatic compounds is inherent to the design of synthetic pathways for a variety of target products. A less utilised aspect is the replacement of an aromatic amine (via a diazonium) for a proton; essentially a defunctionalisation step, this can be a creative and useful sequence as the amine is a powerful directing group in more complex, multi-stage syntheses [1,2]. Historically, simple anilines that form relatively-stable diazonium salts are readily deaminated in good yields under batch conditions [3,4,5]. However, many aromatic heterocycles, such as 2-amino-pyrazoles and pyridines, do not form stable diazonium salts and hence batch procedures form the target deaminated product in generally poor yield. Recent studies have attempted to improve the efficiency for more challenging systems using various catalysts, such as salicylic acid or gallic acid, to both stabilize the diazonium compound (by trapping) and controlling the rate that the diazonium compound radicalizes [6,7]. However, more sophisticated heterocyclic structures, such as 2-aminopyrimidine and 2-aminobenzothiazole, could still be deaminated only in modest yields. Flow chemistry has been shown to be especially useful for the generation and handling of reactive/transient intermediates [8,9,10,11,12,13,14]. The primary benefits of performing such reactions in flow mostly arise from the improved mixing and superior heat dissipation [15,16]. Although elevated temperatures are often required for most of these heterocycles to undergo nitrosation, the overall process is very exothermic, and consequently the reacting molecules can form small, localized areas of very high temperatures, causing the sensitive diazonium intermediates to decompose. On a larger scale, this can also lead to run-away exothermic reactions. Advantageously using flow, the large surface area of the reaction vessel ensures that potential localized regions of undesired high temperatures and prorogation of heat-generation does not lead to substantial deviations of temperature from that of the reactor. Secondly, due to working with very unstable intermediates, it is imperative that there is effective mixing between the intermediates and the hydride source in order to reach the target product as soon as possible, which working in flow provides. The kinetics of at least some stages of this reaction are likely to be diffusion controlled, hence the enhanced mixing further improves the rate, allowing for an effective process omitting catalysts and radical initiators. The third benefit is in the application of temperatures substantially above the boiling point of the solvent, which has been found to be advantageous in this process. The solvents of choice were cyclic ethers, with tetrahydrofuran (THF) being the most effective.

## 2. Results and Discussion

**Figure 1:**
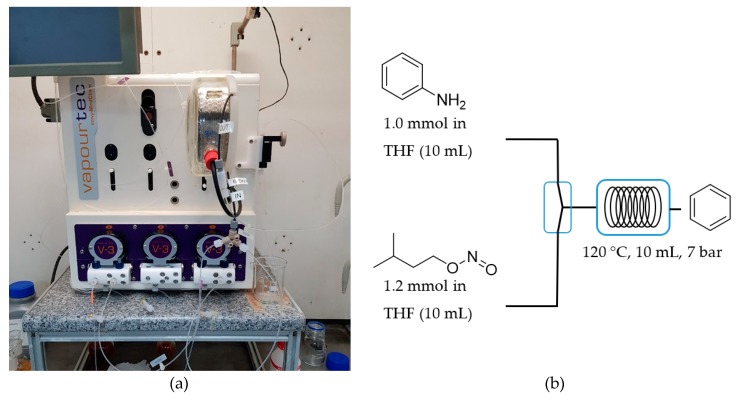
(**a**) The Vapourtech EasyMedChem system used to perform the experiments. (**b**) A diagram to show the general procedure of heterocyclic amine hydrodediazoniation.

Using the Vapourtech EasyMedChem system (Figure 1), we began investigating the principle conditions. Methyl 2-aminothiazole-5-carboxylate and methyl 5-amino-thiophene-2-carboxylate were selected as substrates, because thiazoles and thiophenes are structures that other investigations have struggled to deaminate in high yield [6,7]. The results presented were not obtained at steady state.

### 2.1. Solvent Selection

The criteria for the solvent (which is also the hydride source—see mechanism discussion) is that it should offer sufficient solubility for the starting material/diazonium intermediate and that it can undergo hydride abstraction (Table 1). Ethers were found to be the most effective, likely due to the oxygen atoms ability to stabilize the intermediate formed when the α-hydrogens are abstracted. Alcoholic solvents (MeOH, EtOH, *i*PrOH and BuOH) were found to be far less effective, it is suspected they are not sufficiently active as hydride radical sources or more likely that they react with the unstable intermediates in the mechanism, due to their nucleophilicity. Correlations between the ethers are not readily explained with the current available data. It is unfortunate that cyclohexane, a good hydride donor, could not be fully tested because the starting materials were not particularly soluble, however, some success was achieved when refluxing in batch. Of general note was that in all experiments the starting materials were completely consumed, therefore we turned our attention to measuring the efficiency of the process in terms of isolated yield rather than the kinetics.

### 2.2. Temperature Screening

Even substantially deactivated anilines, such as nitro-substituted, and amino-heterocycles will react with inorganic nitrite in aqueous conditions without external heating, even below room temperature, because the active nitrosation agent (NO^+^) forms in situ. When using organic nitrites, some thermal input is generally required to overcome the greater energy barrier of the first nucleophilic attack. This is normally the rate-determining step as the subsequent intermediates are never detected and the process is noticeably more efficient at higher temperatures (Table 2 and Table 3). For this evaluation we also tested 1,4-dioxane even though it had displayed reduced comparative reactivity as this solvent could be used at a higher temperature but at reduced internal reactor pressure.

### 2.3. Coil Size and Residence Time

It was initially anticipated that a shorter coil volume may improve the yield along with throughput, as the product might begin to decompose after it has formed. Several thiazoles, oxazoles and related heterocycles are known to undergo side reactions via ring opening process such as the Cornforth rearrangement. However, the obtained results did not support this hypothesis, indeed a longer residence time gave improved conversion at the elevated temperature. It is therefore unlikely that the thiazole product degrades significantly under the conditions of this process (Table 4).

### 2.4. The Application of Radical Catalysts

Perretti et al. as well as Felipe-Blanco et al. have shown that using a nucleophilic compound that can form stable radicals can increase the rates and yields of dediazoniation processes, by lowering the energy barrier to forming the target aryl radical species, hence reducing the chances of side transformations [6,7]. We therefore selected a poorly reactive substrate to evaluate any potential advantage of using such an approach in combination with flow processing (Table 5). There was a notable enhancement in conversion obtained when employing the catalyst however, at elevated temperatures this effect was not observed and hence we decided to concentrate on temperature rather than catalyst application. It should be noted that this may still offer benefit as an approach for poorly reactive substrates which give turnover at lower temperatures. 

### 2.5. Different Organic Nitrite Sources

Several organic nitrites were screened for their effectiveness in this process; tested were tertiary-butyl nitrite, normal-butyl nitrite and *iso*-propyl nitrite. All were found to perform similarly, with all giving near quantitative conversions for substrates, such as 2-aminobenzothiazole (Table 6, **2s**). Based upon a consideration of its lower cost, the *iso*-pentyl nitrite was selected as the reagent of choice for all further studies (Table 6).

### 2.6. Substrate Scope for Optimised Process

The flow procedure was applied to a variety of substrates, from simple amino-pyridines to sophisticated, multi-cyclic structures. Most substrates were deaminated efficiently, giving good-to-excellent conversions and isolated yields, after column chromatography purification. Although some substrates proceeded in lower yields, other studies have failed to deaminate these substrates at all.

## 3. Discussion

The fast reaction kinetics of the hydrodediazoniation ensures that full consumption of the starting material was observed for almost every derivative. The reason for imperfect yields was because of working with very unstable intermediates; namely the hetero-diazonium and the heteroaryl radical. For example, the methyl 2-diazoniumthiazole-5-carboxylate tetrafluoroborate salt was stable at −5 °C (though highly dependent on the anion), but the aryl radical was never detected and attempts to trap it with a stable radical, such as TEMPO were not successful, although more stable nitrobenzene aryl radicals have been trapped by Perretti et al. [6]. The key to maximising yields for the process is therefore dependant on optimizing the process to limit decomposition pathways from both reactive intermediates.

**Scheme 1:**
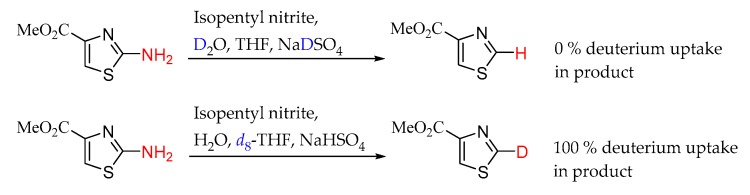
The results from experiments using deuterated solvents, to show that the new hydrogen always comes from the alkyl tetrahydrofuran (THF) hydrogens and not acidic protons from the solvent.

In general, the diazonium intermediate can become a more stable structure by emitting N_2_. Nucleophilic substitution, with N_2_ as the leaving group is sometimes possible for diazonium compounds, but is not specifically observed in this reaction. Our experiments with deuterated THF show that the new hydrogen in the product always comes from the THF, hence ruling out any possibility of nucleophilic behaviour (Scheme 1).

**Figure 2:**
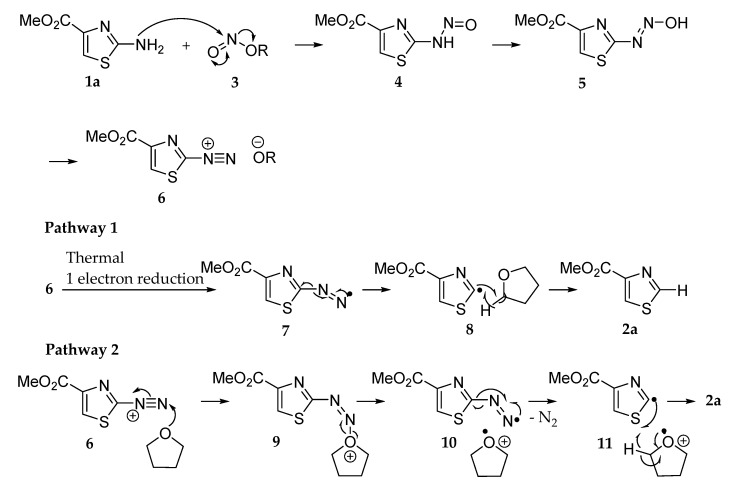
The postulated mechanism for the formation of the aryl radical and termination to the target hydrogenated product.

To enable radical hydride abstraction the diazonium compound **6** must undergo a one-electron reduction to become the radical species **7**, which can eliminate N_2_ to form the aryl radical **8** (Figure 2). There is much debate regarding how this may occur, it could be a thermal process, particularly at the elevated reaction temperatures used, or even photochemically, from the ambient lighting within the laboratory (Figure 2, pathway 1) [17]. However, photochemical activation as the primary route has been ruled out by performing the process with the exclusion of light. Alternatively, it is known that nucleophilic solvents, from alcohols to cyclic ethers, can interact with the diazo group, forming a positively charged intermediate **9** that can fragment radically, leading again to the aryl radical **8** (Figure 2, pathway 2). The results obtained in this study cannot distinguish which pathways are under operation, although it was shown that neither are possible at 0 °C, as there is no reaction between the thiazole diazonium compound and THF at this temperature unless additional sodium nitrite is added (which is known to convert some diazonium compounds to aryl radicals) [9].

The versatility of the deamination process comes from the fact that the conversion is rapid under conditions where most other functionalities are stable and it can be run as a single step process (diazotization and substitution). Advantageously, a mild pH is used and there is no need for additional radical initiators or catalysts that could cause compatibility issues. The ether solvents of choice are inert, relatively low cost and readily available. The process is very simple and easy to perform, adding to its value as part of multi-stage syntheses, particularly as the aromatic nitrogen is often residual from the heterocyclic formation. Consequently, the flow process described has been specifically designed to target heteroaryl amines that are poorer nucleophiles and generally do not form stable diazonium salts, hence limiting the yield of any segregated process that relies on first forming the diazonium salt then, for example, conducting a hydrogenation in a subsequent step. 

**Figure 3:**
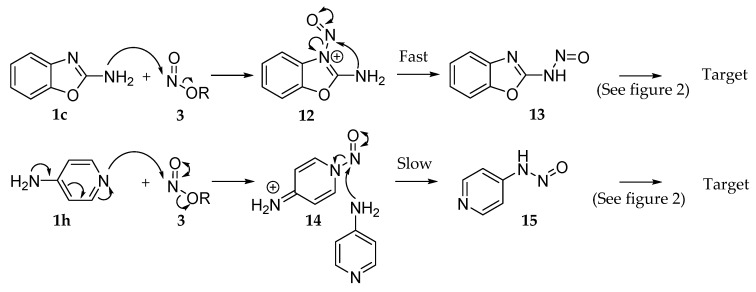
A potential explanation as to why some substrates react more effectively under the presented procedure than others.

*N*-containing heteroaromatics often present a challenge in a range of nucleophilic processes targeting the primary amine, including diazotisation, due to the inherent low nucleophilicity of the heterocyclic *N* atom. To the best of our knowledge, this process is the first published procedure for successful hydrodeazoniation of aminopyridines. The difficulty arises because the electrophile (isopentyl nitrite in this case) can react, often preferentially, with the heterocyclic *N* atom. While this addition is largely reversible, the nitroso group must migrate to the primary amine in order for the diazotization to proceed (Figure 3). This is fairly trivial for cycles where the *N* atom (or atoms) is in close proximity to the primary amine and if the primary amine is sufficiently nucleophilic (e.g., 2-aminobenzimidazole **1b**, Figure 3), allowing for swift intramolecular transfer to the target primary amine. For substrates such as 4-aminopyridine **1h**, this is not the case and these substrates likely rely on intermolecular transfer to the primary amine (Figure 3). The intermediate nitrosoamine **14**, formed in an attack from the heterocyclic *N* on the isopentenyl nitrite, may not be stable under these conditions and hence this represents another pathway to decomposition and yield loss and could explain why 3-aminopyridine **2g** was deaminated in much higher yield than the 4-amino **1h** (Table 1). While the migration of the nitroso group from the pyridine *N* atom to the primary amine is not hindered for 2-aminopyridine **2f**, the weak nucleophilicity of the primary amine ensures that this substrate could also only be deaminated in poor conversions (Table 6).

Although the process was successful for a range of heterocycles, unfortunately deamination was not possible for certain compound types (Table 6) in which the starting materials were recovered unchanged. The aminopyrimidines which were unreactive possess very low nucleophilicity at the primary amine as well as having increased steric hindrance around the nitrogen [18]. We also prepared the ammonium salt **2aq** to test if increased acidity may help promote the process but without advantage. However, we were able to show that compound **2z** possessing both a primary pyrazole and pyrimidine based *N* atom underwent deamination selectively only at the pyrazole centred nitrogen albeit in low yield (Table 6). We hope to explore this reaction more and find ways to elaborate the less reactive structures as well as explore the interesting natural selectivity in other species.

## 4. Materials and Methods 

Reagents and solvents were purchased from Fischer Scientific, Sigma Aldrich™, Fluorochem™, or Alfa Aesar™, were of analytical reagent grade and were used as received. ^1^H and ^13^C-NMR spectra were recorded, in specified deuterated solvents, (purchased from Apollo Scientific™), at room temperature on Bruker™ Avance-400 (^1^H, ^13^C) (operating at 400.13 MHz) spectrometers and are reported as follows: chemical shift δ (ppm) (number of protons, multiplicity, coupling constant J (Hz) (if applicable), assignment). Multiplicities are reported using the following abbreviations: s (singlet), d (doublet), t (triplet), q (quartet) and m (unresolved multiplet). All ^13^C-NMR spectra were proton-decoupled and carbons are numbered according to the IUPAC systematic name. The ^1^H and ^13^C chemical shifts are reported using the residual signal of deuterated solvent as the internal reference (for CDCl_3_: δ_H_ = 7.26 ppm; δ_C_ = 77.16 ppm and for deuterated *d*^6^-DMSO: δ_H_ = 2.50 ppm; δ_C_ = 39.51 ppm). All chemical shifts are quoted in parts per million, relative to tetramethylsilane (δ_H_, δ_C_ = 0.00 ppm). All coupling constants are ^3^J_HH_ unless otherwise stated. Electrospray Ionisation (ESI) mass spectra, for LC-MS results were obtained using a TQD mass spectrometer (Waters UK Ltd., Manchester, UK). High-resolution mass spectra were obtained with an LCT Premier XE mass spectrometer (Waters UK Ltd., Manchester, UK); all were obtained by the Durham University Mass Spectrometry service. ASAP mass spectra were obtained using a Waters™ Synapt G2s apparatus. Thin-Layer Chromatography was performed using Merck TLC Aluminium oxide 60 F254 with glass backing. Plates were stained with potassium permanganate solution, where required and visualised using UV light. Column chromatography refers to purification by applying the mixture, dissolved in a minimum amount of dichloromethane, onto silica gel (40–63 µm mesh size) with the stated solvent system.

### 4.1. General Procedure for Solvent Screening

A solution of methyl 2-aminothophene-5-carboxylate (152 mg, 1.00 mmol) in the selected solvent (see Table 1, 10 mL) and a solution of isopentyl nitrite (141 mg, 1.20 mmol) in the selected solvent (10 mL) were both pumped at a flow rate of 0.25 mL min^−1^ with a Vapourtech ‘Easy MedChem V3’ system, meeting at a PTFE T-piece and the output flowing through a 6.5 mL coil reactor maintained at 70 °C, giving a residence time of 13 min (see Table 2). The pressure of the system was maintained at 7 bar with a variable compression back-pressure regulator. The output mixture was concentrated under reduced pressure (100 mbar) to yield the product as an oil. Three repeat samples were analysed by ^1^H-NMR spectroscopy with an internal standard (nitrobenzene) and the average used to quantify the conversion to the target thiazole product.

### 4.2. General Procedure for Temperature Investigation

A solution of methyl 2-aminothiazole-5-carboxylate (152 mg, 1.00 mmol) in THF (10 mL) and a solution of isopentyl nitrite (141 mg, 1.20 mmol) in THF (10 mL) were both pumped at a flow rate of 0.25 mL min^−1^ with a Vapourtech ‘Easy MedChem V3’ system, meeting at a T-piece and the output flowing through a 10 mL coil reactor maintained at the selected temperature (see Table 3 and Table 4), giving a residence time of 20 min. The pressure of the system was maintained at 7 bar with a back-pressure regulator. The output mixture was concentrated under reduced pressure (100 mbar) to yield the product as an oil. Three repeat samples were analysed by ^1^H-NMR spectroscopy with an internal standard (nitrobenzene) and the average used to quantify the conversion to the target thiophene product.

### 4.3. General Procedure for Reactor Coil Size Investigation

A solution of methyl 2-aminothiazole-5-carboxylate (152 mg, 1.00 mmol) in THF (10 mL) and a solution of isopentyl nitrite (141 mg, 1.20 mmol) in THF (10 mL) were both pumped at a flow rate of 0.25 mL min^−1^ with a Vapourtech ‘Easy MedChem V3’ system, meeting at a T-piece and the output flowing through the stated coil reactor maintained at the selected temperature (see Table 4). The pressure of the system was maintained at 7 bar with a back-pressure regulator. The output mixture was concentrated under reduced pressure (100 mbar) to yield the product as an oil. Three repeat samples were analysed by ^1^H-NMR spectroscopy with an internal standard (nitrobenzene) and the average used to quantify the conversion to the target thiazole product.

### 4.4. General Procedure for Investigation of Catalysis using Gallic Acid and Salicylic Acid

A solution of 4-(5-amino-4-cyano-1*H*-pyrazol-1-yl)-benzoic acid (228 mg, 1.00 mmol) and isopentyl nitrite (141 mg, 1.20 mmol) with the stated catalyst loading of salicylic acid or gallic acid (see Table 5) in THF (50 mL) was pumped at a flow rate of 1.0 mL min^−1^ with a Vapourtech ‘Easy MedChem V3’ system flowing through a 10 mL coil reactor maintained at 70 °C, giving a residence time of 20 min. The pressure of the system was maintained at 7 bar with a back-pressure regulator. The output mixture was concentrated under reduced pressure to give a powder. Three repeat samples were analysed by ^1^H-NMR spectroscopy with an internal standard (nitrobenzene) and the average used to quantify the conversion of the target product.

### 4.5. General Procedure for Investigation of Alternative Organic Nitrites

A solution of methyl 2-aminothiazole-5-carboxylate (152 mg, 1.00 mmol) in THF (10 mL) and the stated organic nitrite (1.20 mmol) (see Table 6) in THF (10 mL) were pumped at a flow rate of 0.25 mL min^−1^ with a Vapourtech ‘Easy MedChem V3’ system, meeting at a T piece, then flowing through a 10 mL coil reactor maintained at 120 °C, giving a residence time of 10 min. The pressure of the system was maintained at 7 bar with a back-pressure regulator. The output mixture was concentrated under reduced pressure (100 mbar) to yield the product as an oil. Three repeat samples were analysed by ^1^H-NMR spectroscopy with an internal standard (nitrobenzene) and the average used to quantify the conversion of the target product.

### 4.6. General Procedure for Hydrodeazoniation of Substrates

A solution of the selected heterocyclic starting material (1.00 mmol) in THF (10 mL) and a solution of isopentyl nitrite (141 mg, 1.20 mmol) in THF (10 mL) were both pumped at a flow rate of 0.25 mL min^−1^ with a Vapourtech ‘Easy MedChem V3’ system, meeting at a PTFE T-piece and the output flowing through a 10.0 mL coil reactor maintained at 120 °C, giving a residence time of 20 min. The pressure of the system was maintained at 7 bar with a back-pressure regulator. For compounds where an isolated yield was reported: the output mixture was concentrated under reduced pressure to give an oil (or powder). The oil (or powder) was purified using column chromatography with various mixtures of ethyl acetate and hexane as the eluent, or by recrystallisation using methanol, to give isolated compounds that showed no impurities by NMR spectroscopy. For compounds where a conversion was reported (due to volatility of products), the output mixture was carefully concentrated under a reduced pressure of 100 mbar for 10 min and the conversion was calculated by integration of product peaks to a quantified internal standard (nitrobenzene).

Starting materials **1a**–**d**, **1f**–**s, 1al**–**ar** were obtained from Alfa Asear and were used as supplied without additional purification. Other starting materials were synthesised, with spectra provided in the supplementary information.

*6-Chlorobenzo[1,3]thiazole* (**2d**): Eluent: hexane/ethyl acetate (100:1)**→**(4:1) yellow crystals, 68% yield. ^1^H-NMR (400 MHz, CDCl_3_) δ 8.98 (s, 1H), 8.05 (d, *J* = 8.7 Hz, 1H), 7.95 (d, *J* = 2.1 Hz, 1H), 7.49 (dd, *J* = 8.7, 2.1 Hz, 1H). ^13^C-NMR (101 MHz, CDCl_3_) δ 154.51 (CH), 151.72 (C), 135.06 (C), 131.95 (C), 127.34 (CH), 124.46 (CH), 121.66 (CH). ASAP-MS (MeCN) R_t_ = 0.50 min [M + H]^+^ = 170.0.

*3-(1-Methylethyl)benzoic acid* (**2e**): Eluent: hexane/ethyl acetate (100:1)**→**(4:1). Yellow needles, 96% yield. ^1^H-NMR (400 MHz, CDCl_3_) δ 8.00 (d, *J* = 1.8 Hz, 1H), 7.95 (dt, *J* = 7.6, 1.4 Hz, 1H), 7.48 (d, *J* = 1.3 Hz, 1H), 7.40 (t, *J* = 1.3 Hz, 1H), 2.99 (p, *J* = 7.3 Hz, 1H), 1.29 (d, *J* = 7.3 Hz, 6H). ^13^C-NMR (101 MHz, CDCl_3_) δ 172.87 (C), 149.44 (C), 132.29 (CH), 129.46 (C), 128.63 (CH), 128.34 (CH), 127.93 (CH), 34.16 (CH), 24.01 (CH_3_). LC-MS (MeCN) R_t_ = 2.25 min [M − H]^−^ = 163.2. HRMS C_10_H_11_O_2_ calc. 163.0759, found 163.0754, (Δ = −3.1 ppm).

*Methyl thiophene-3-carboxylate* (**2r**): Eluent: hexane/ethyl acetate (100:1)**→**(10:1). ^1^H-NMR (400 MHz, CDCl_3_) δ 8.12 (dd, *J* = 3.0, 1.2 Hz, 1H), 7.54 (dd, *J* = 5.1, 1.2 Hz, 1H), 7.41–7.28 (m, 1H), 3.95–3.85 (m, 3H). ^13^C-NMR (101 MHz, CDCl_3_) δ 161.28, 133.50, 132.70, 127.88, 126.04, 51.84. LC-MS (MeOH) R_t_ = 0.87 min [M + H]^+^ = 143.1.

*Benzo[1,3]thiazole* (**2s**): Eluent: hexane/ethyl acetate (100:1)**→**(10:1). Brown, volatile oil, 99% yield. ^1^H-NMR (400 MHz, CDCl_3_) δ = 9.00 (s, 1H), 8.35 (d, *J* = 8.2 Hz, 1H), 7.95 (d, *J* = 8.2 Hz, 1H), 7.51 (t, *J* = 7.7 Hz, 1H), 7.42 (t, *J* = 7.7 Hz, 1H). GC-MS M^+^ 135.1.

*5-(2-Bromophenyl)-1,3,4-thiadiazol-2-amine* (**1t**): Starting material **1t** was prepared following the procedure of Mullick et al. [19] and isolated as a white solid in 85% yield. ^1^H-NMR (400 MHz, CDCl_3_) δ 8.01 (dd, *J* = 7.8, 1.7 Hz, 1H), 7.70 (dd, *J* = 7.9, 1.3 Hz, 1H), 7.43 (td, *J* = 7.6, 1.3 Hz, 1H), 7.37–7.29 (m, 1H), 6.06 (br. s, 2H). ^1^H-NMR (400 MHz, DMSO-*d*_6_) δ 7.87 (dd, *J* = 7.8, 1.8 Hz, 1H), 7.75 (dd, *J* = 7.8, 1.2 Hz, 1H), 7.53–7.41 (m, 3H), 7.37 (td, *J* = 7.8, 1.8 Hz, 1H). ^13^C-NMR (101 MHz, DMSO-*d_6_*) δ 170.47 (C), 153.62 (C), 134.17 (C), 132.07 (C), 131.65 (C), 128.53 (C), 121.38 (C). ^13^C-NMR (101 MHz, DMSO*-d_6_*) δ 134.17, 131.66, 131.64, 128.53. LC-MS (MeCN) R_t_ = 1.69 min [M + H]^+^ = 258.2. HRMS C_8_H_6_N_3_S^79^Br calc. 255.9544, found 255.9551, (Δ = 2.7 ppm).

*2-(2-Bromophenyl)-1,3,4-thiadiazole* (**2t**): Eluent: hexane/ethyl acetate (95:5)**→**(4:1). Brown oil, 67% yield. ^1^H-NMR (400 MHz, CDCl_3_) δ 9.25 (s, 1H), 8.14 (d, *J* = 7.8 Hz, 1H), 7.74 (d, *J* = 8.0 Hz, 1H), 7.46 (t, *J* = 7.6 Hz, 1H), 7.36 (t, *J* = 7.5 Hz, 1H). ^13^C-NMR (101 MHz, CDCl_3_) δ 165.25 (C), 152.87 (CH), 134.12 (CH), 132.21 (CH), 132.01 (CH), 130.67 (C), 128.03 (CH), 122.71 (C). 

*4-(4-Cyano-1H-pyrazol-1-yl)-benzoic acid* (**2u**): [20] Starting material **1u** was prepared following the procedure of Smith et al. [21]. The product was obtained via recrystallization from methanol. Purple powder, 70% yield. ^1^H-NMR (400 MHz, DMSO-*d*_6_) δ 13.19 (s, 1H), 9.46 (s, 1H), 8.43 (s, 1H), 8.10 (d, *J* = 8.8 Hz, 2H), 8.00 (d, *J* = 8.8 Hz, 2H). ^13^C-NMR (101 MHz, DMSO-*d*_6_) δ 143.56 (CH), 138.89 (C), 134.24 (CH), 130.34 (CH), 118.24 (CH), 113.65 (C), 93.07 (C), 51.03 (CH). LC-MS (MeCN) R_t_ = 1.67 min [M − H]^–^ = 212.2. HRMS C_11_H_6_N_3_O_2_ calc. 212.0460, found 212.0456, (Δ = −1.9 ppm).

*1-(4-Fluorophenyl)-1H-pyrazole-4-carbonitrile* (**2v**): [22,23] Starting material **1v** was prepared following the procedure of Smith et al. [21]. The product was obtained via recrystallization from ethanol and water. Brown crystals, 77% yield. ^1^H-NMR (400 MHz, DMSO-*d*_6_) δ 9.28 (s, 1H), 8.33 (s, 1H), 8.00–7.73 (m, 2H), 7.52–7.16 (m, 2H). ^13^C-NMR (101 MHz, DMSO-*d*_6_) δ161.62 (C, d, *J* = 245.8 Hz), 143.96 (CH), 135.47 (C, d, *J* = 3.0 Hz), 134.75 (CH), 122.01 (CH, d, *J* = 9.0 Hz), 116.93 (CH, d, *J* = 23.8 Hz), 113.97 (C), 93.59 (C). ^19^F NMR (376 MHz, DMSO-*d*_6_) δ −114.22. GC-MS (MeCN) R_t_ = 0.30 min M^+^ = 187.1. HRMS C_10_H_7_N_3_F calc. 188.0624, found 188.0615, (Δ = −4.8 ppm).

*1-(3-Chloro-4-fluorophenyl)-1H-pyrazole-4-carbonitrile* (**2w**): Starting material **1w** was prepared following the procedure of Smith et al. [21]. The product was obtained via recrystallization ethanol and water. Brown solid, 69% yield. ^1^H-NMR (400 MHz, DMSO-*d*_6_) δ 9.35 (s, 1H), 8.39 (s, 1H), 8.13 (dt, *J* = 6.3, 2.0 Hz, 1H), 7.98–7.76 (m, 1H), 7.64 (td, *J* = 9.0, 1.2 Hz, 1H)). ^13^C-NMR (101 MHz, DMSO-*d*_6_) δ 158.84 (C, d, *J* = 247.1 Hz), 144.32 (CH), 135.93 (C, d, *J* = 3.2 Hz), 135.26 (CH), 122.05 (CH), 121.09 (C, d, *J* = 19.3 Hz), 120.58 (CH, d, *J* = 8.0 Hz), 118.46 (CH, d, *J* = 22.4 Hz), 113.83 (C), 93.92 (C). ^19^F NMR (376 MHz, DMSO-*d*_6_) δ −117.41. ASAP-MS (MeCN) R_t_ = 0.30 min [M + H]^+^ = 222.0. HRMS C_10_H_6_Cl_2_FN_3_ calc. 222.0234, found 222.0228, (Δ = −2.7 ppm).

*5-Amino-1-(6-methyl-4-(trifluoromethyl)pyridin-2-yl)-1H-pyrazole-4-carbonitrile* (**1x**): Starting material **1x** was prepared following the procedure of Smith et al. [21]. ^1^H-NMR (400 MHz, DMSO-*d*_6_) δ 8.09 (s, 2H), 8.01–7.87 (m, 1H), 7.87–7.75 (m, 1H), 7.59 (s, 1H), 2.64 (s, 3H). ^13^C-NMR (101 MHz, DMSO-*d*_6_) δ 159.72 (C), 153.73 (C), 153.58 (C), 143.73 (CH), 140.25 (C, q, *J* = 33.9 Hz), 122.90 (C, q, *J* = 273.5 Hz), 116.23 (CH, q, *J* = 3.2 Hz), 114.65 (C), 106.40 (CH, q, *J* = 3.2 Hz), 73.92 (C), 24.09 (CH3). ^19^F NMR (376 MHz, DMSO-*d*_6_) δ 63.81. LC-MS (MeCN) R_t_ = 2.22 min [M + H]^+^ = 269.2.

*1-(6-Methyl-4-(trifluoromethyl)pyridin-2-yl)-1H-pyrazole-4-carbonitrile* (**2x**): Recrystallised from ethanol and water as rose coloured needles in 76% yield. ^1^H-NMR (400 MHz, DMSO-*d*_6_) δ 9.43 (s, 1H), 8.47 (s, 1H), 7.95 (s, 1H), 7.78 (s, 1H), 2.65 (s, 3H). ^13^C-NMR (101 MHz, DMSO-*d*_6_) δ 160.98 (C), 150.29 (C), 145.27 (CH), 140.48 (C, q, *J* = 34.6 Hz), 134.47 (CH), 122.84 (C, q, *J* = 273.5 Hz), 119.01 (CH, q, *J* = 3.4 Hz), 113.62 (C), 106.15 (CH, q, *J* = 4.0 Hz), 94.81 (C), 24.30 (CH_3_). ^19^F NMR (376 MHz, DMSO-*d*_6_) δ −63.60. LC-MS (MeCN) R_t_ = 4.44 min M^+^ = 252.1. HRMS C_11_H_8_N_4_F_3_ calc. 253.0701, found 253.0702, (Δ = 0.4 ppm).

*4-(4-Fluorophenyl)-6-phenylpyrimidin-2-amine* (**1y**): Starting material **1y** was prepared following the procedure of Baxendale et al. [24]. Recrystallised from ethanol and obtained as yellow crystals, 59% yield. ^1^H-NMR (400 MHz, DMSO-*d*_6_) δ 8.29–8.26 (m, 2H), 8.17–8.15 (m, 2H), 7.67–7.58 (m, 4H), 7.47–7.42 (m, 2H). ^13^C-NMR (101 MHz, DMSO-*d*_6_) δ 165.81 (C), 164.50 (C, d, *J* = 29.7 Hz), 163.32 (C), 157.53 (C), 133.17 (C), 132.22 (CH), 130.68 (CH, d, *J* = 9.2 Hz), 130.24 (CH), 128.95 (CH), 127.98 (CH), 116.10 (CH, d, *J* = 21.9 Hz), 100.81 (CH), 30.69 (CH). ^19^F NMR (376 MHz, DMSO-*d*_6_) δ −107.72. ASAP-MS (MeCN) R_t_ = 0.36 min [M + H]^+^ = 267.1. Product 4-(4-fluorophenyl)-6-phenylpyrimidine (**2y**) was consistent with the literature data [25]. LC-MS (MeCN) R_t_ = 1.96 min [M + H]^+^ = 251.3. HRMS C_16_H_12_N_2_F calc. 251.0985, found 251.0982, (Δ = −1.2 ppm).

*1-Phenyl-6-(1-phenyl-1H-pyrazol-4-yl)-1H-pyrazolo[3,4-d]pyrimidin-4-amine* (**2z**): Starting material **1z** was prepared following the procedure of Smith et al. [21]. Eluent: hexane/ethyl acetate (4:1)**→**(1:4). Orange solid, 30% yield. ^1^H-NMR (400 MHz, DMSO-*d*_6_) δ ^13^C-NMR (101 MHz, DMSO-*d*_6_) δ 158.24, 154.27, 148.34, 141.13, 139.40, 139.28, 134.19, 129.59, 129.32, 129.24, 128.41, 128.07, 126.76, 125.73, 125.35, 123.34, 120.28, 118.81, 100.03. LC-MS (MeCN) R_t_ = 2.76 min [M + H]^+^ = 352.3. HRMS C_22_H_19_N_5_ calc. 354.1470, found 354.1476, (Δ = 1.7 ppm).

*1-Phenyl-1H-1,2,3-triazole-4-carbonitrile* (**2aa**): [26] Starting material **1aa** was prepared following the procedure of Smith et al. [27]. The product was obtained by recrystallization from ethanol and water. Orange solid, 65% yield. ^1^H-NMR (400 MHz, DMSO-*d*_6_) δ 8.51 (s, 1H), 7.77–7.74 (m, 2H), 7.64–7.55 (m, 3H). ^13^C-NMR (101 MHz, DMSO-*d*_6_) δ 135.74 (C), 130.21 (CH), 127.59 (CH), 121.96 (C), 120.99 (CH), 111.21 (C).

*1-(p-Tolyl)-1H-1,2,3-triazole-4-carbonitrile* (**2ab**): [28] Starting material **1ab** was prepared following the procedure of Smith et al. [27]. The product was obtained by recrystallization from ethanol and water. Brown solid, 71% yield. ^1^H-NMR (400 MHz, DMSO-*d*_6_) δ 9.69 (s, 1H), 7.80 (app. d, *J* = 8.5 Hz, 2H), 7.45 (app. d, *J* = 8.5 Hz, 2H), 2.40 (s, 3H). ^13^C-NMR (101 MHz, DMSO-*d*_6_) δ 140.22 (C), 133.82 (C), 130.89 (C), 130.85 (CH), 121.22 (CH), 120.80 (C), 112.52 (C), 21.11 (CH_3_). ASAP-MS (MeCN) R_t_ = 0.47 min [M + H]^+^ = 185.1. HRMS C_10_H_9_N_4_ calc. 185.0827, found 185.0830, (Δ = 1.6 ppm).

*1-(4-Nitrophenyl)-1H-1,2,3-triazole-4-carbonitrile* (**2ac**): Starting material **1ac** was prepared following the procedure of Smith et al. [27]. The product was obtained by recrystallization from ethanol and water as a brown solid in 81% yield. ^1^H-NMR (400 MHz, DMSO-*d*_6_) δ 9.94 (s, 1H), 8.51 (d, *J* = 8.7 Hz, 2H), 8.23 (d, *J* = 8.7 Hz, 2H). ^13^C-NMR (101 MHz, DMSO-*d*_6_) δ 148.03 (C), 140.27 (C), 131.81 (CH), 126.08 (CH), 122.21 (CH), 121.35 (C), 112.19 (C). ASAP-MS (MeCN) R_t_ = 0.75 min [M + H]^+^ = 216.1. HRMS C_9_H_6_N_5_O_2_ calc. 216.0521, found 216.0521, (Δ = 0.0 ppm).

*2-(2-Pyridinyl)-quinoline* (**2ad**): [29] Starting material **1z** was prepared following the procedure of Smith et al. [30]. The product was obtained by elution with hexane/ethyl acetate (100:1)**→**(10:1) as a white solid in 70% yield. ^1^H-NMR (400 MHz, CDCl_3_) δ 8.75 (ddd, *J* = 4.8, 1.8, 1.0 Hz, 1H), 8.68 (dt, *J* = 8.0, 1.1 Hz, 1H), 8.58 (d, *J* = 8.6 Hz, 1H), 8.29 (dd, *J* = 8.6, 0.9 Hz, 1H), 8.21 (dq, *J* = 8.5, 0.9 Hz, 1H), 7.95–7.81 (m, 2H), 7.76 (ddd, *J* = 8.4, 6.9, 1.5 Hz, 1H), 7.57 (ddd, *J* = 8.1, 6.9, 1.2 Hz, 1H), 7.37 (ddd, *J* = 7.5, 4.8, 1.2 Hz, 1H). ^13^C-NMR (101 MHz, CDCl_3_) δ 156.17 (C, d, *J* = 16.0 Hz), 149.24 (CH), 147.96 (C), 137.13 (CH, d, *J* = 12.2 Hz), 129.72 (CH, d, *J* = 15.5 Hz), 128.37 (C), 127.74 (CH), 126.91 (CH), 124.18 (CH), 122.02 (CH), 119.09 (CH). ASAP-MS (MeCN) R_t_ = 0.59 min [M + H]^+^ = 207.1.

*(2-Aminothiophen-3-yl)(phenyl)methanone* (**1ae**): Starting material **1ae** was prepared following the procedure of Mallia et al. [31] as tan needles in 99% yield by column chromatography using hexane/ethyl acetate (1:1). ^1^H-NMR (400 MHz, DMSO-*d*_6_) δ 8.37 (s, 2H), 7.64–7.55 (m, 2H), 7.55–7.41 (m, 3H), 6.74 (d, *J* = 5.9 Hz, 1H), 6.27 (d, *J* = 5.9 Hz, 1H). ^13^C-NMR (101 MHz, DMSO-*d*_6_) δ 189.62 (C), 167.83 (C), 141.38 (C), 130.92 (CH), 128.68 (CH), 128.14 (CH), 127.06 (CH), 113.37 (C), 106.66 (CH). LC-MS (MeCN) R_t_ = 2.20 min [M + H]^+^ = 204.2. HRMS C_11_H_10_NOS calc. 204.0483, found 204.0484, (Δ = 0.5 ppm).

*Phenyl(thiophen-3-yl)methanone* (**2ae**): [32] Eluent: hexane/ethyl acetate (1:1) as a tan coloured oil in 66% yield. ^1^H-NMR (400 MHz, DMSO-*d*_6_) δ 8.21 (dd, *J* = 2.8, 1.3 Hz, 1H), 7.83–7.77 (m, 2H), 7.71 (dd, *J* = 5.0, 2.8 Hz, 1H), 7.69–7.62 (m, 1H), 7.59–7.50 (m, 3H). ^13^C-NMR (101 MHz, DMSO-*d*_6_) δ 189.57 (C), 140.87 (C), 138.56 (C), 135.73 (CH), 132.91 (CH), 129.50 (CH), 129.05 (CH), 128.50 (CH), 128.05 (CH). LC-MS (MeCN) R_t_ = 2.41 min [M + H]^+^ = 189.2. HRMS C_11_H_9_OS calc. 189.0374, found 189.0377, (Δ = 1.6 ppm).

*(2-Aminothiophen-3-yl)(4-methoxyphenyl)methanone* (**1af**): Starting material **1xx** was prepared following the procedure of Mallia et al. [31] as tan needles in 99% yield by column chromatography using hexane/ethyl acetate (1:1). ^1^H-NMR (400 MHz, DMSO-*d*_6_) δ 8.25 (s, 2H), 7.60 (d, *J* = 8.25 Hz, 2H), 7.02 (d, *J* = 8.25 Hz, 2H), 6.81 (d, *J* = 5.9 Hz, 1H), 6.27 (d, *J* = 5.9 Hz, 1H), 3.82 (s, 3H). ^13^C-NMR (101 MHz, DMSO-*d*_6_) δ 188.72 (C), 167.35 (C), 161.61 (C), 133.73 (C), 130.38 (CH), 127.16 (CH), 113.93 (CH), 113.40 (C), 106.43 (CH), 55.78 (CH_3_). LC-MS (MeCN) R_t_ = 2.20 min [M + H]^+^ = 234.2. HRMS C_12_H_12_NO_2_S calc. 234.0589, found 234.0594, (Δ = 2.1 ppm).

*(4-Methoxyphenyl)(thiophen-3-yl)methanone* (**2af**): [33] Eluent: hexane/ethyl acetate (4:1). Yellow crystals, 69% yield. ^1^H-NMR (400 MHz, DMSO-*d*_6_) δ 8.18 (dd, *J* = 2.8, 1.3 Hz, 1H), 7.86–7.77 (m, 2H), 7.70 (dd, *J* = 5.1, 2.8 Hz, 1H), 7.50 (dd, *J* = 5.1, 1.3 Hz, 1H), 7.14–7.02 (m, 2H), 3.86 (s, 3H). ^13^C-NMR (101 MHz, DMSO-*d*_6_) δ 188.26 (C), 163.27 (C), 141.15 (C), 134.32 (CH), 132.01 (CH), 130.92 (C), 128.63 (CH), 127.74 (CH), 114.35 (CH), 55.99 (CH_3_). LC-MS (MeCN) R_t_ = 2.41 min [M + H]^+^ = 219.4 HRMS C_12_H_10_O_2_S calc. 219.0480, found 219.0486, (Δ = 2.7 ppm).

*4-(3-chlorophenyl)thiazol-2-amine* (**1ag**): Prepared following the procedure of Potopnyk et al. [34]. Recrystallised from acetone to yield a white solid, 90% yield. ^1^H-NMR (400 MHz, DMSO-*d*_6_) δ 7.85 (t, *J* = 2.0 Hz, 1H), 7.72 (dt, *J* = 7.3, 1.7 Hz, 1H), 7.56–7.44 (m, 2H), 7.40 (s, 1H). ^13^C-NMR (101 MHz, DMSO-*d*_6_) δ 170.49 (C), 139.07 (C), 134.19 (C), 131.74 (CH), 131.33 (C), 129.39 (CH), 125.98 (CH), 124.94 (C), 104.93 (CH). LC-MS (MeCN) R_t_ = 1.25 min [M + H]^+^ = 211.0. HRMS C_9_H_7_ClN_2_S calc. 211.0097, found 211.0111, (Δ = 6.6 ppm).

*4-(3-chlorophenyl)thiazole* (**2ag**): Isolated as an inseparable mixture of the product and a secondary species in a ratio of 1:0.33 (**2ag**:by-product) equating to 45% of the desired product.

*4-(3-bromophenyl)thiazol-2-amine* (**1ah**): Prepared following the procedure of Potopnyk et al. [34]. Recrystallised from acetone to yield a white solid. White crystals, 88% yield. ^1^H-NMR (400 MHz, DMSO-*d*_6_) δ 9.04 (s, 2H), 7.97 (t, *J* = 1.8 Hz, 1H), 7.76 (dt, *J* = 7.7, 1.2 Hz, 1H), 7.61 (dt, *J* = 7.7, 1.8 Hz, 1H), 7.52–7.29 (m, 2H). ^13^C-NMR (101 MHz, DMSO-*d*_6_) δ 170.53 (C), 138.41 (C), 132.38 (CH), 131.65 (C), 131.57 (CH), 128.75 (CH), 125.31 (CH), 122.74 (C), 105.01 (CH). LC-MS (MeCN) R_t_ = 2.06 min [M + H]^+^ = 257.4. HRMS C_9_H_8_^79^BrN_2_S calc. 254.9592, found 254.9603, (Δ = 4.3 ppm, mDa 1.1).

*4-(3-Bromophenyl)thiazole* (**2ah**): Isolated in 51% using Hexane/EtOAc 1:1. ^1^H-NMR (400 MHz, CDCl_3_) δ 8.91 (d, *J* = 2.0 Hz, 1H), 8.13 (t, *J* = 1.8 Hz, 1H), 7.88 (ddd, *J* = 8.0, 1.7, 1.0 Hz, 1H), 7.59 (d, *J* = 2.0 Hz, 1H), 7.50 (ddd, *J* = 8.0, 2.0, 1.1 Hz, 1H), 7.35–7.30 (m, 1H).LC-MS Rt 2.64 found 240.1; HRMS found 239.9501 for C_9_H_7_NS^79^Br calc. 239.9483 (7.5 ppm, mDa 1.8).

*4-(4-Trifluorophenyl)thiazol-2-amine* (**1ai**): Prepared following the procedure of Potopnyk et al. [34]/ Recrystallised from acetone to yield a white solid, 78% yield ^1^H-NMR (400 MHz, DMSO-*d*_6_) δ 7.99 (m, 2H), 7.82 (m, 2H), 7.46 (s, 1H). ^13^C-NMR (101 MHz, DMSO-*d*_6_) δ 170.61 (C), 139.09 (C), 133.46 (C), 129.46 (C, q, *J* = 32.3 Hz), 126.95 (CH), 126.33 (CH, q, *J* = 3.8 Hz), 124.48 (C, q, *J* = 272.4 Hz), 106.10 (CH). ^19^F NMR (376 MHz, DMSO) δ −61.26. 

*4-(4-Trifluorophenyl)thiazole* (**2ai**): Isolated in 43% using Hexane/EtOAc 1:1. ^1^H-NMR (400 MHz, CDCl_3_) δ 8.94 (d, *J* = 2.0 Hz, 1H), 8.07 (app. dp, *J* = 7.7, 0.9 Hz, 2H), 7.77–7.62 (m, 3H). ^13^C-NMR (101 MHz, CDCl_3_) δ 154.90 (C), 153.33 (CH), 137.38 (C), 130.08 (C, q, *J* = 31.8 Hz), 128.80 (C), 126.65 (CH), 125.83 (CH, q, *J* = 3.8 Hz), 124.13 (C, q, *J* = 273 Hz), 114.44 (CH). LC-MS Rt 2.71 found 230.1 [M + H] 271.1 [MH + MeCN], HRMS found 230.0253 for C_10_H_7_NSF_3_ calc. 230.0251 (0.9 ppm).

*5-Amino-1-(2,6-difluorobenzyl)-1H-1,2,3-triazole-4-carbonitrile* (**1aj**): Prepared following the procedure of Brand et al. [35] in 77% yield following recrystallization from ethanol as a red solid. ^1^H-NMR (400 MHz, DMSO-*d*_6_) δ 7.51 (m, 1H), 7.26 (s, 2H), 7.21–7.10 (m, 2H), 5.40 (d, *J* = 1.1 Hz, 2H). ^13^C-NMR (101 MHz, DMSO-*d*_6_) δ 161.17 (C, dd, *J* = 249.3, 8.2 Hz), 148.59 (C), 132.04 (CH, t, *J* = 10.7 Hz), 114.02 (C), 112.31 (CH, app. dd, *J* = 24.9, 5.8 Hz), 101.34 (C), 37.89 (CH_2_). ^19^F NMR (376 MHz, DMSO-*d*_6_) δ −114.54. ASAP-MS (MeCN) R_t_ = 0.44 min [M + H]^+^ = 236.1. HRMS C_10_H_8_N_5_F_2_ calc. 236.0748, found 236.0755, (Δ = 3.0 ppm).

*1-(2,6-Difluorobenzyl)-1H-1,2,3-triazole-4-carbonitrile* (**2aj**): [36] Recrystallised from ethanol and water as a red solid in 73% yield. ^1^H-NMR (400 MHz, DMSO-*d*_6_) δ 9.18 (s, 1H), 7.54 (tt, *J* = 8.5, 6.7 Hz, 1H), 7.33–7.07 (m, 2H), 6.02 – 5.52 (m, 2H). ^13^C-NMR (101 MHz, DMSO-*d*_6_) δ 162.1.2 (C, dd, *J* = 249.5, 6.7 Hz), 132.94, (CH), 132.62 (CH, t, *J* = 10.6 Hz), 120.12 (C), 112.51 (C), 112.46 (CH, m), 110.80 (C, t, *J* = 19.1 Hz), 42.33 (CH_2_). ^19^F NMR (376 MHz, DMSO-*d*_6_) δ −114.42. LC-MS (MeCN) R_t_ = 1.93 min [M + H]^+^ = 221.1. HRMS C_10_H_7_N_4_F_2_ calc. 221.0639, found 221.0643, (Δ = 1.8 ppm).

*Methyl 5-amino-1-(phenylmethyl)-1H-1,2,3-triazole-4-carboxylate* (**1ak**): Eluent: hexane/ethyl acetate (25: 1). Prepared from the following procedure: benzyl bromide (20 mmol) and sodium azide (30 mmol) in acetone (5 mL) and water (2 mL) were stirred with sodium hydroxide (30 mmol) at ambient temperature for 24 h. The resulting mixture was concentrated under reduced pressure and then ethyl acetate (10 mL) and water (10 mL) were added. Following phase separation, the organic phase was concentrated under reduce pressure to give benzyl azide. The benzyl azide and cyanoacetic acid (20 mmol) was dissolved in dimethyl formamide (5 mL) and water (2 mL) and heated at 120 °C for 48 h. To the resultant mixture was added ethyl acetate (10 mL) and water (10 mL). Following phase-separation, the organic phase was concentrated under reduced pressure, to give a brown oil, which was twice purified by column chromatography to give the desired product as a pale powder in 8% yield. ^1^H-NMR (400 MHz, CDCl_3_) δ 7.49 (m, 2H), 7.16 (m, 3H), 4.56 (s, 2H), 3.84 (s, 3H). LC-MS (MeOH) R_t_ = 0.56 min [M + H]^+^ = 232.2.

*Ethyl oxazole-4-carboxylate* (**2am**): ^1^H-NMR (400 MHz, CDCl_3_) δ 8.27 (d, *J* = 1.1 Hz, 1H), 7.94 (d, *J* = 1.1 Hz, 1H), 4.39 (q, *J* = 7.1 Hz, 2H), 1.38 (t, *J* = 7.1 Hz, 3H). ^13^C-NMR (101 MHz, CDCl_3_) δ 160.95 (C), 151.40 (CH), 144.01 (CH), 133.33 (C), 61.31 (CH_2_), 14.24 (CH_3_). EI GC polar compounds (MeCN) R_t_ = 3.20 min M^+^ = 141.1. HRMS C_6_H_8_NO_3_ calc. 142.050419, found 142.050416 (Δ = 1.7 ppm).

*1-Chloro-4-phenoxybenzene* (**2al**): ^1^H-NMR (400 MHz, CDCl_3_) δ 7.46–7.35 (m, 1H), 7.27–7.17 (m, 1H), 7.10–6.98 (m, 1H). ^13^C-NMR (101 MHz, CDCl_3_) δ 155.04, 146.68, 130.32, 128.47, 126.89, 125.14, 119.40, 118.17. LC-MS (MeOH) R_t_ = 1.21 min [M + H]^+^ = 205.0.

*6-methyl-[1,2,4]triazolo[1,5-a]pyridin-2-amine* (**1an)** was prepared according to the general procedure of Verček et al. [37]. *N*-Ethoxycarbonyl-*N*’-(5-methyl-2-pyridinyl)thiourea: ^1^H-NMR (400 MHz, DMSO-*d_6_*) δ 12.11 (s, 1H), 11.43 (s, 1H), 8.57 (s, 1H), 8.32–8.02 (m, 1H), 7.69 (ddd, *J* = 8.5, 2.4, 0.8 Hz, 1H), 4.22 (q, *J* = 7.1 Hz, 2H), 2.28 (s, 3H), 1.26 (t, *J* = 7.1 Hz, 3H). ^13^C-NMR (101 MHz, DMSO-*d_6_*) δ 177.57 (C), 153.97, 149.57, 148.54, 138.76, 130.85, 115.48, 62.63 (CH_2_), 17.81 (CH_3_), 14.57 (CH_3_). ESI-LC MeCN (TQD) R_t_ = 2.20 min, M^+^ = 241.3 HRMS C_10_H_14_N_3_O_2_S calc. 240.0807, found 240.0807 (Δ = 2.9 ppm). **6-**Methyl-[1,2,4]triazolo[1,5-a]pyridin-2-amine (**1an**): ^1^H-NMR (400 MHz, DMSO-*d_6_*) δ 8.37 (s, 1H), 7.25 (d, *J* = 1.3 Hz, 2H), 5.90 (s, 2H), 2.25 (d, *J* = 1.1 Hz, 3H). ^13^C-NMR (101 MHz, DMSO-*d_6_*) δ 166.32 (C), 149.46 (C), 131.57 (CH), 126.16 (CH), 121.08 (C), 112.25 (CH), 17.60 (CH_3_). ESI-LC MeCN (TQD) R_t_ = 0.70 min, M^+^ = 149.6. HRMS C_7_H_9_N_4_ calc. 149.0827, found 149.0830 (Δ = 2.0 ppm).

*2,6-Diethyl-5-methylpyrimidin-4-aminium acetate* (**1aq**): Starting material synthesized following the procedure Baxendale et al. [38]. Salt formed by addition of 1 equivalent of acetic acid and the material recrystallized from ethanol as white crystals in 60% yield. ^1^H-NMR (400 MHz, DMSO-*d*_6_) δ 13.37 (s, 1H), 8.79 (s, 1H), 8.19 (s, 1H), 2.72 (m, *J* = 7.6 Hz, 4H), 2.03 (s, 3H), 1.24 (t, *J* = 7.6 Hz, 3H), 1.16 (t, *J* = 7.6 Hz, 3H). ^13^C-NMR (101 MHz, DMSO-*d*_6_) δ 165.06 (C), 163.58 (C), 155.76 (C), 108.63 (C), 27.83 (CH_2_), 23.79 (CH_2_), 13.00 (CH_3_), 11.69 (CH_3_), 10.85 (CH_3_). ASAP-MS (MeCN) R_t_ = 0.34 min M^+^ = 166.1. HRMS C_9_H_16_N_3_ calc. 166.1344, found 166.1344, (Δ = 0.0 ppm).

## 5. Conclusions

In summary, we have developed a simple method for the replacement of heteroaromatic primary amino groups with hydrogen. Heteroaryl amines that are often challenging, undergo deamination in good yields in a single, simple reactor. A range of functional groups are tolerated under the optimised conditions, making use of the improved mixing and heat dissipation that working in flow has to offer. The method has allowed certain structures that are particularly challenging to be deaminated in a homogeneous organic system for the first time. The yields for the deamination of simple anilines are comparable to existing methods, but advantages in terms of reaction time and the ease of the process should be noted. This protocol would inspire further application of continuous flow towards improved yields, incorporating radical catalysts and in-line purifications. 

## Figures and Tables

**Table 1:** Conversion of methyl 2-aminothiophene-3-carboxylate (**1r**) to methyl thiophene-3-carboxylate (**2r**) in a range of different solvents.

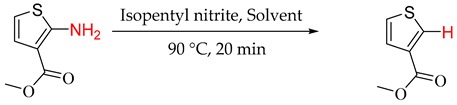	
Solvent	Conversion to Product (%)
THF	47
1,4-dioxane	34
1,2-dimethoxyethane	16
diethyl ether	24
cyclohexane	Not possible
polyethylene glycol	<3
butanol	<3
propan-2-ol	13
ethanol	<3
methanol	8
ethyl acetate	<3

All experiments were performed on: methyl 2-aminothiophene-3-carboxylate (1.0 mmol) in the stated solvent (10 mL), *iso*-pentyl nitrite (1.2 mmol) in the stated solvent (10 mL), both flowing at 0.25 mL min^−1^ through a 10.0 mL coil reactor maintained at 90 °C (residence time: 20 min). The conversions were calculated by integration of product ^1^H-NMR peaks relative to a quantified internal standard of nitrobenzene. THF—tetrahydrofuran.

**Table 2:** Conversion of methyl 2-aminothiazole-5-carboxylate (**1a**) to methyl thiazole-5-carboxylate (**2a**) at various temperatures in THF.

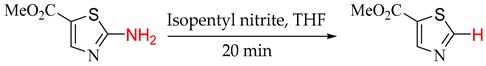	
Temperature / °C	Conversion to Product (%)
25	0
40	21
50	49
60	50
70	53
80	55
90	61
100	65
110	71
120	88

All experiments were: methyl 2-aminothiazole-5-carboxylate (1.0 mmol) in THF (10 mL), *iso*-pentyl nitrite (1.2 mmol) in THF (10 mL), both flowing at 0.25 mL min^−1^ through a 10.0 mL coil reactor at the stated temperature, (residence time: 20 min). The conversions were calculated by integration of product ^1^H-NMR peaks relative to a quantified internal standard of nitrobenzene. The reported value is the average of at least three repeat runs.

**Table 3:** Conversion of methyl 2-aminothiazole-5-carboxylate (**1a**) to methyl thiazole-5-carboxylate (**2a**) at various temperatures in 1,4-dioxane.

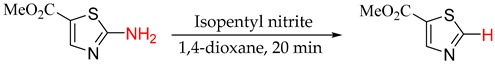	
Temperature/°C	Conversion to Product (%)
25	0
40	23
60	47
80	60
100	63
120	83
140	80
160	58

All experiments were: methyl 2-aminothiazole-5-carboxylate (1.0 mmol) in 1,4-dioxane (10 mL), *iso*-pentyl nitrite (1.2 mmol) in 1,4-dioxane (10 mL), both flowing at 0.25 mL min^−1^ through a 10.0 mL coil reactor at the stated temperature, (residence time: 20 min). The conversions were calculated by integration of product ^1^H-NMR peaks relative to a quantified internal standard of nitrobenzene. The reported value is the average of at least three repeat runs.

**Table 4:** Conversion of methyl 2-aminothiazole-5-carboxylate (**1a**) to methyl thiazole-5-carboxylate (**2a**) at various temperatures and reactor coil sizes in THF.

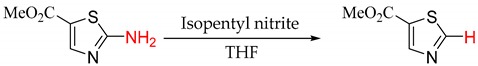			
Coil Volume/mL	Residence Time/min	Temperature/°C	Conversion (%)
5		60	41
	10	90	55
		120	73
		60	50
10	20	90	61
		120	88
		60	59
20	40	90	76
		120	87

All experiments were: methyl 2-aminothiazole-5-carboxylate (1.0 mmol) in THF (10 mL), *iso*-pentyl nitrite (1.2 mmol) in THF (10 mL), both flowing at 0.25 mL min^−1^ through the stated coil reactor (1 mm i.d.) at the stated temperature. The conversions were calculated by integration of product ^1^H-NMR peaks relative to a quantified internal standard of nitrobenzene.

**Table 5:** Conversion of 4-(5-amino-4-cyano-1*H*-pyrazol-1-yl)-benzoic acid (**1u**) to 4-(4-cyano-1*H*-pyrazol-1-yl)-benzoic acid (**2u**) with various quantities of gallic and salicylic acid.

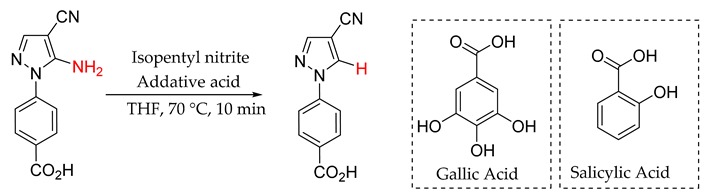		
Catalyst Loading (mol%)	Conversion (%) Gallic Acid	Conversion (%) Salicylic Acid
0	0	0
1	0	0
5	33	1
10	26	26
10^a^	18	22
20	22	30
100	0	21

All experiments were: 4-(5-amino-4-cyano-1H-pyrazol-1-yl)-benzoic acid (1.0 mmol) and the stated quantity of gallic acid or salicylic acid in THF and *iso*-pentyl nitrite (1.2 mmol) in THF (50 mL), flow rate 1.0 mL min^−1^, through a 10 mL coil reactor maintained at 70 °C, giving a residence time of 10 min. The conversions were calculated by integration of product ^1^H-NMR peaks relative to a quantified internal standard of nitrobenzene. ^a^Reaction performed at 120 °C.

**Table 6:** Flow hydrodediazoniation of aromatic amines.

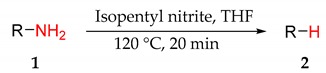
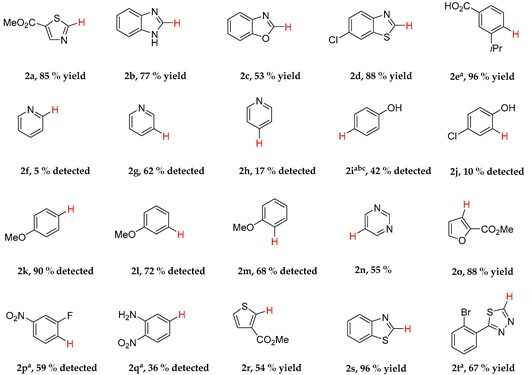
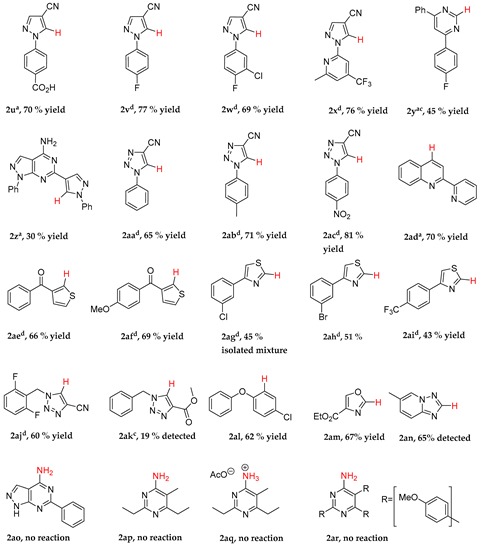

Starting material (1.0 mmol) in THF (10 mL), *iso*-pentyl nitrite (1.2 mmol) in THF (10 mL), 10 mL coil volume, 20 min residence time. ^a^ 20 mL of THF was used for both reagent solutions. ^b^ DMF (2 mL) was added to the starting material solution to improve solubility. ^c^ Concentrations of 0.5 mmol of starting material and 0.6 mmol of isopentyl nitrite. ^d^ Concentrations of 10.0 mmol of starting material and 12.0 mmol of isopentyl nitrite. **Yield** refers to pure, isolated and characterised yield. **Detected** refers to the conversion of starting material to product as quantified by integration of product ^1^H-NMR peaks relative to a quantified internal standard of nitrobenzene. **No reaction** refers to compounds for which there was no evidence of formation and only starting material was recovered.

## Supplementary Materials

The following are available online: NMR spectra of products where an isolated yield is reported and for starting materials that were not commercially available, hence we have synthesised ourselves.
