# Supplementary material for: Flow Hydrodediazoniation of Aromatic Heterocycles
Source: Molecules. 2019 May 24;24(10):1996. doi: 10.3390/molecules24101996 (PMC6572451; doi:10.3390/molecules24101996)
Supplement: Supplementary file 1 [file molecules-24-01996-s001.pdf]

Supplementary Information

Flow Hydrodediazoniation of Aromatic Heterocycles

Liesa Röder,[1] Alexander J. Nicholls[2] and Ian R. Baxendale[2,*]

[1] Department of Biology, Chemistry, and Pharmacy, Freie Universität Berlin; 14195 Berlin, Germany.
[2] Department of Chemistry, University of Durham, South Road, Durham, Durham, UK. DH1 3LE.
[3] Thomas Swan & Co. Ltd. Rotary Way, Consett, Durham, UK. DH8 7ND.
* Correspondence: i.r.baxendale@durham.ac.uk;

Copies of ^{1}H, ^{13}C, DEPT 135 and if applicable ^{19}F spectra for staring materials **1** and isolated products **2**.

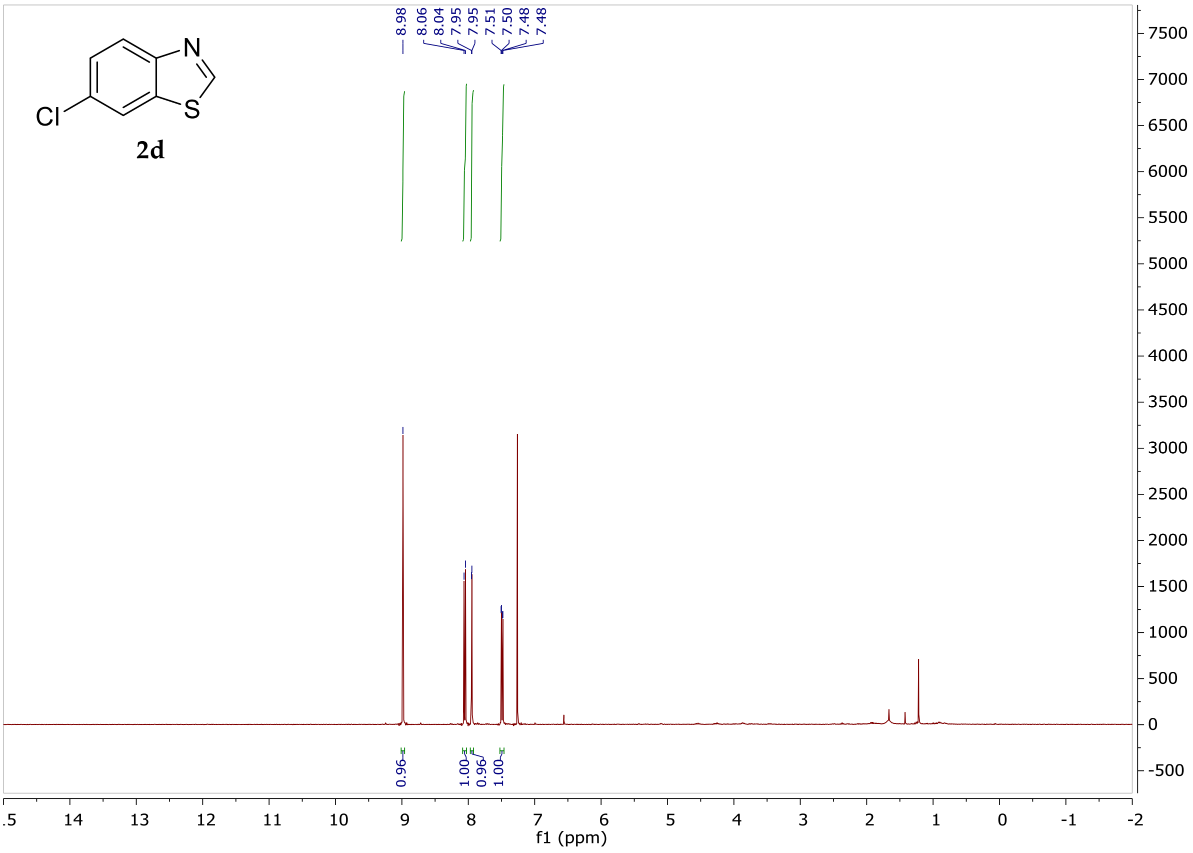

N
S
Cl
2d
8.98
8.06
8.04
7.95
7.95
7.51
7.50
7.48
7.48
0.96
1.00
0.96
1.00
f1 (ppm)

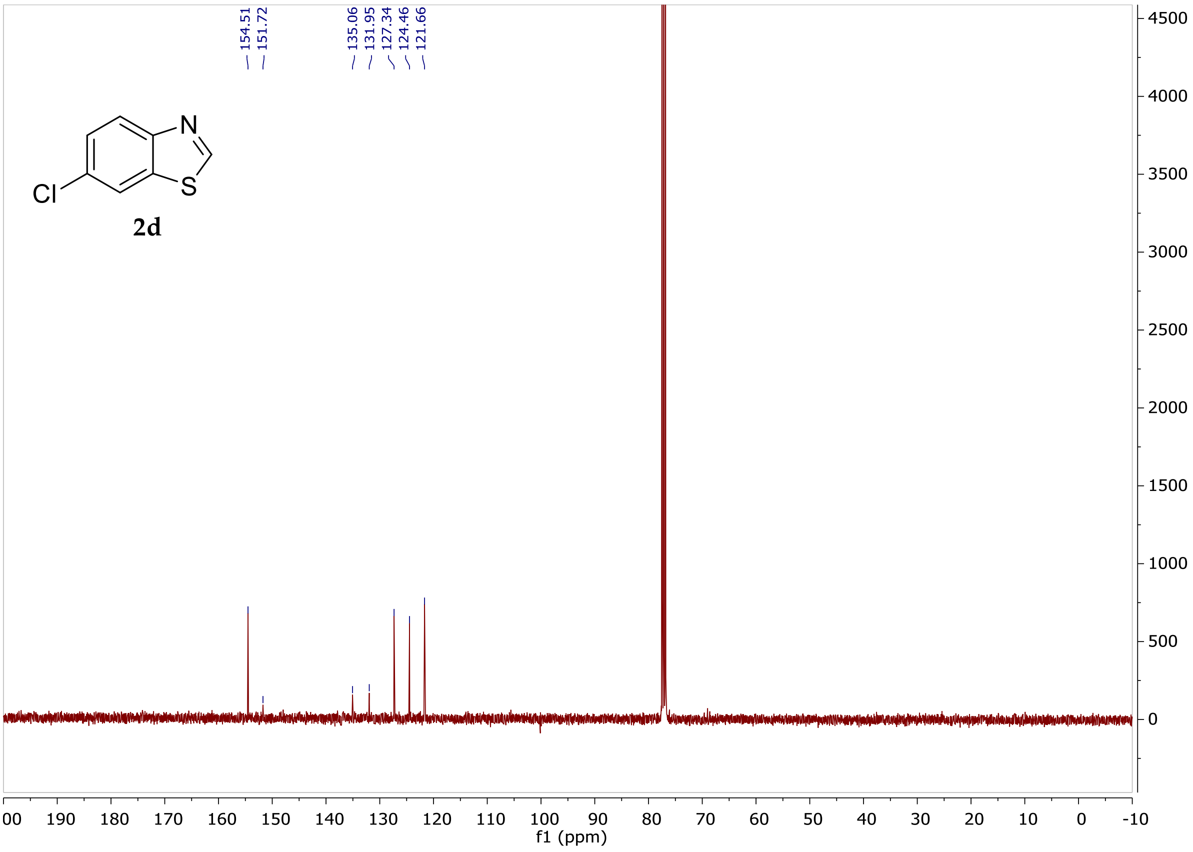

154.51
151.72
135.06
131.95
127.34
124.46
121.66
N
S
Cl
2d
f1 (ppm)

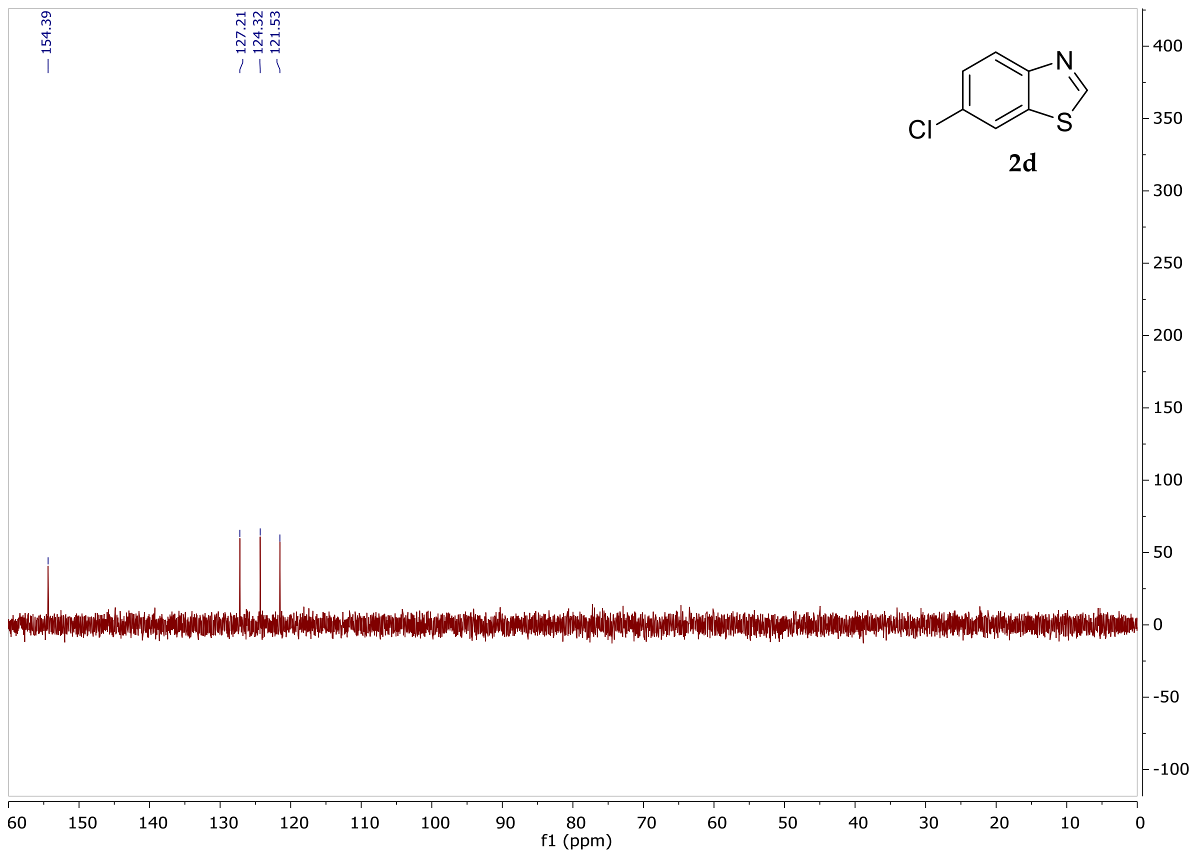
154.39
127.21
124.32
121.53
Cl
N
S
2d
f1 (ppm)

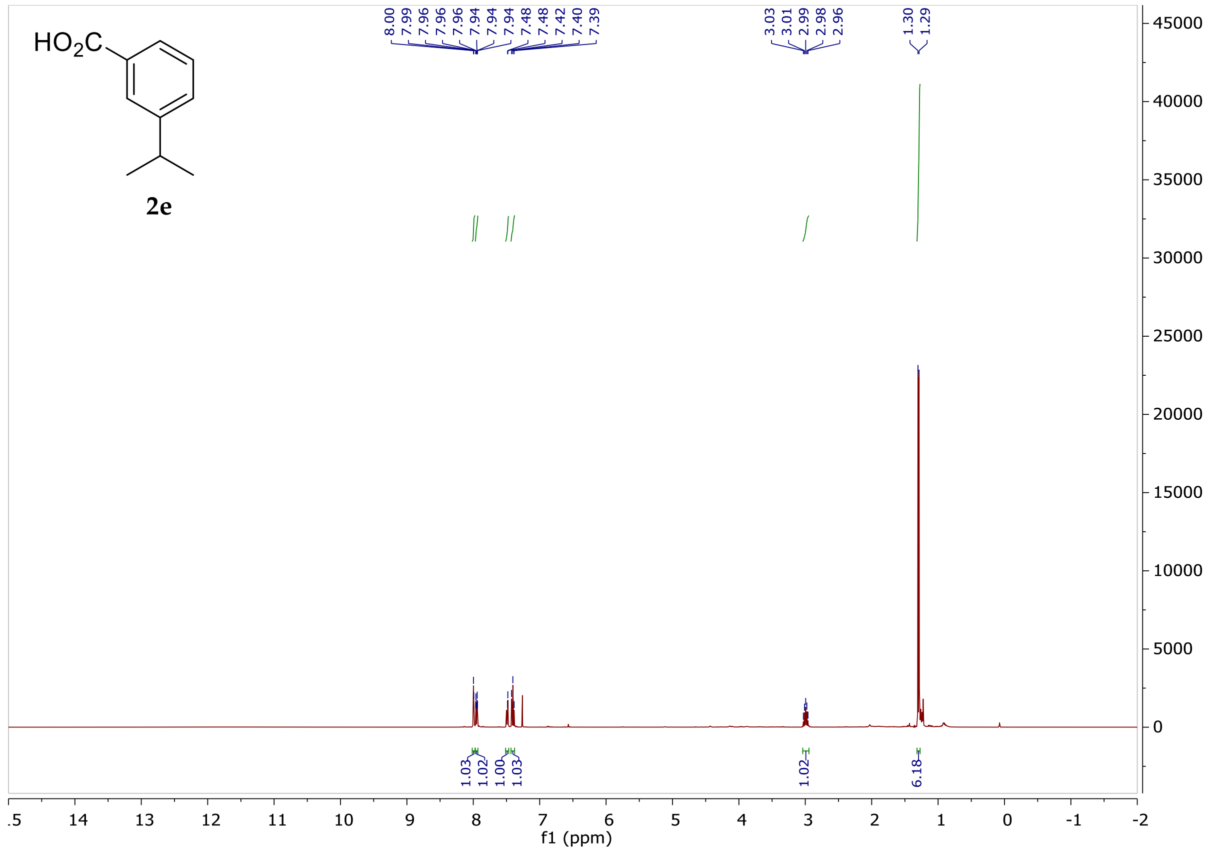

HO2C
2e
8.00
7.99
7.96
7.96
7.96
7.94
7.94
7.94
7.48
7.48
7.42
7.40
7.39
3.03
3.01
2.99
2.98
2.96
1.30
1.29
1.03
1.02
1.00
1.03
1.02
6.18
f1 (ppm)

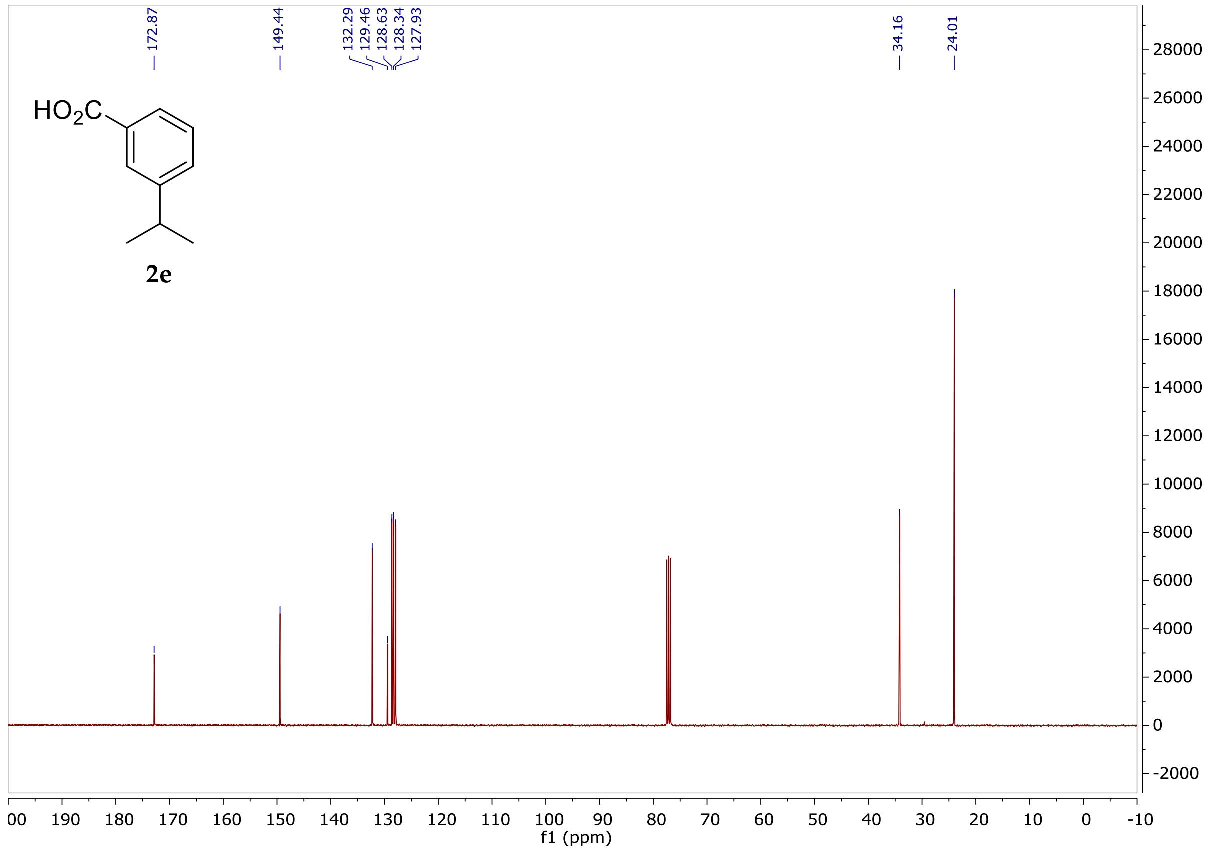

HO2C
2e
172.87
149.44
132.29
129.46
128.63
128.34
127.93
34.16
24.01
f1 (ppm)

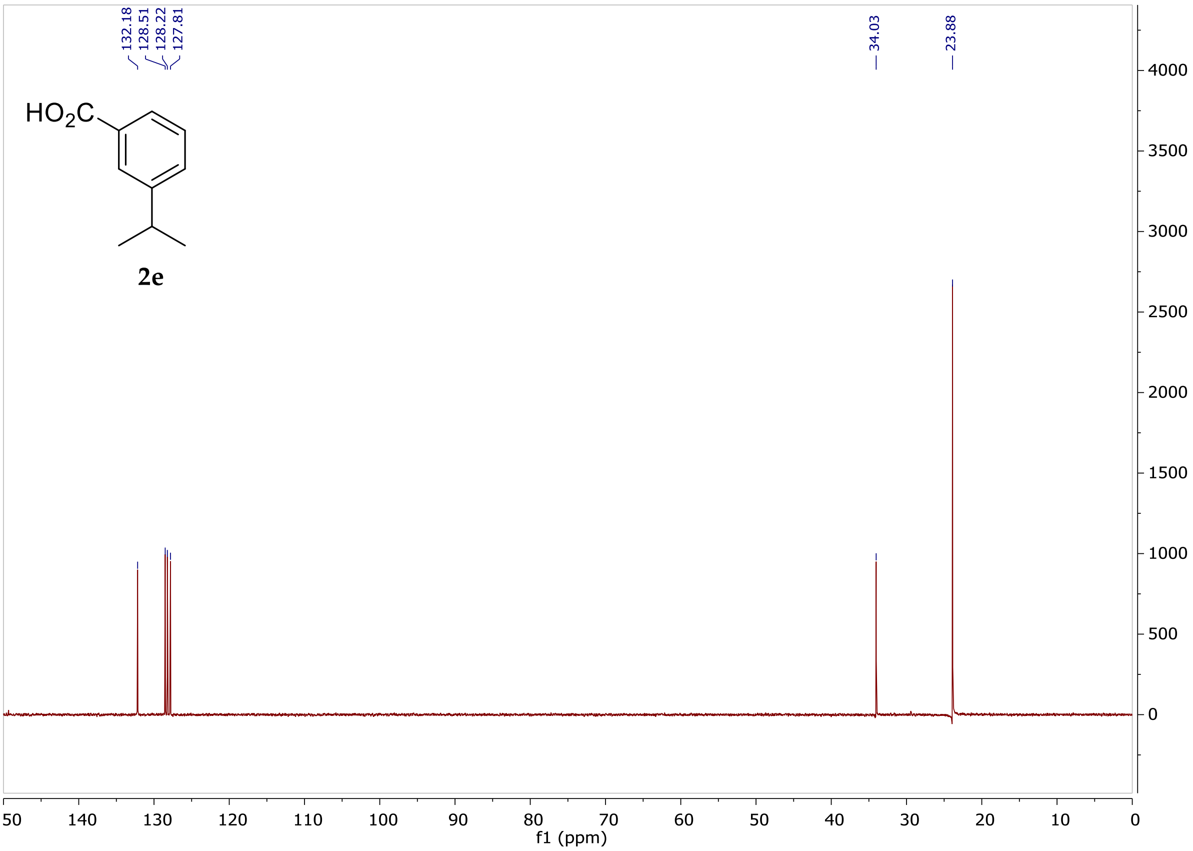
132.18
128.51
128.22
127.81
34.03
23.88
HO2C
2e
4000
3500
3000
2500
2000
1500
1000
500
0
50
140
130
120
110
100
90
80
70
60
50
40
30
20
10
0
f1 (ppm)

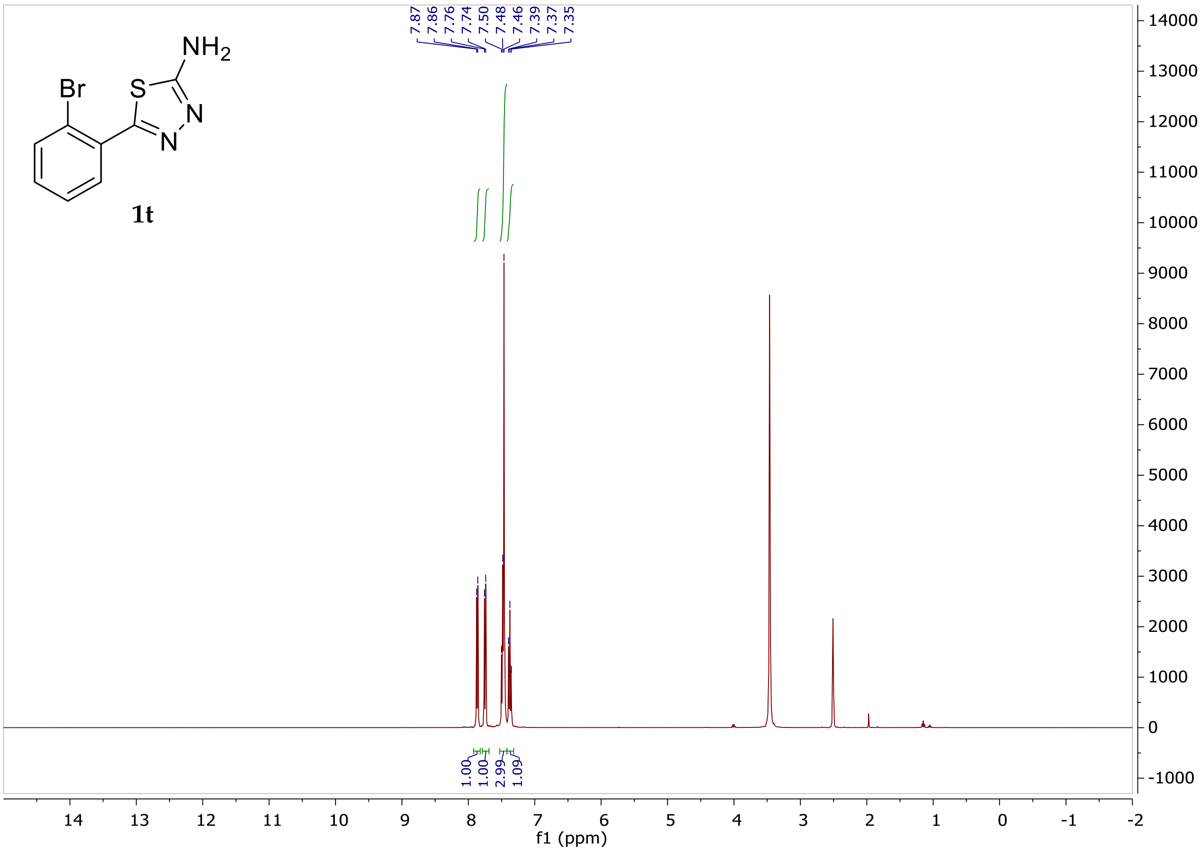

7.87
7.86
7.76
7.74
7.50
7.48
7.46
7.39
7.37
7.35
NH2
Br
S
N
N
1t
1.00
1.00
2.99
1.09
f1 (ppm)

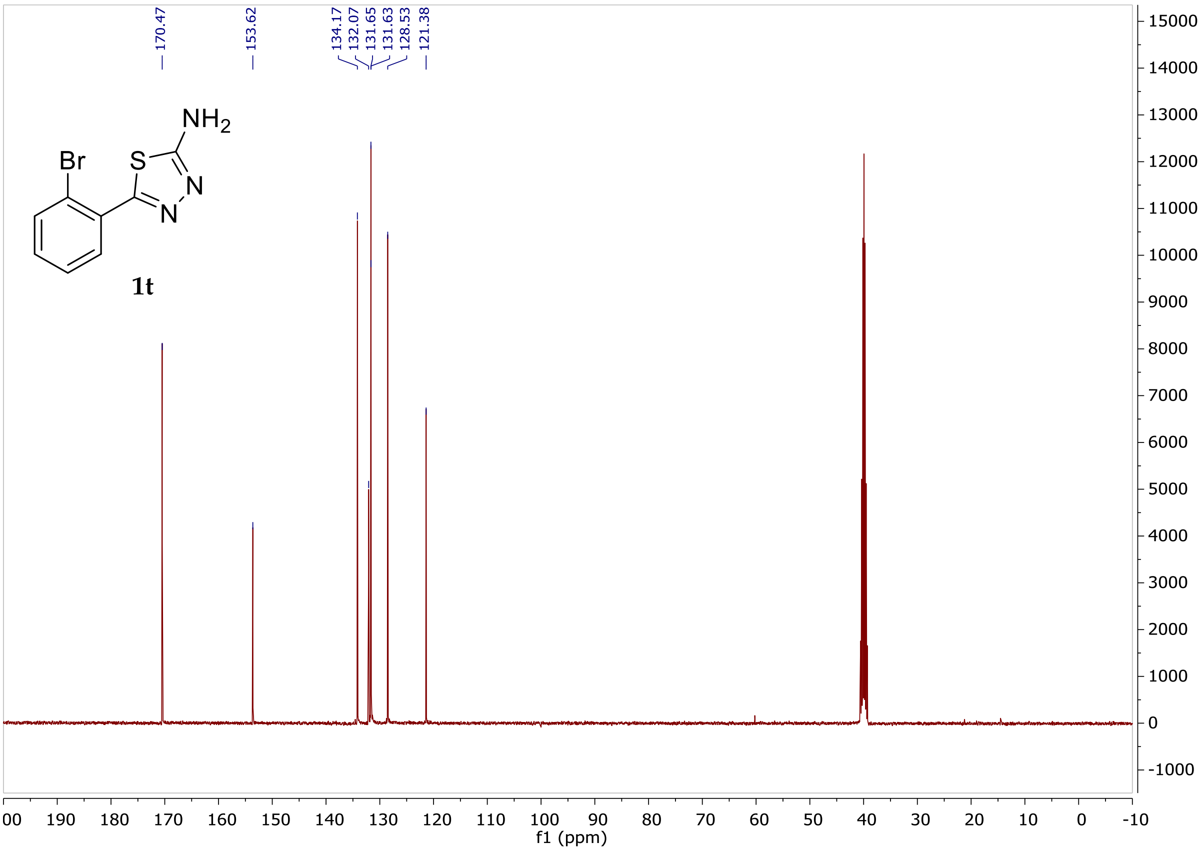

170.47
153.62
134.17
132.07
131.65
131.63
128.53
121.38
NH2
Br
S
N
N
1t
f1 (ppm)

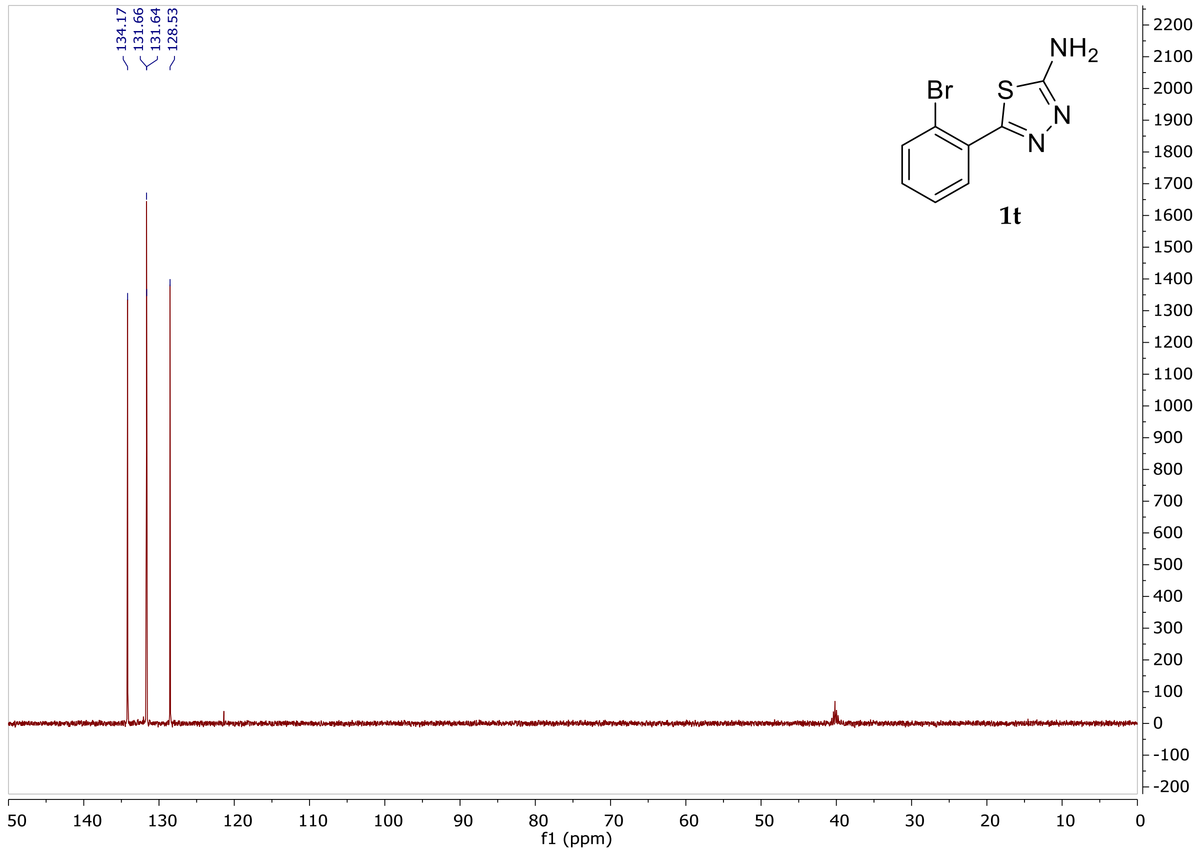
134.17
131.66
131.64
128.53
Br
S
N
N
NH2
1t
f1 (ppm)

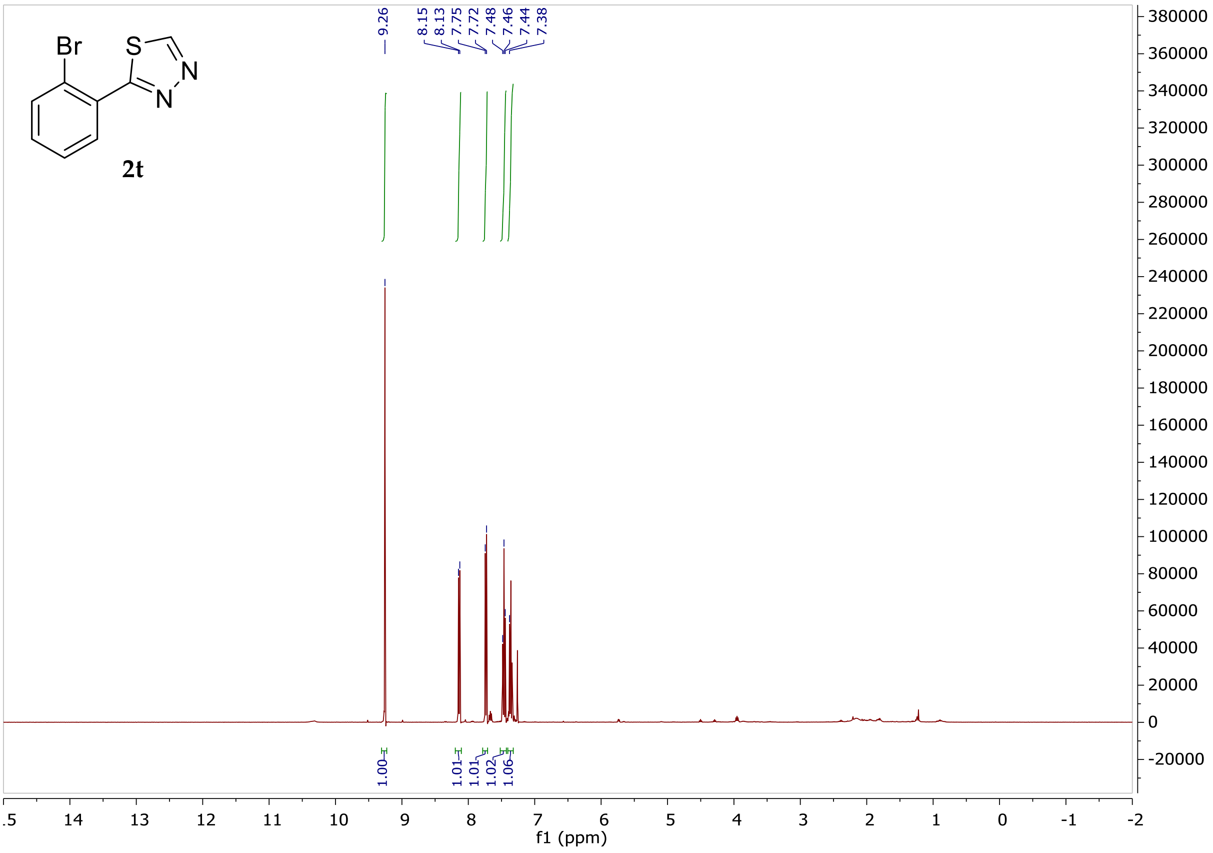

9.26
8.15
8.13
7.75
7.72
7.48
7.46
7.44
7.38
Br
S
N
N
2t
1.00
1.01
1.01
1.02
1.06
f1 (ppm)

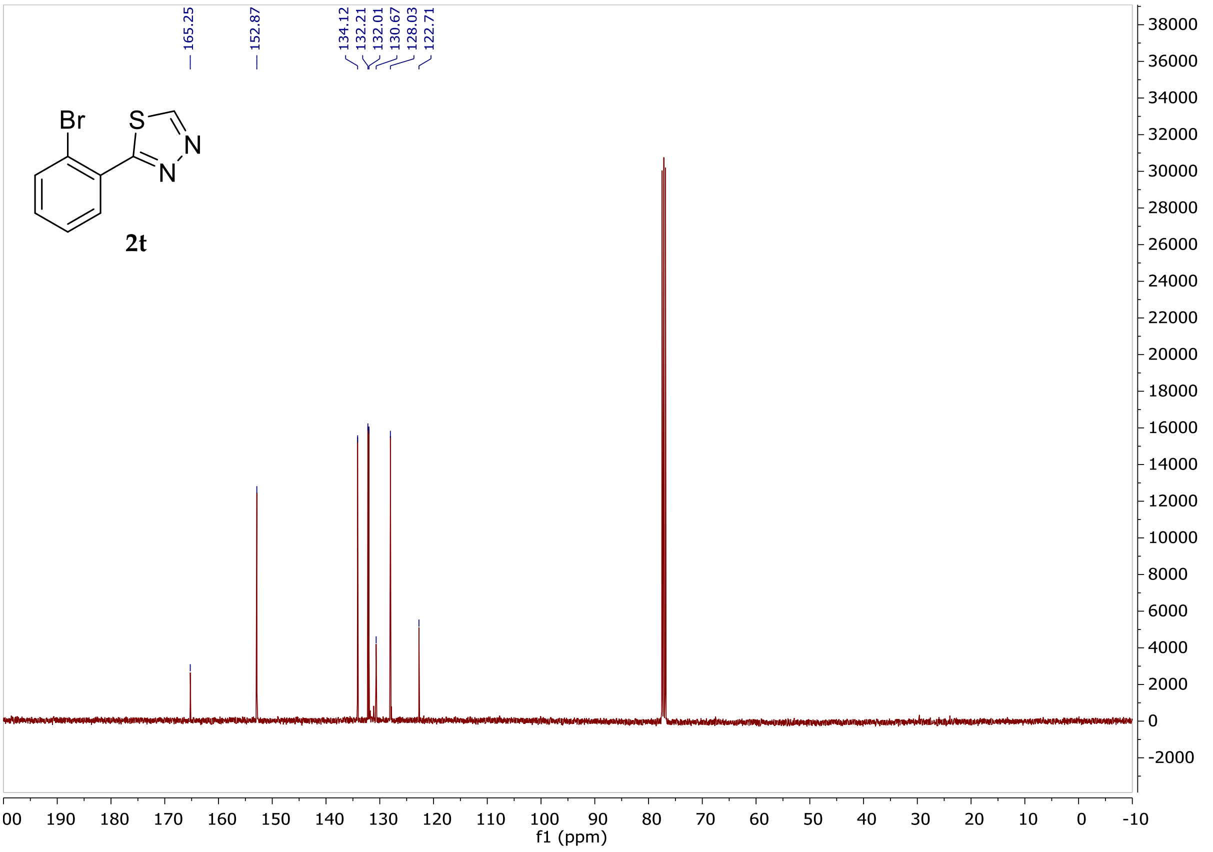

165.25
152.87
134.12
132.21
132.01
130.67
128.03
122.71
Br
S
N
N
2t
f1 (ppm)

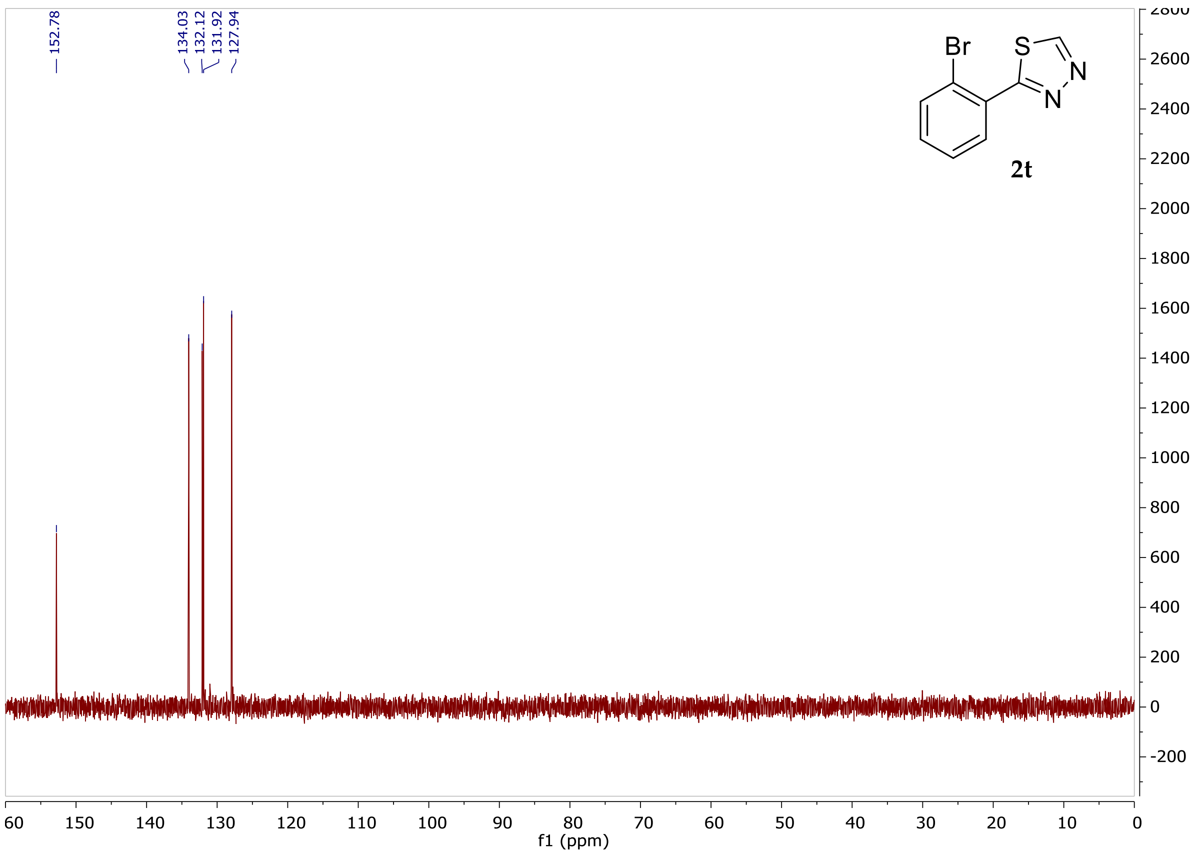
152.78
134.03
132.12
131.92
127.94
Br
S
N
N
2t
f1 (ppm)

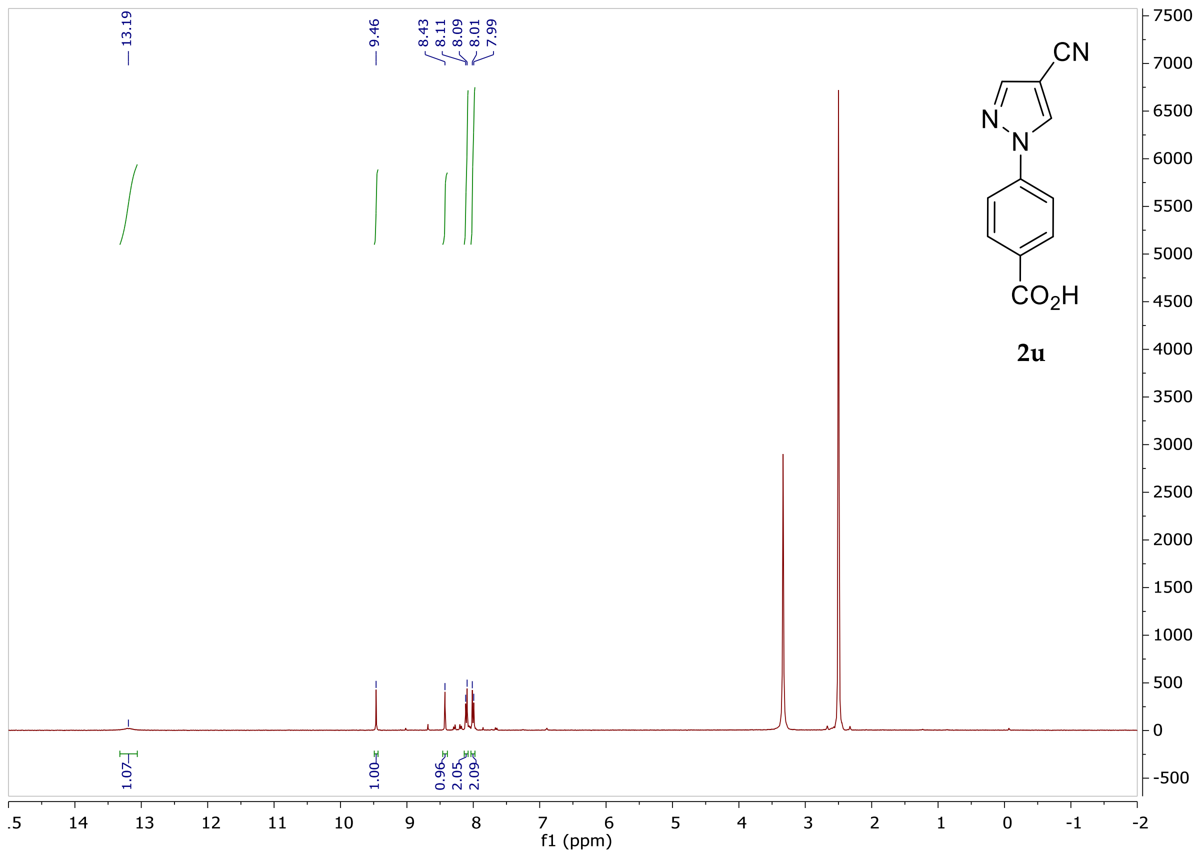
13.19
9.46
8.43
8.11
8.09
8.01
7.99
CN
N
N
CO2H
2u
1.07
1.00
0.96
2.05
2.09
f1 (ppm)

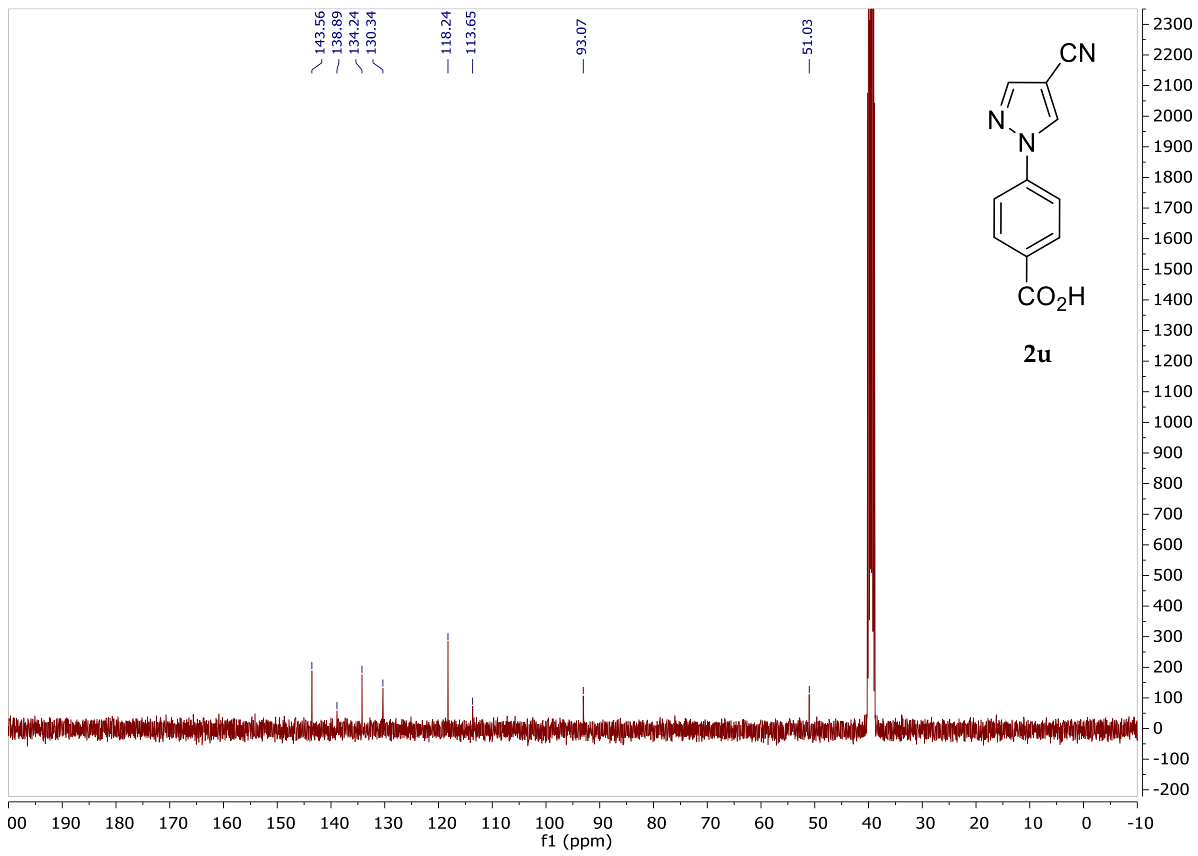
143.56
138.89
134.24
130.34
118.24
113.65
93.07
51.03
CN
N
N
CO2H
2u
f1 (ppm)

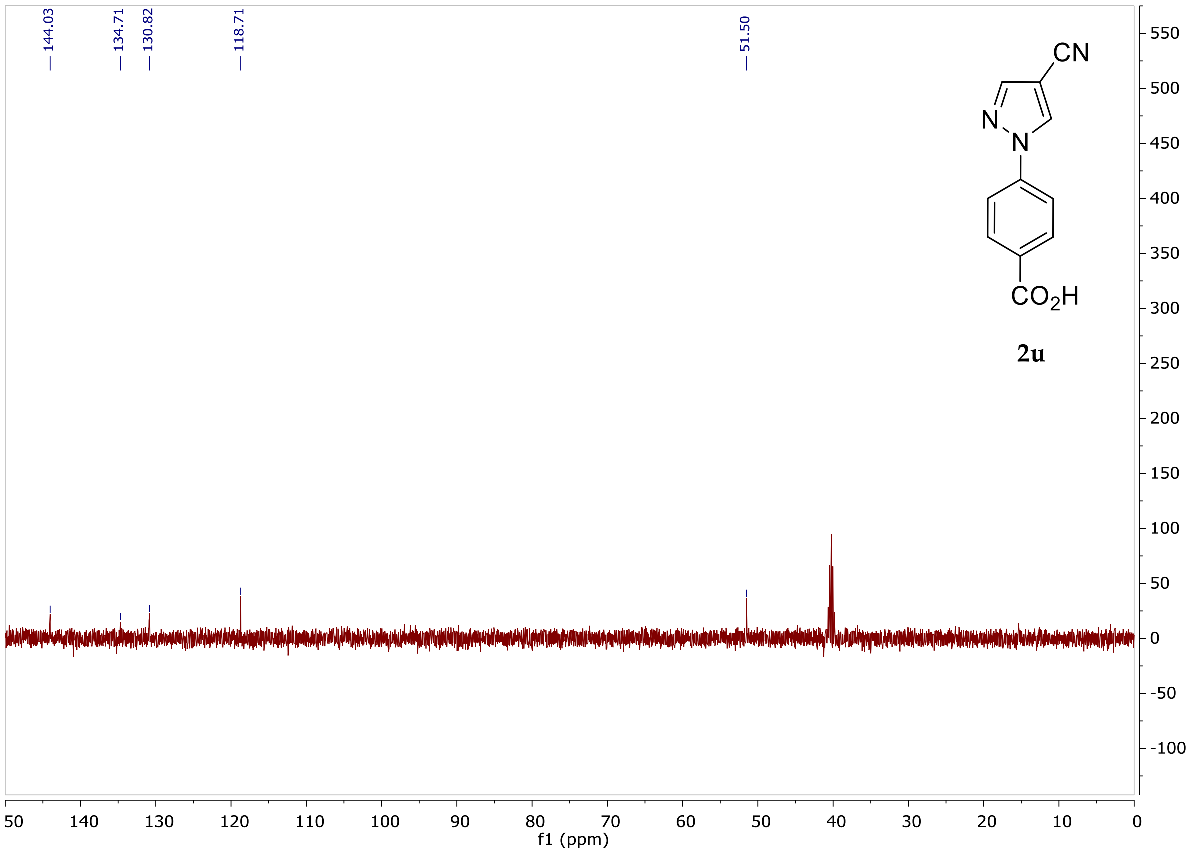
144.03
134.71
130.82
118.71
51.50
CN
N
N
CO2H
2u
f1 (ppm)

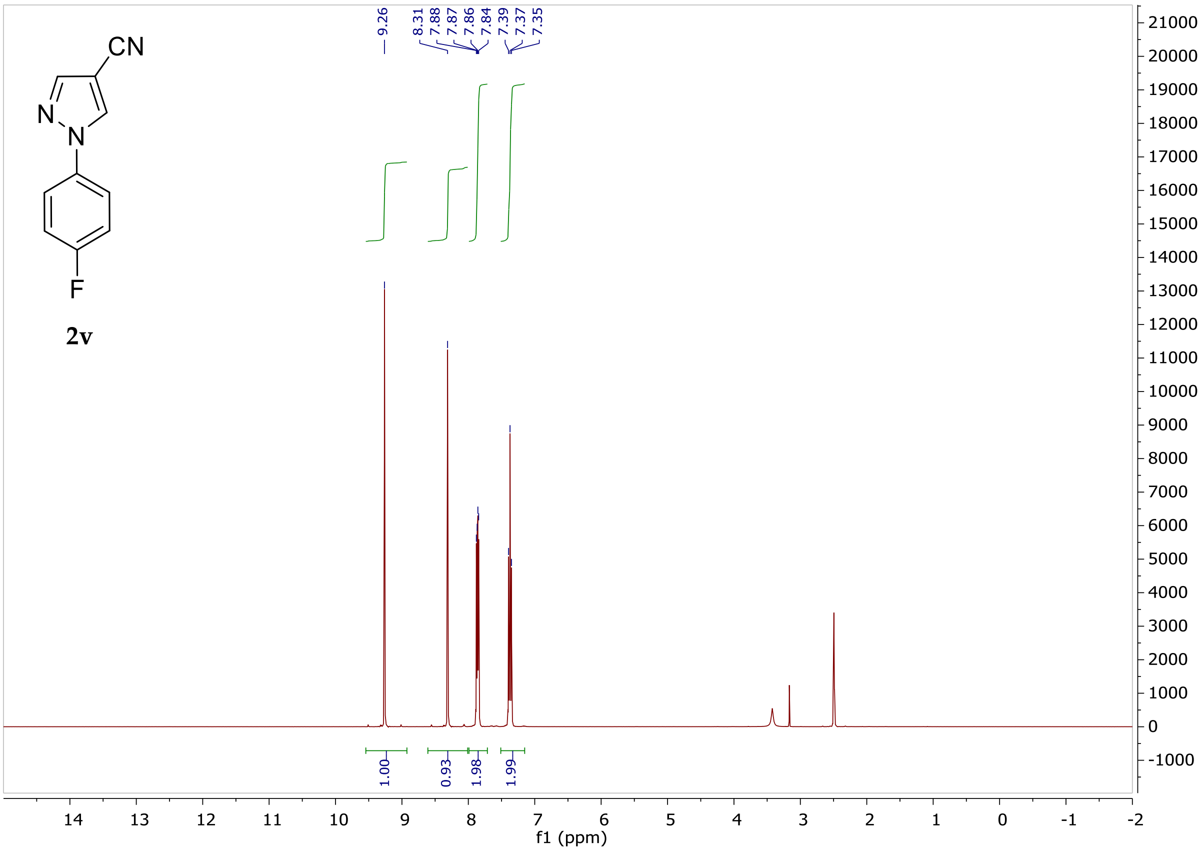

CN
N
N
F
2v
9.26
8.31
7.88
7.87
7.86
7.84
7.39
7.37
7.35
1.00
0.93
1.98
1.99
f1 (ppm)

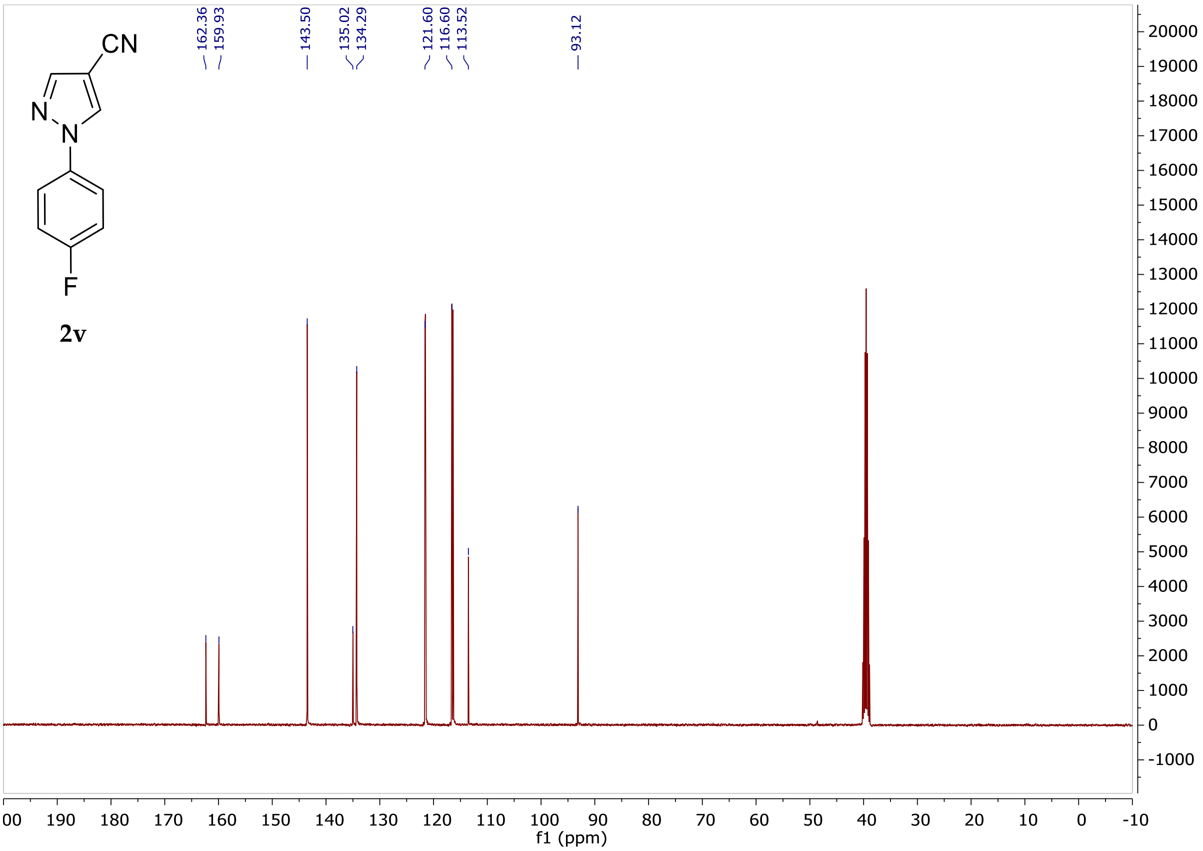

CN
N
N
F
2v
162.36
159.93
143.50
135.02
134.29
121.60
116.60
113.52
93.12
f1 (ppm)

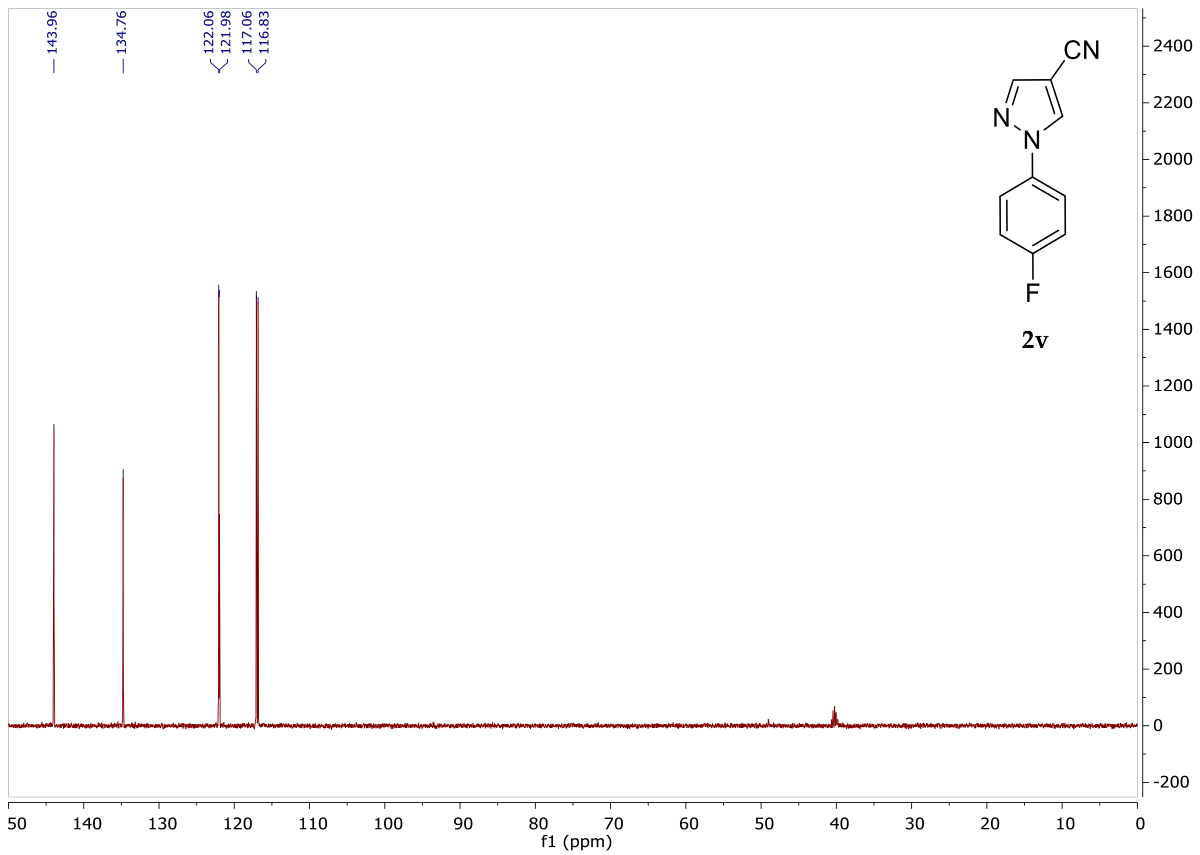
143.96
134.76
122.06
121.98
117.06
116.83
CN
N
N
F
2v
f1 (ppm)

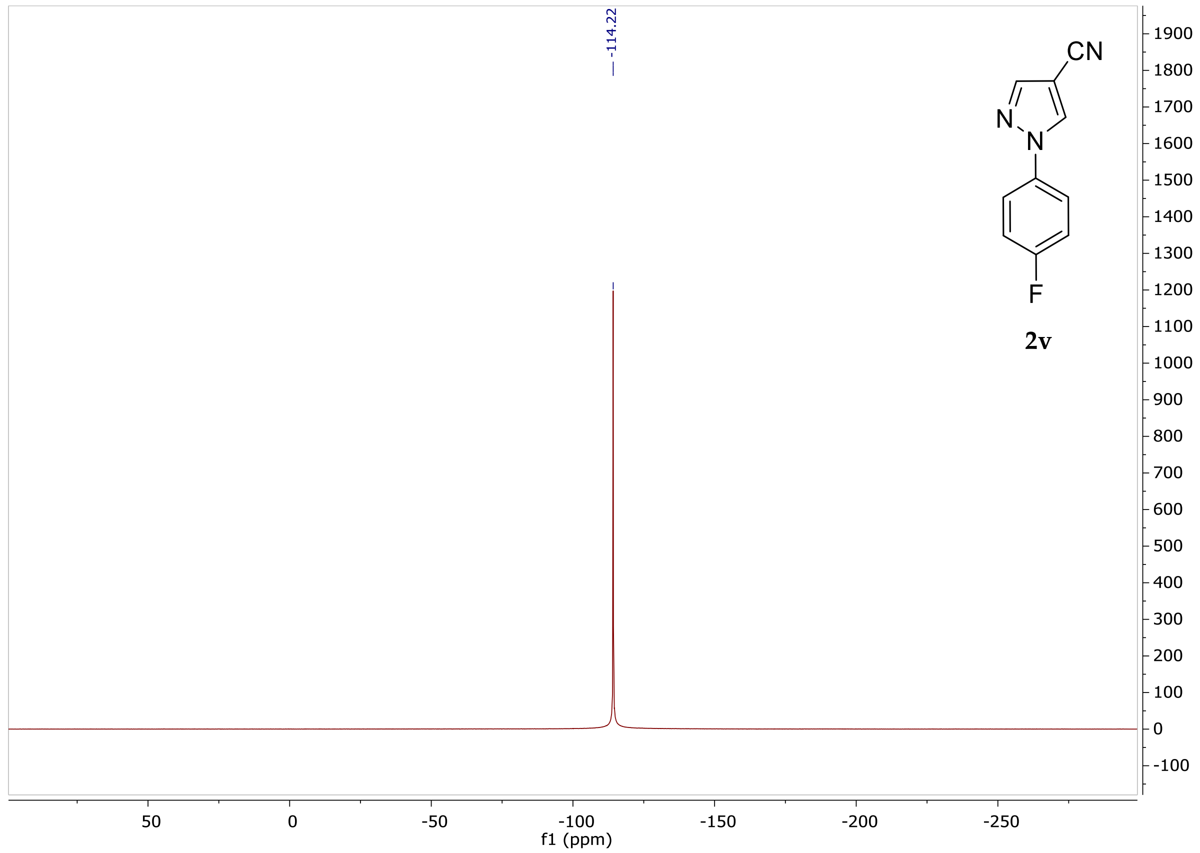
-114.22
CN
N
N
F
2v
f1 (ppm)

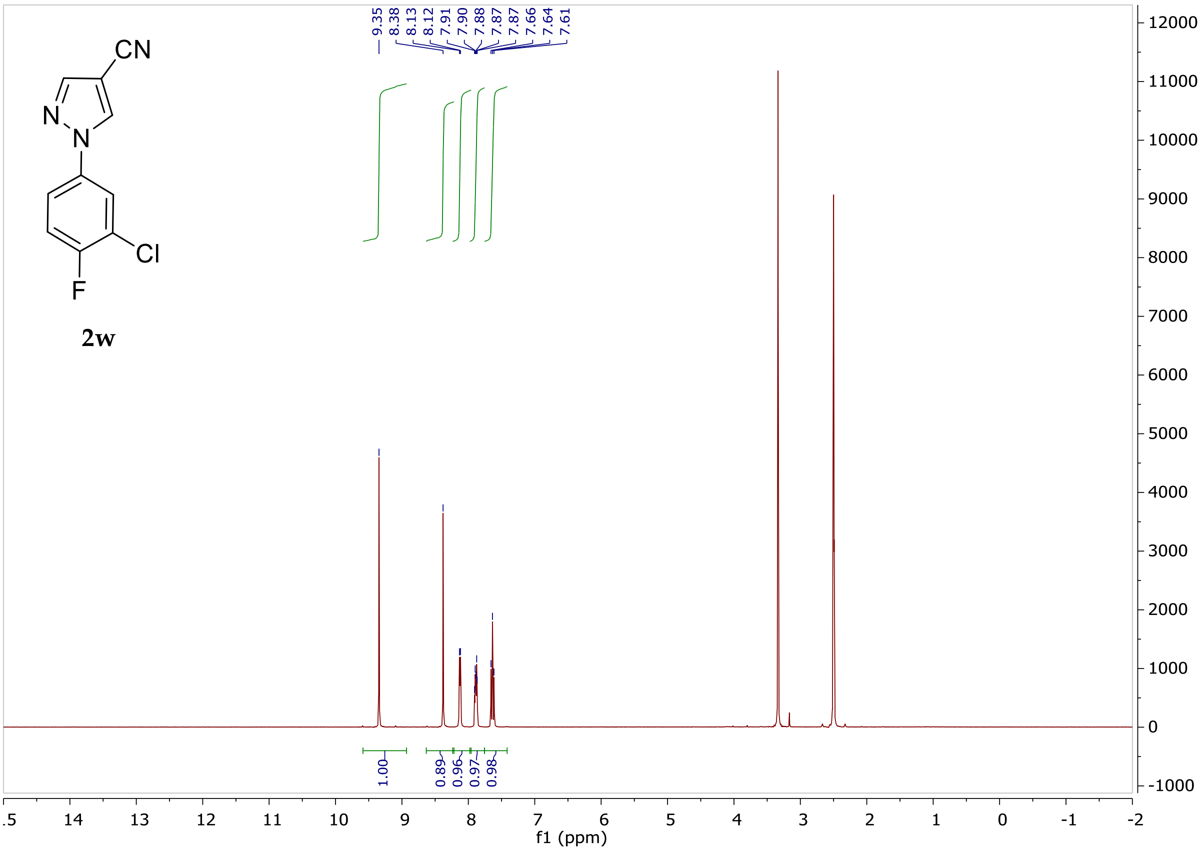

CN
N
N
Cl
F
2w

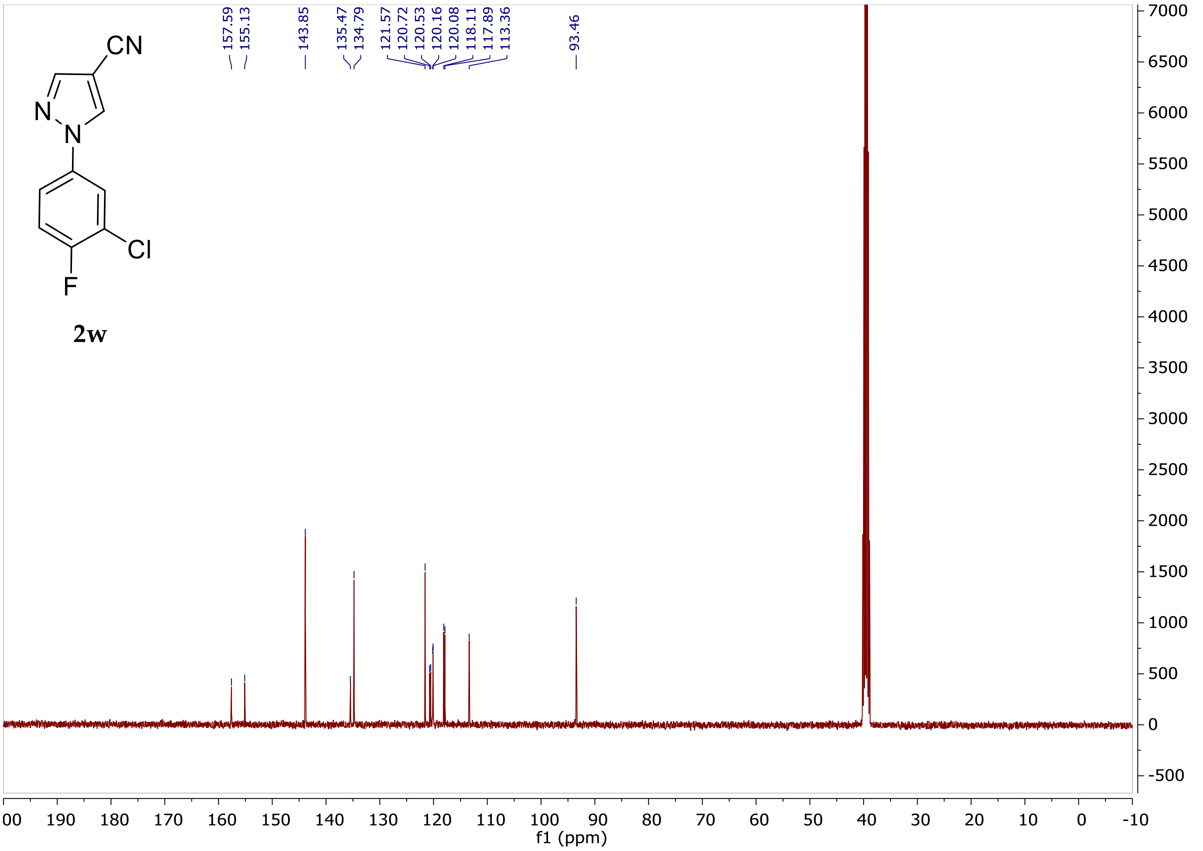

CN
N
N
Cl
F
2w

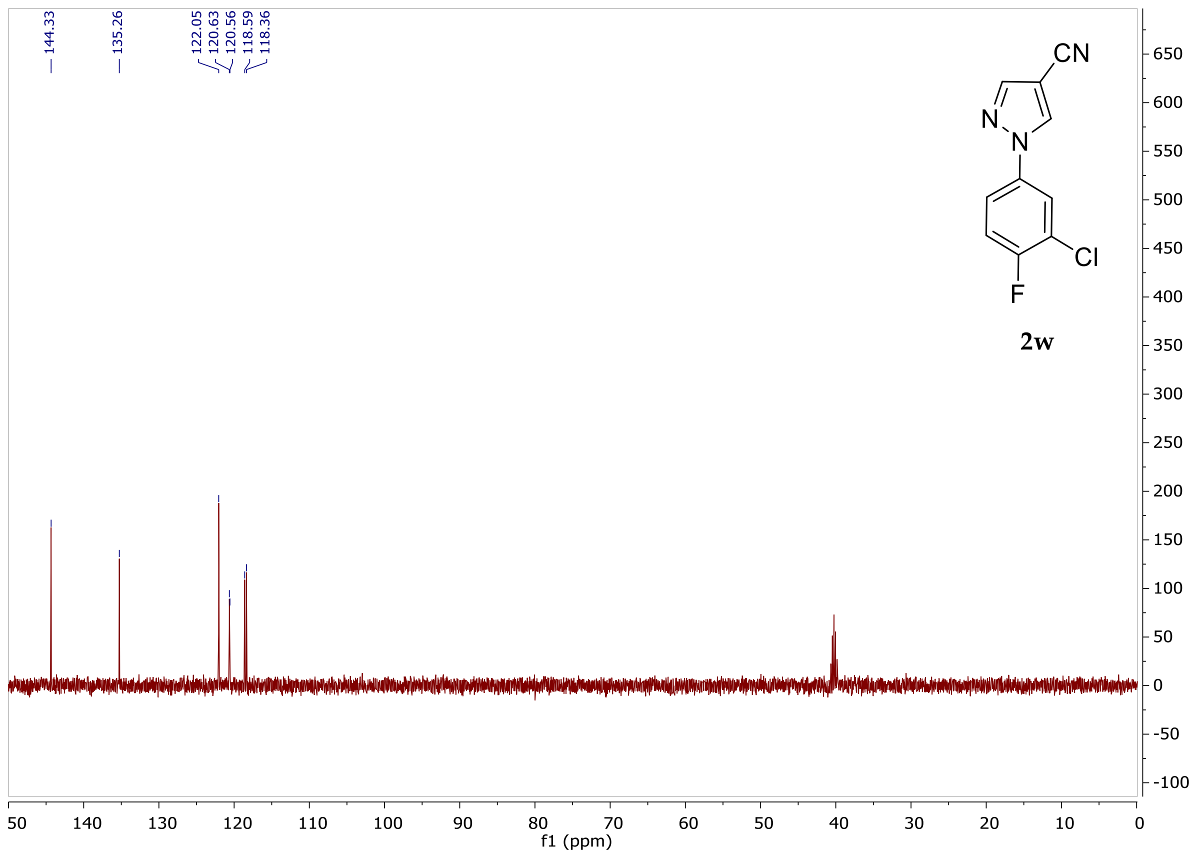
144.33
135.26
122.05
120.63
120.56
118.59
118.36
CN
N
N
Cl
F
2w
f1 (ppm)

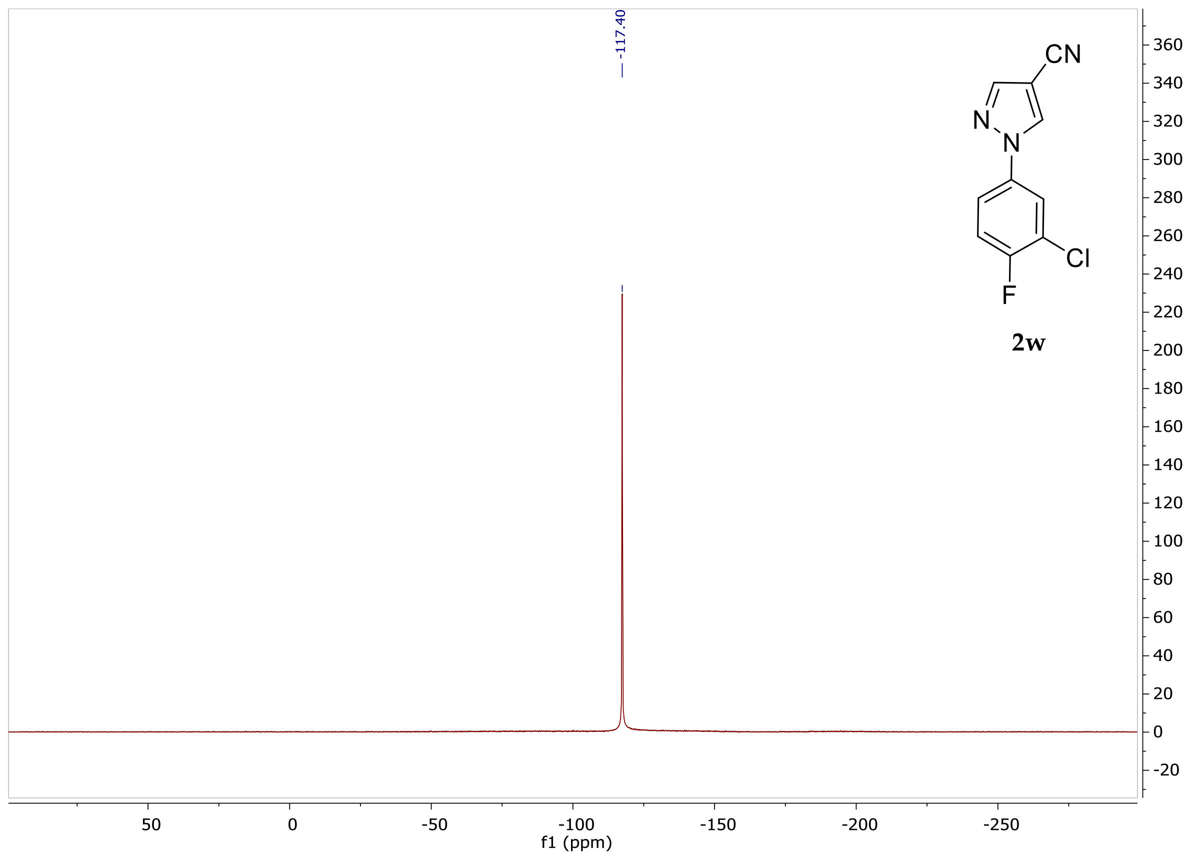
-117.40
CN
N
N
Cl
F
2w
f1 (ppm)

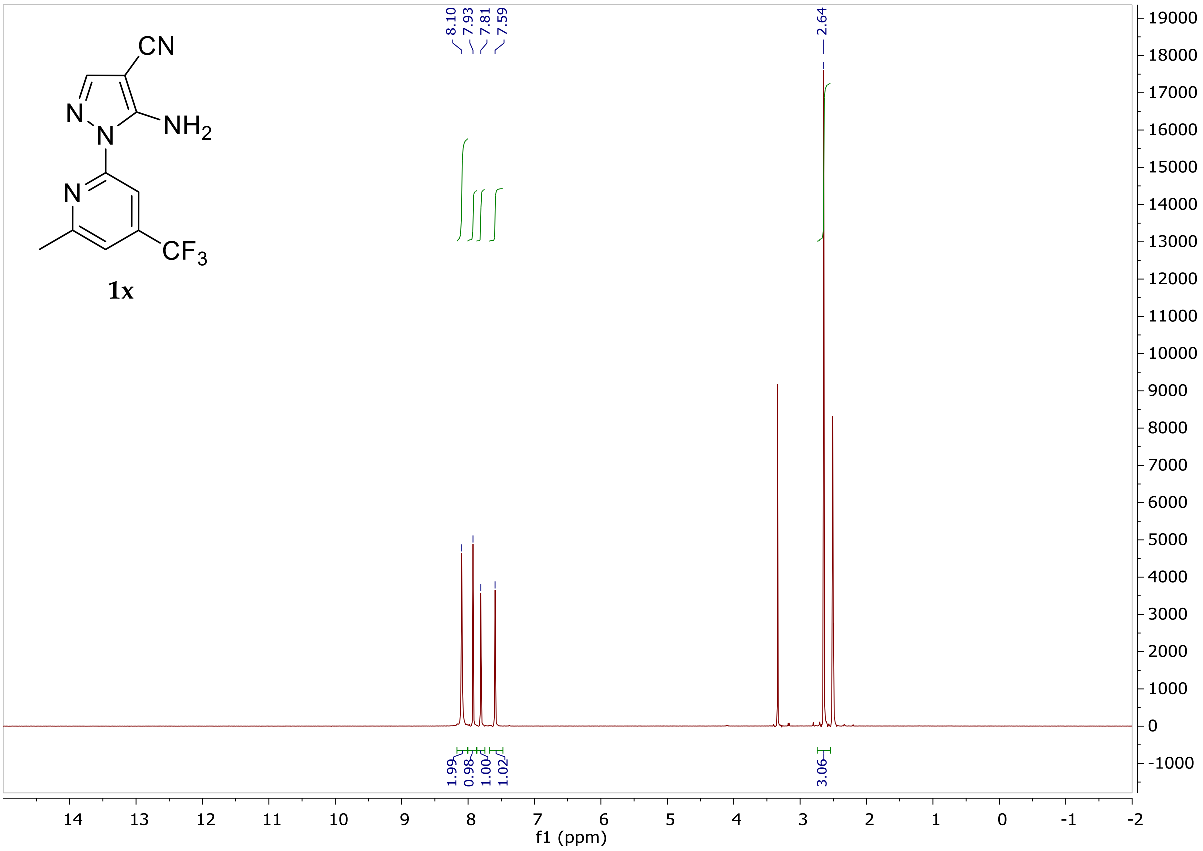
CN
N
N
NH2
N
CF3
1x
8.10
7.93
7.81
7.59
2.64
1.99
0.98
1.00
1.02
3.06
f1 (ppm)

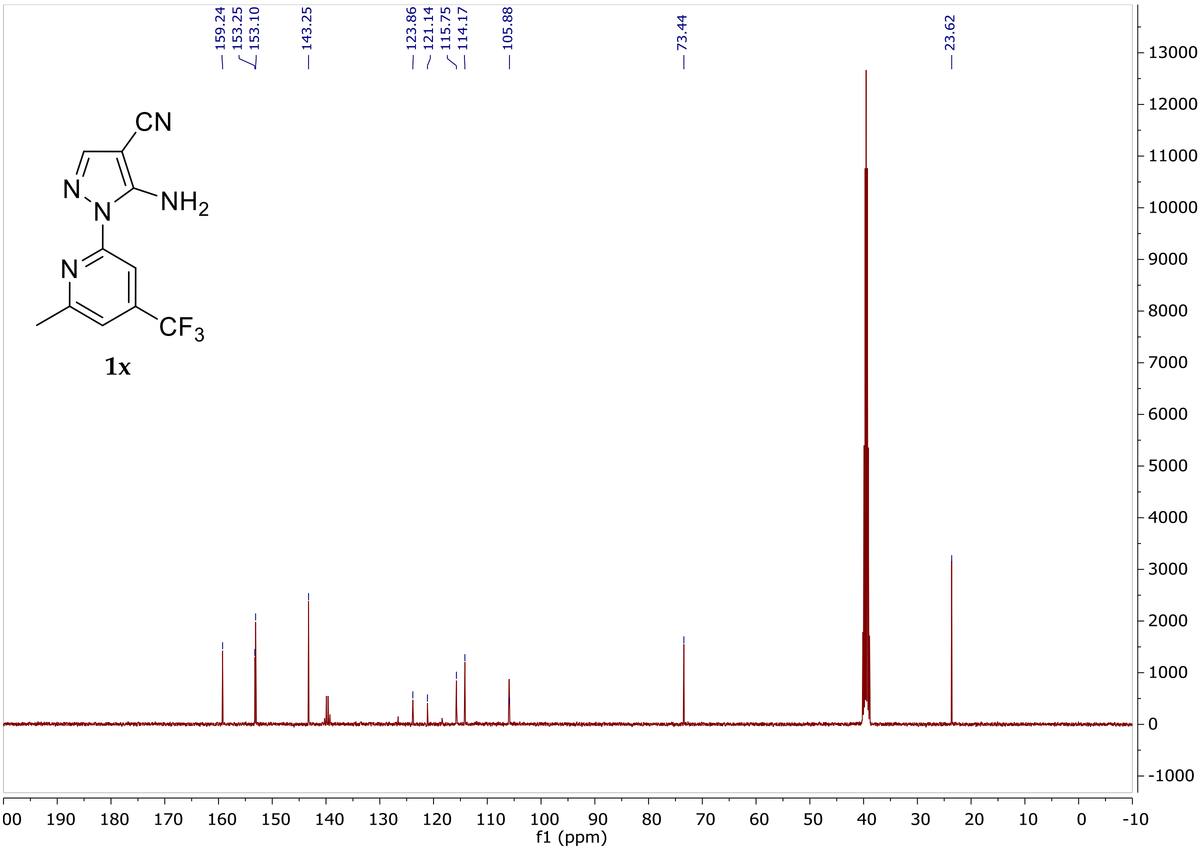
CN
N
N
NH2
N
CF3
1x
159.24
153.25
153.10
143.25
123.86
121.14
115.75
114.17
105.88
73.44
23.62
f1 (ppm)

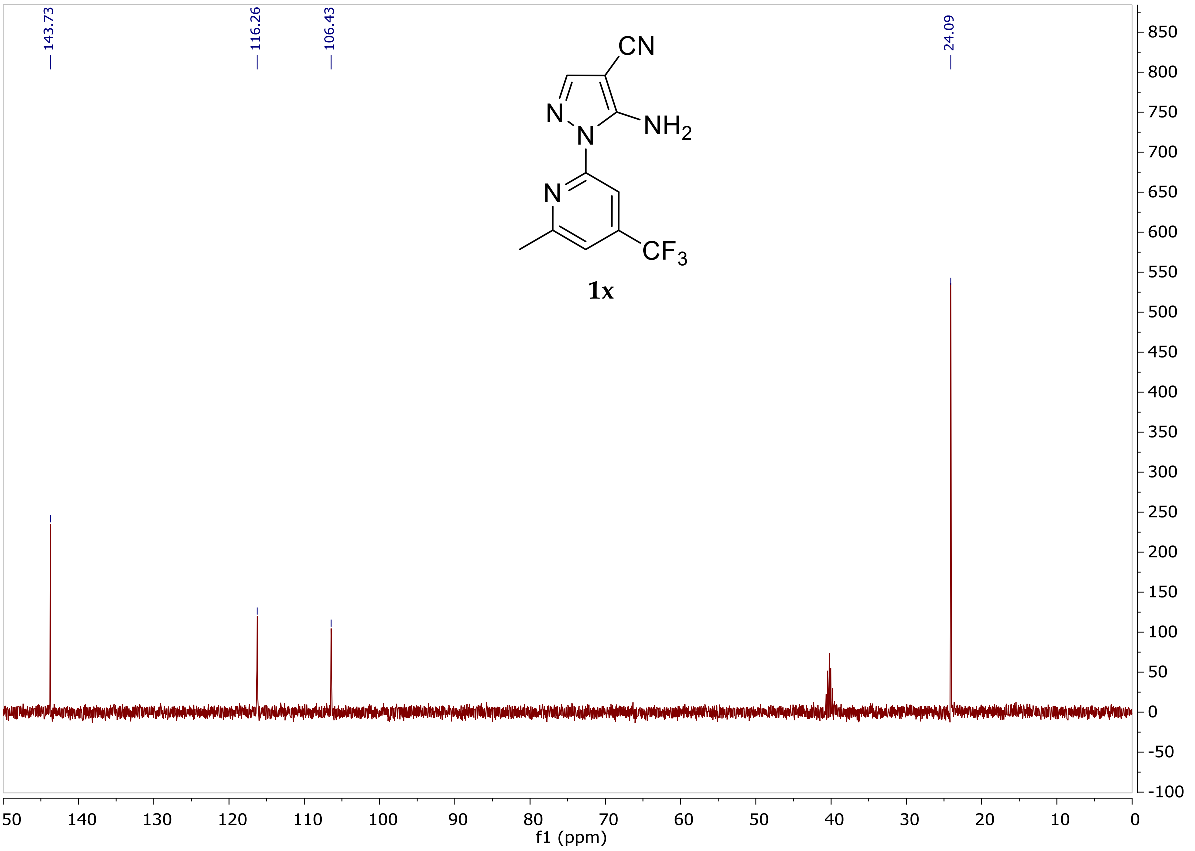
143.73
116.26
106.43
24.09
CN
NH2
N
N
N
CF3
1x
f1 (ppm)

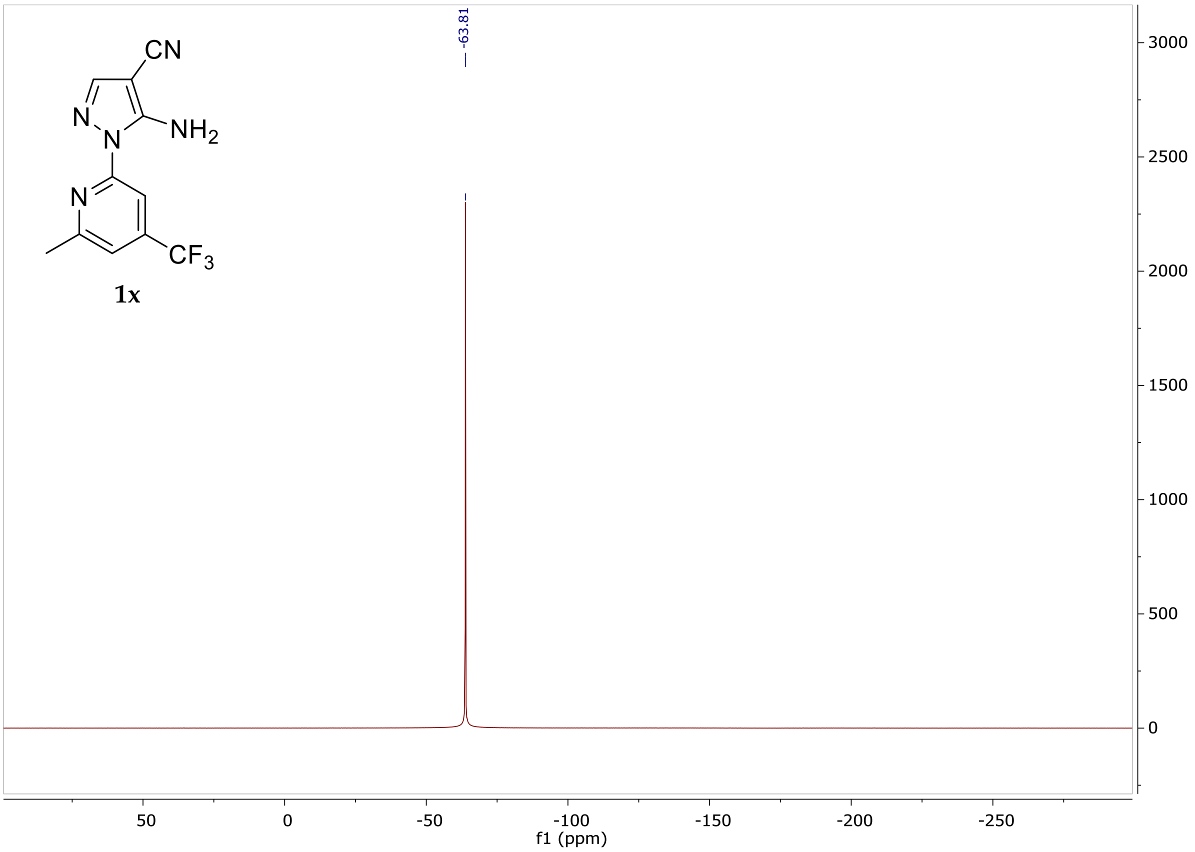
-63.81
CN
NH2
N
N
N
CF3
1x
f1 (ppm)

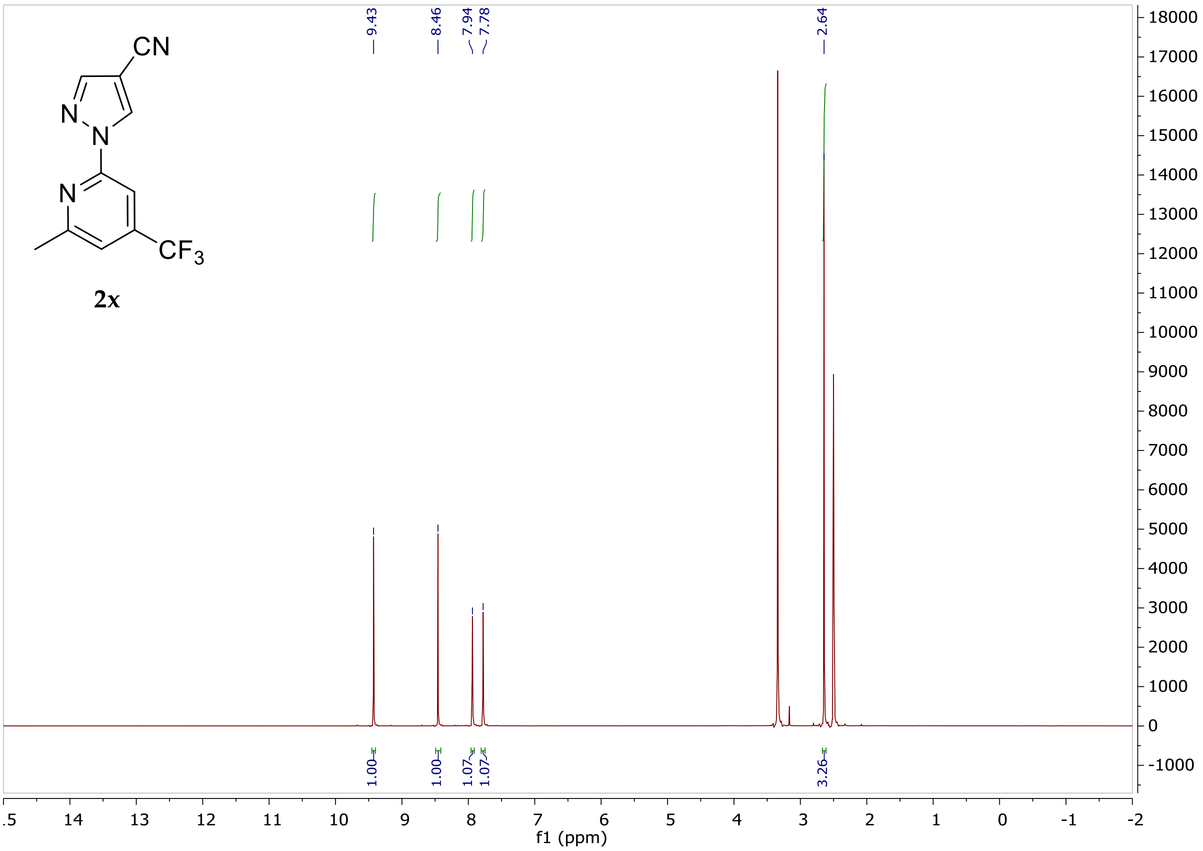
9.43
8.46
7.94
7.78
2.64
1.00
1.00
1.07
1.07
3.26
CN
N
N
N
CF3
2x
f1 (ppm)

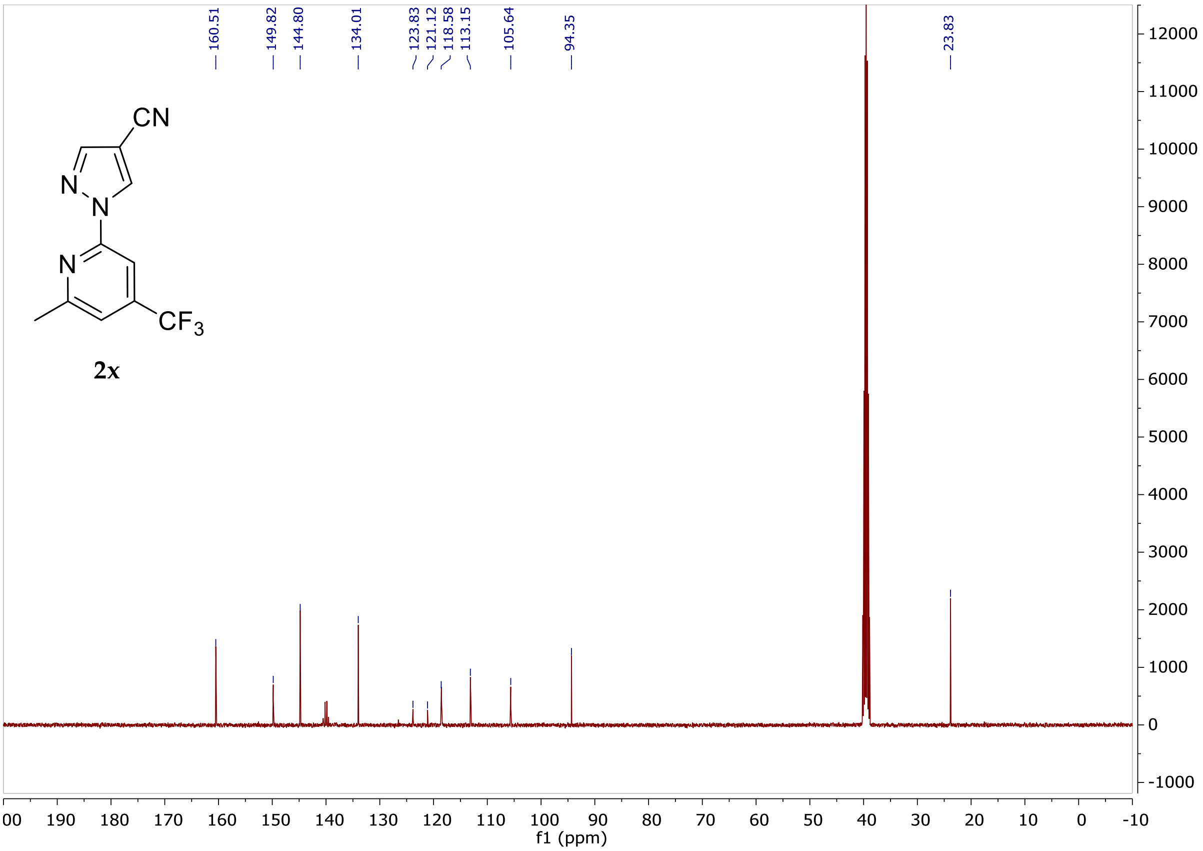
160.51
149.82
144.80
134.01
123.83
121.12
118.58
113.15
105.64
94.35
23.83
CN
N
N
N
CF3
2x
f1 (ppm)

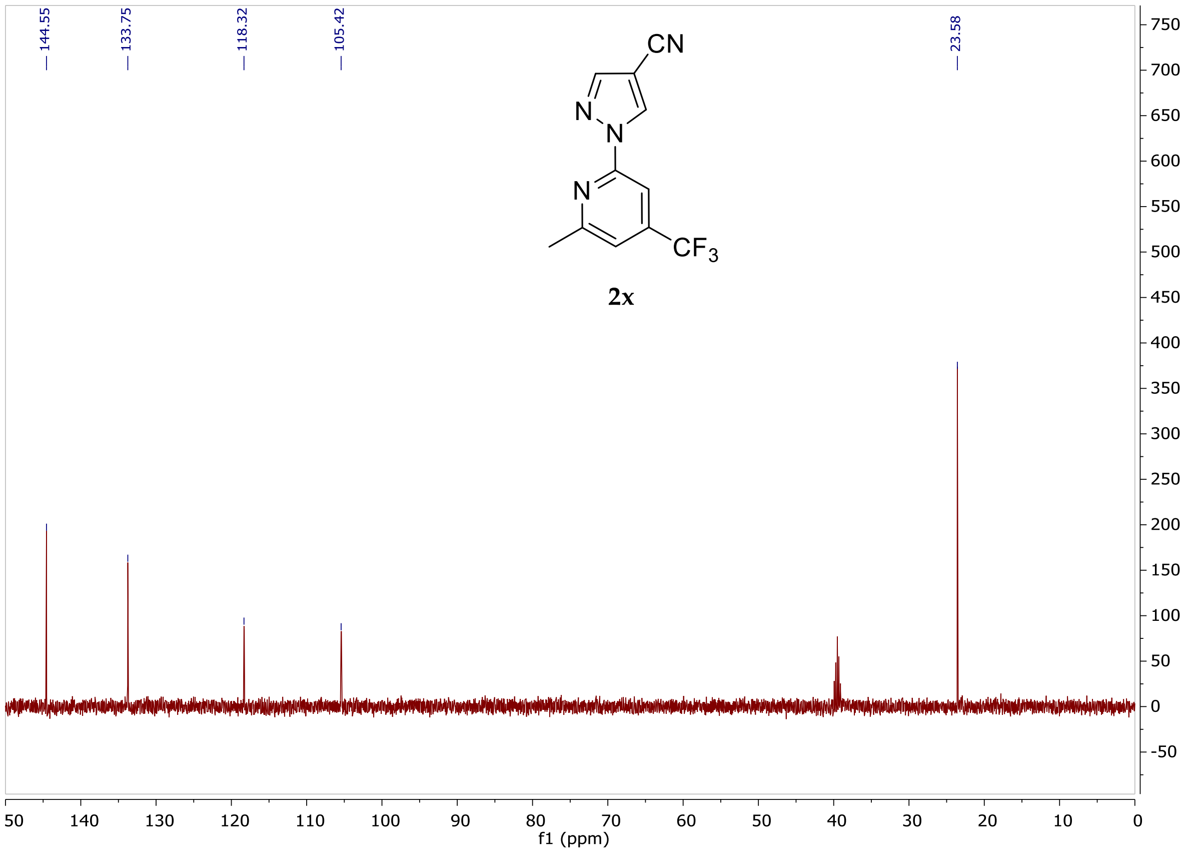
144.55
133.75
118.32
105.42
23.58
CN
N
N
N
CF3
2x
f1 (ppm)

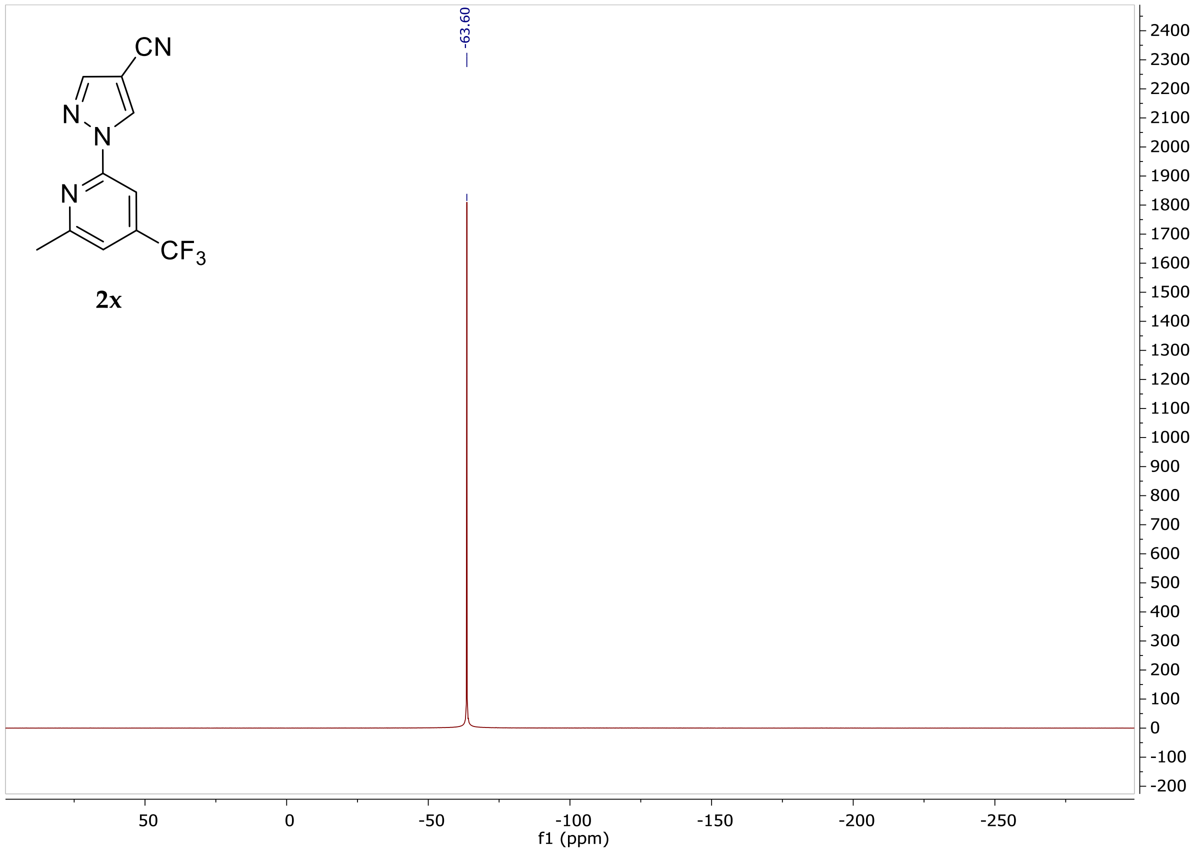
-63.60
CN
N
N
N
CF3
2x
f1 (ppm)

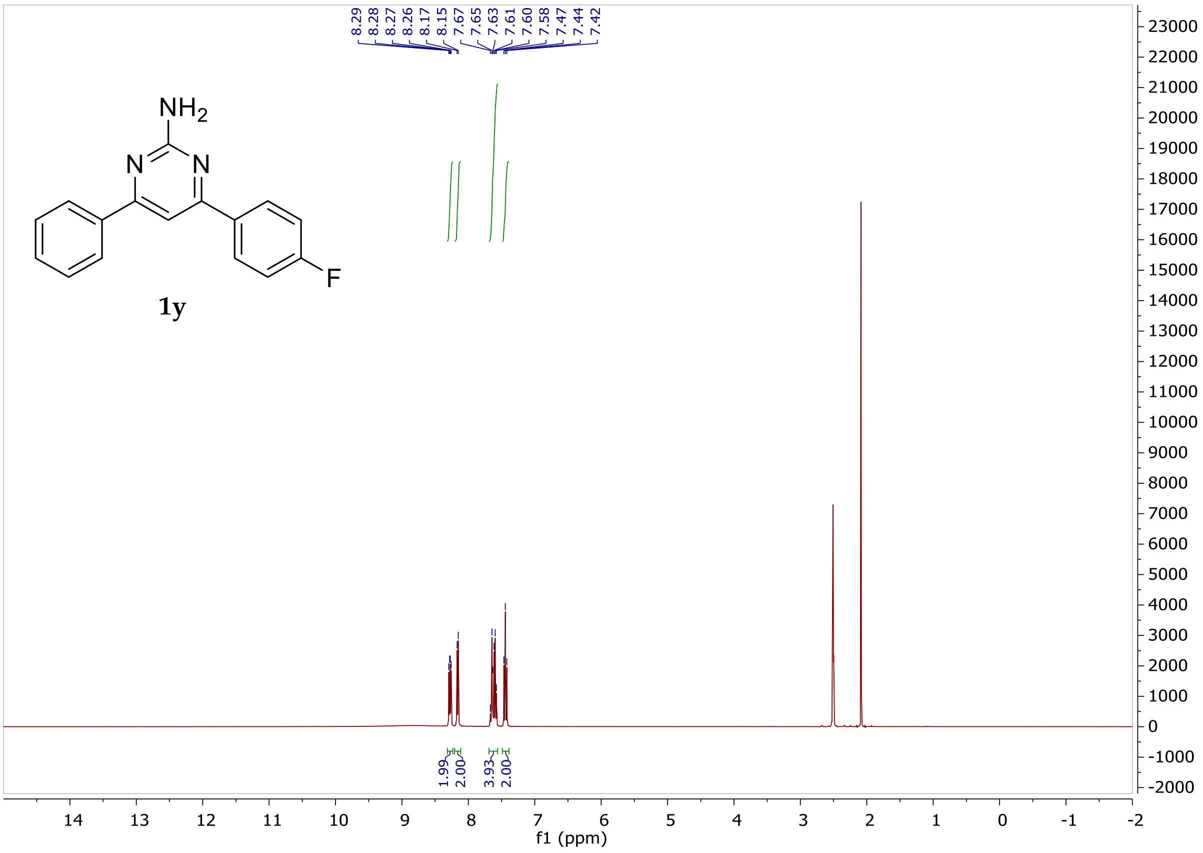

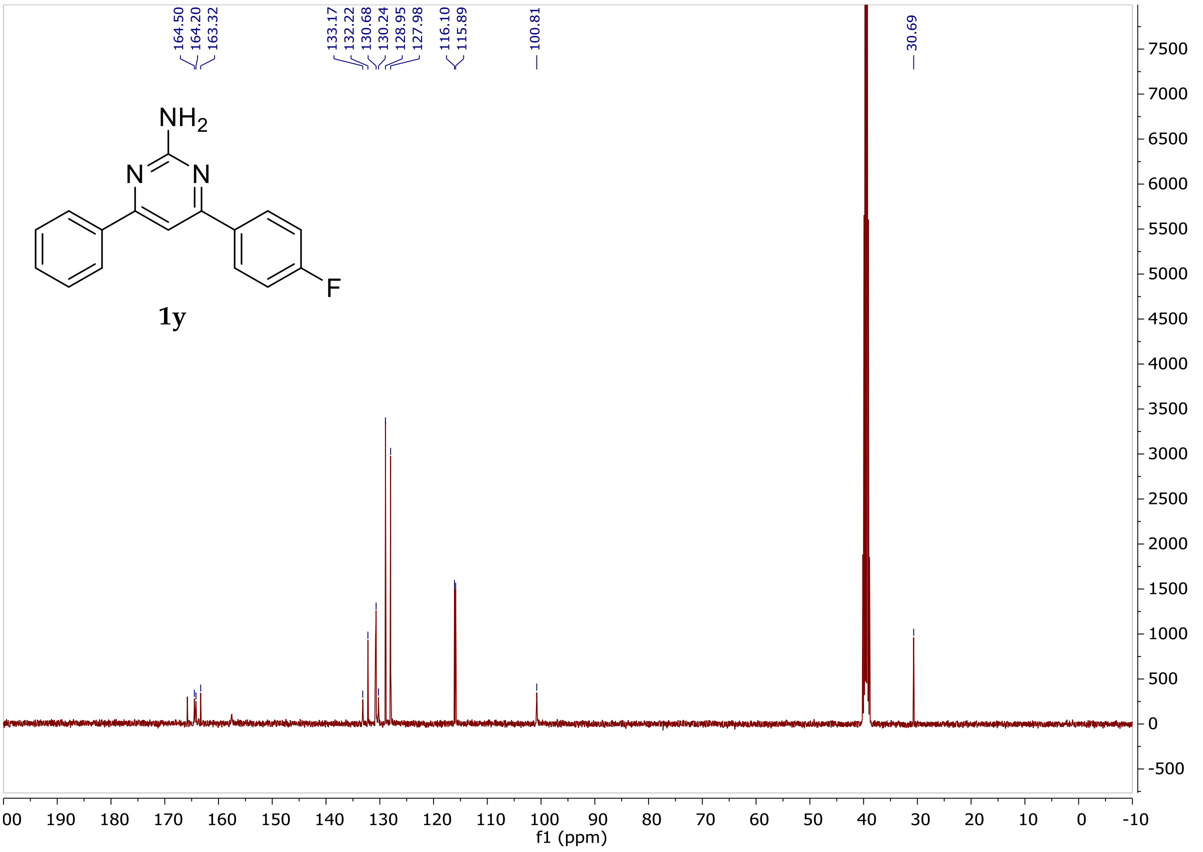

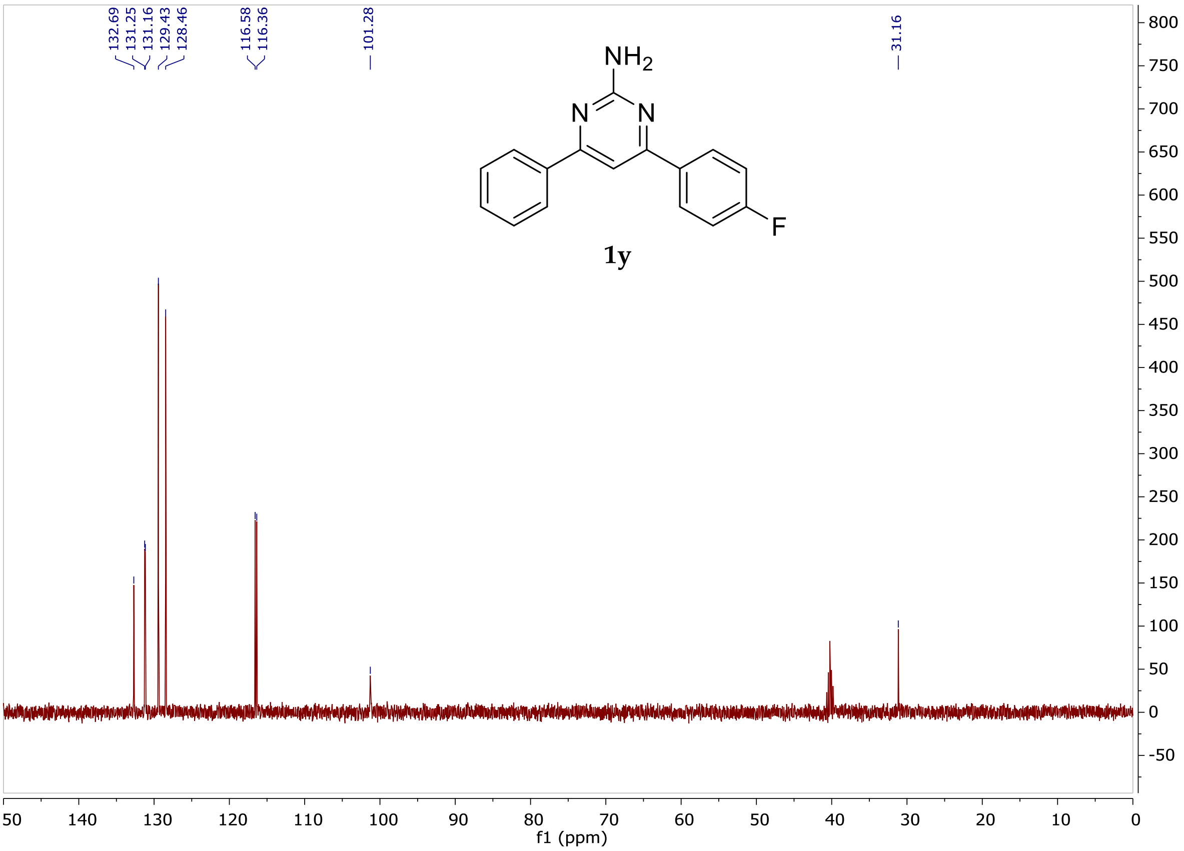
132.69
131.25
131.16
129.43
128.46
116.58
116.36
101.28
31.16
NH2
N
N
F
1y
f1 (ppm)

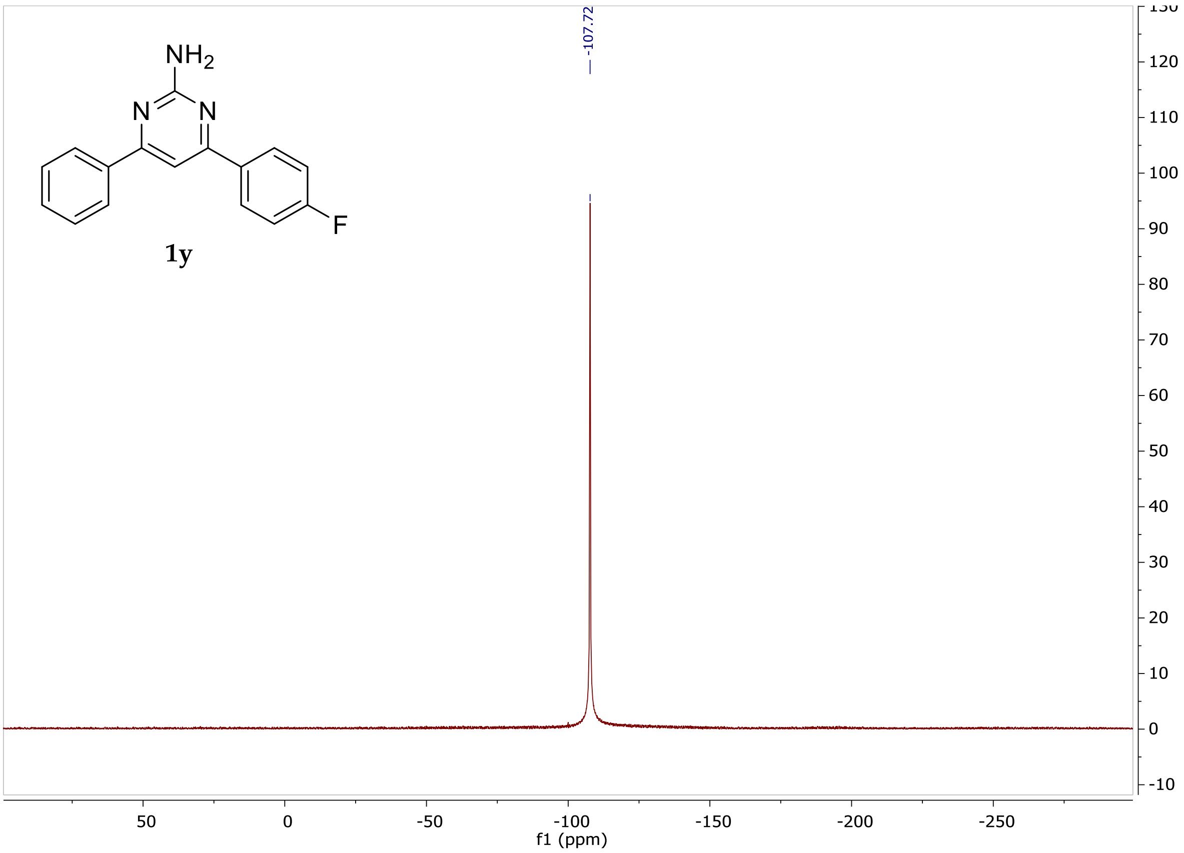
-107.72
NH2
N
N
F
1y
f1 (ppm)

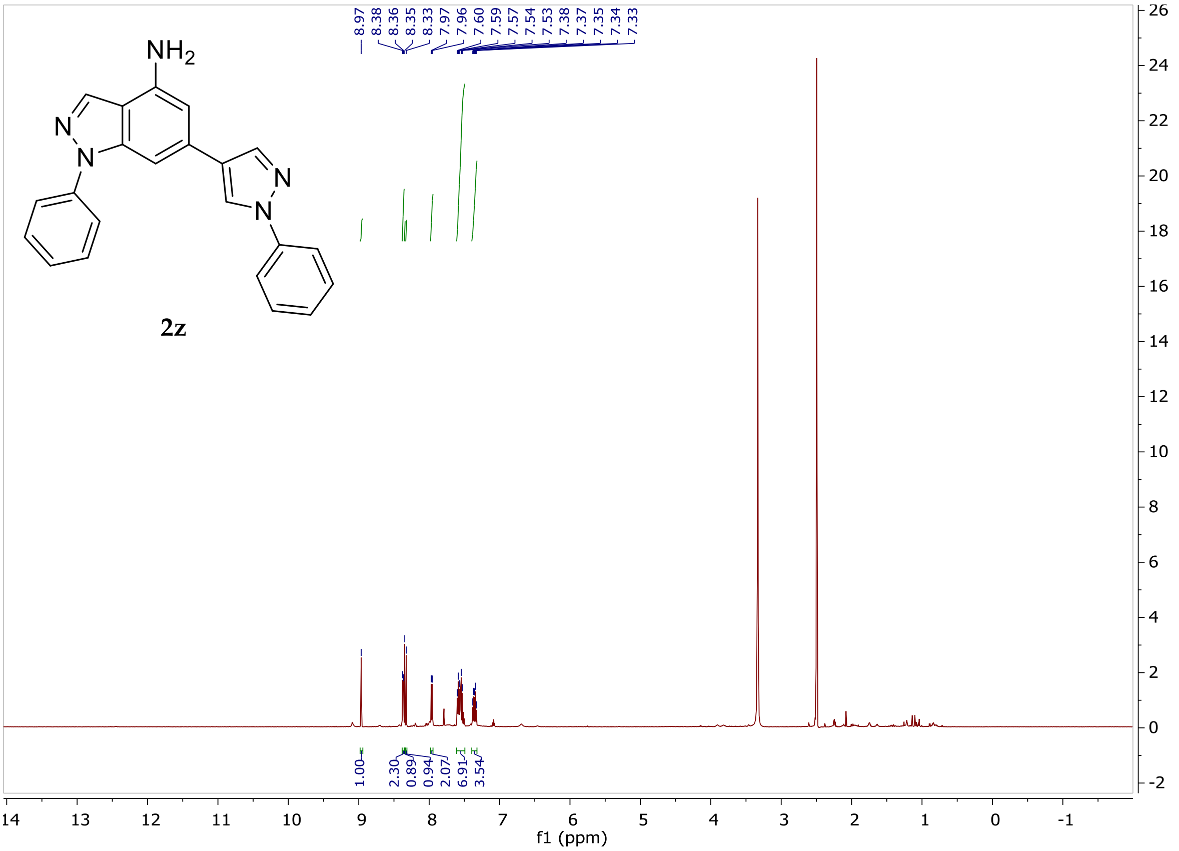
8.97
8.38
8.36
8.35
8.33
7.97
7.96
7.60
7.59
7.57
7.54
7.53
7.38
7.37
7.35
7.34
7.33
NH2
N
N
N
N
2z
1.00
2.30
0.89
0.94
2.07
6.91
3.54
f1 (ppm)

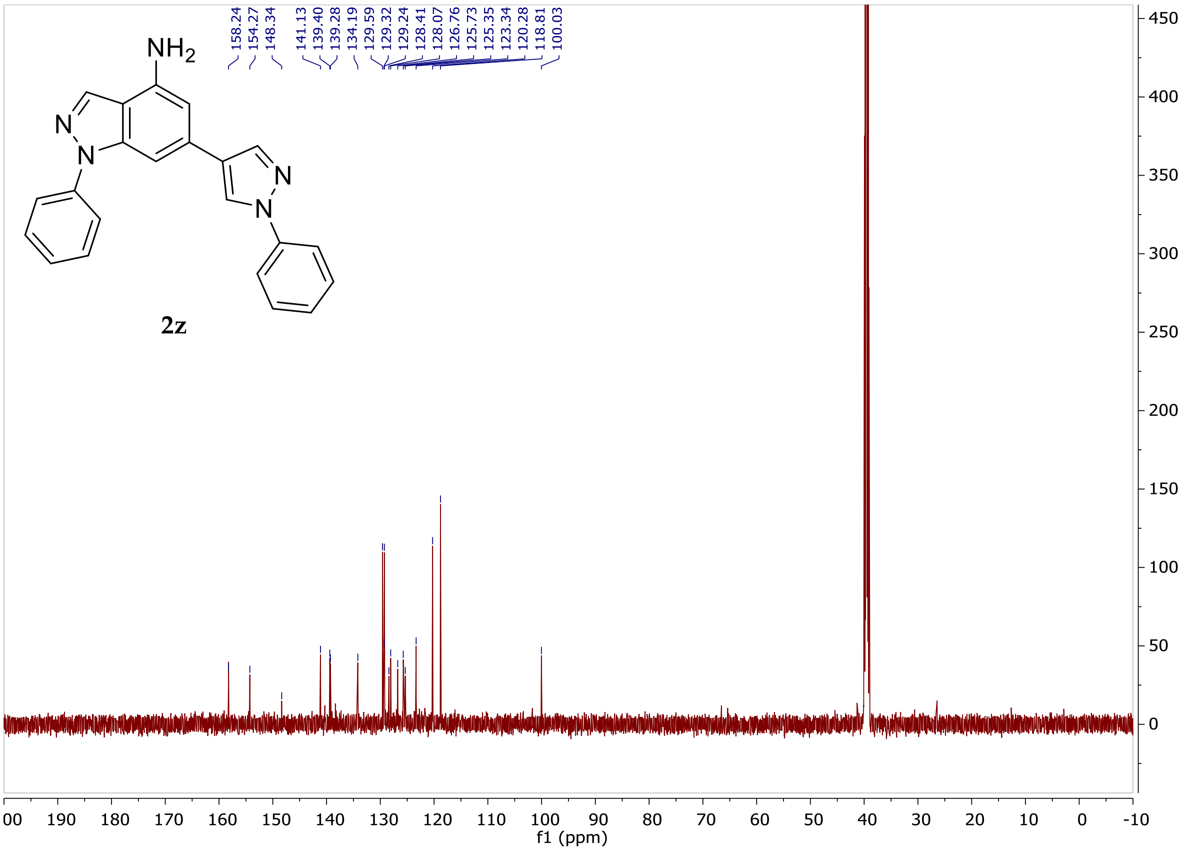
158.24
154.27
148.34
141.13
139.40
139.28
134.19
129.59
129.32
129.24
128.41
128.07
126.76
125.73
125.35
123.34
120.28
118.81
100.03
NH2
N
N
N
N
2z
f1 (ppm)

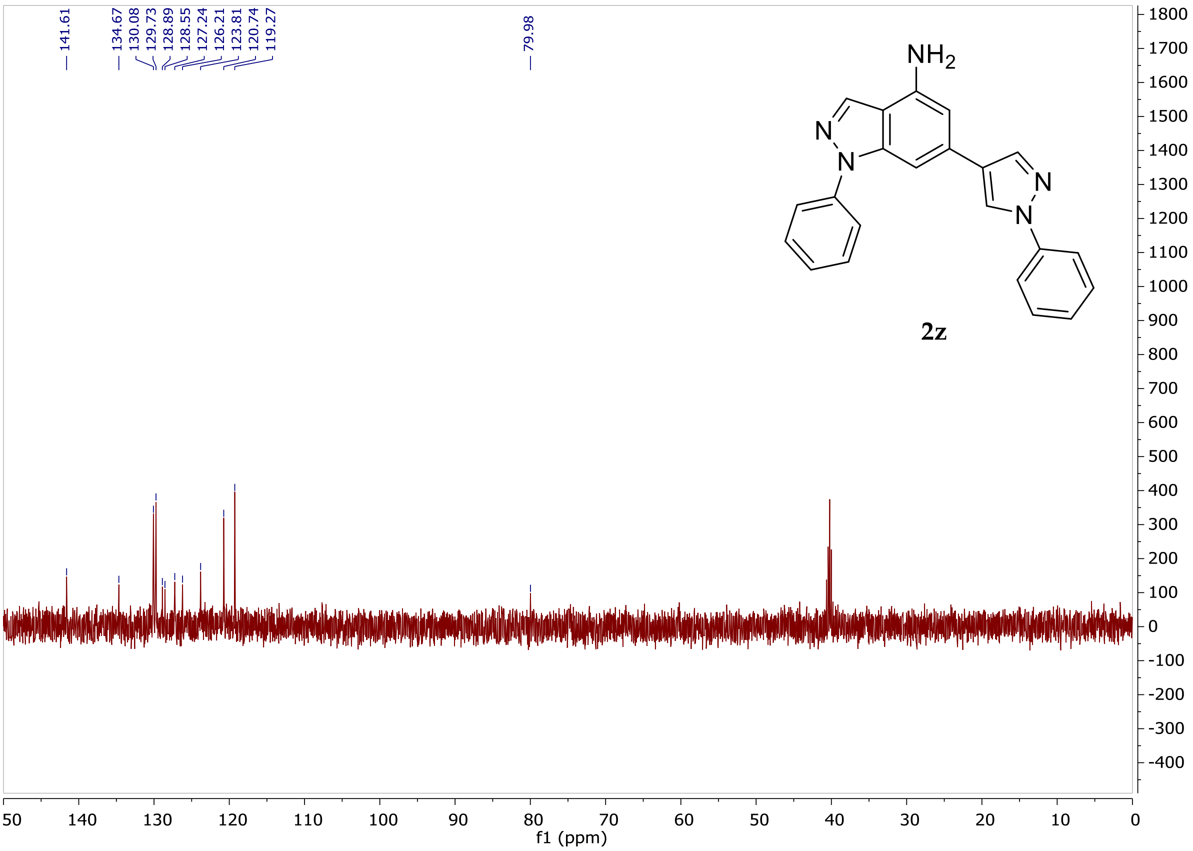
141.61
134.67
130.08
129.73
128.89
128.55
127.24
126.21
123.81
120.74
119.27
79.98
NH2
N
N
N
N
2z
f1 (ppm)

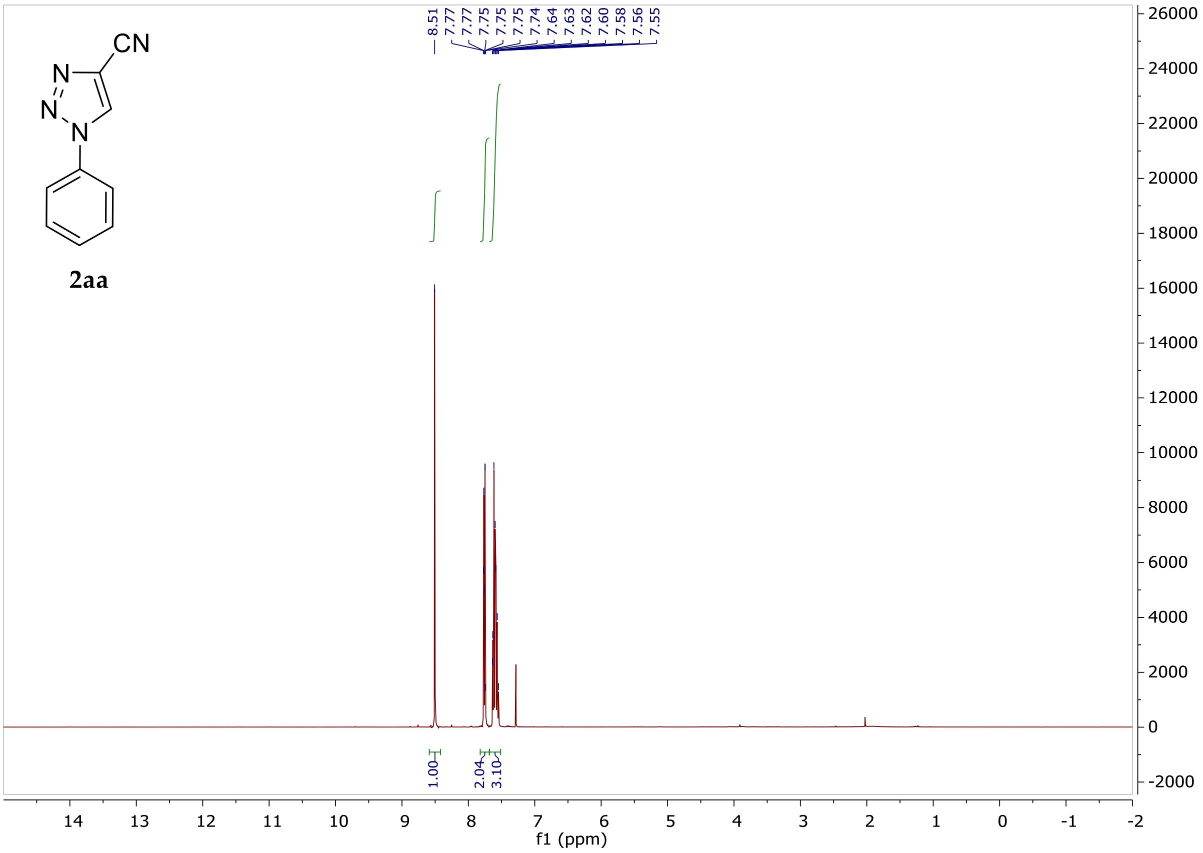
CN
N
N
N
2aa
8.51
7.77
7.77
7.75
7.75
7.75
7.74
7.64
7.63
7.62
7.60
7.58
7.56
7.55
1.00
2.04
3.10
f1 (ppm)

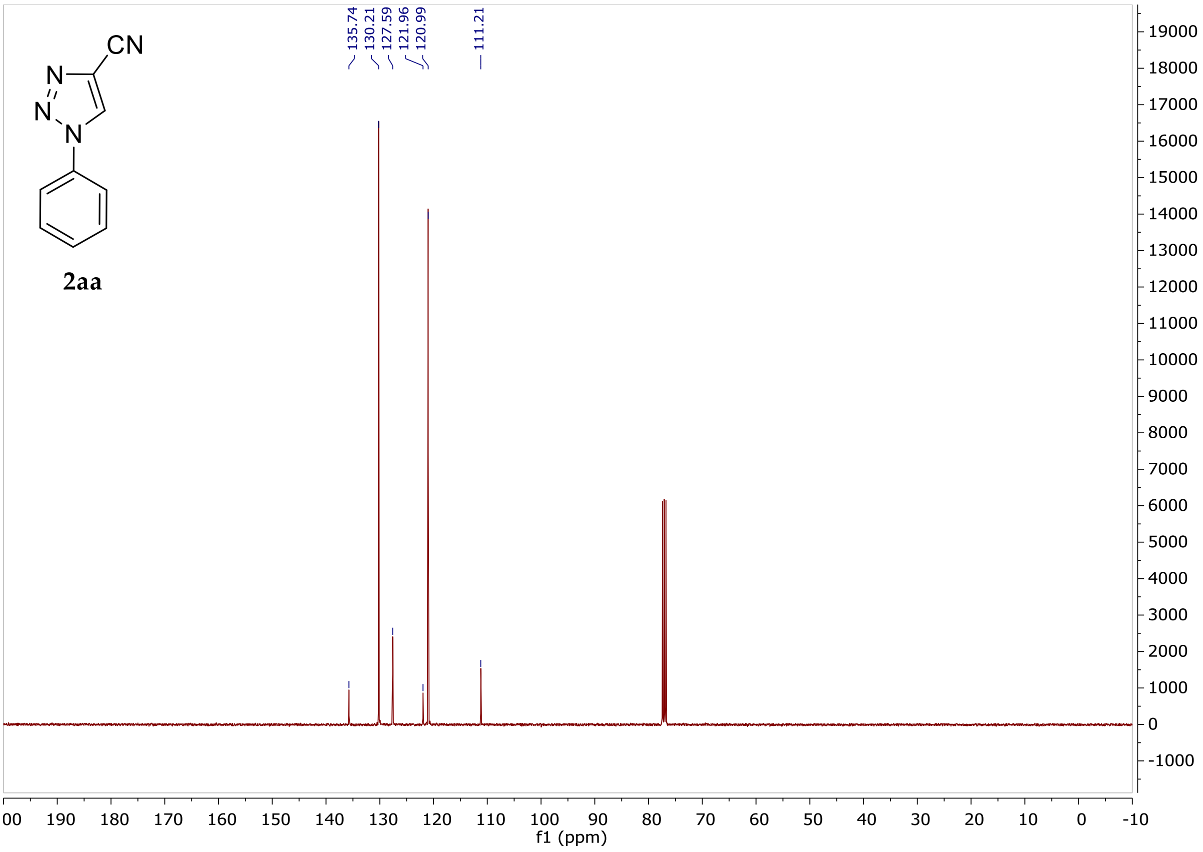
CN
N
N
N
2aa
135.74
130.21
127.59
121.96
120.99
111.21
f1 (ppm)

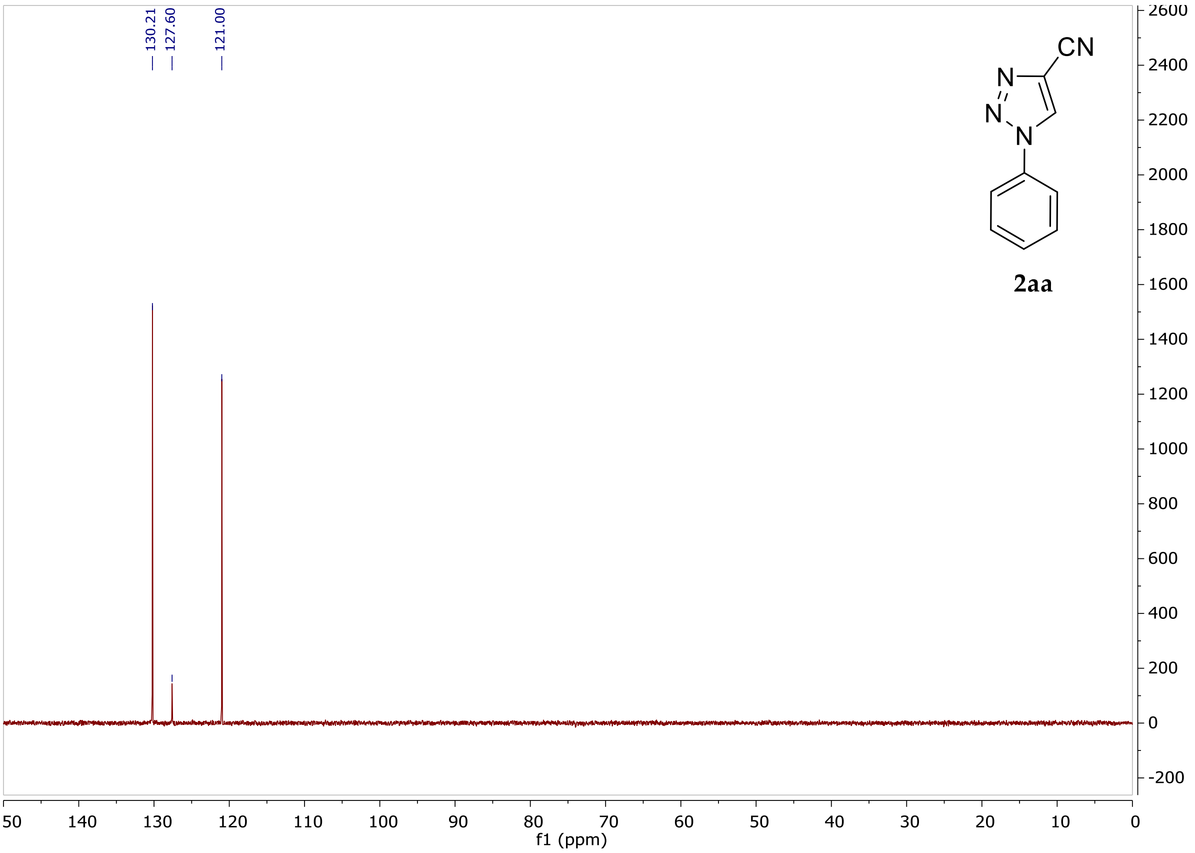
130.21
127.60
121.00
CN
N
N
N
2aa
2600
2400
2200
2000
1800
1600
1400
1200
1000
800
600
400
200
0
-200
50
140
130
120
110
100
90
80
70
60
50
40
30
20
10
0
f1 (ppm)

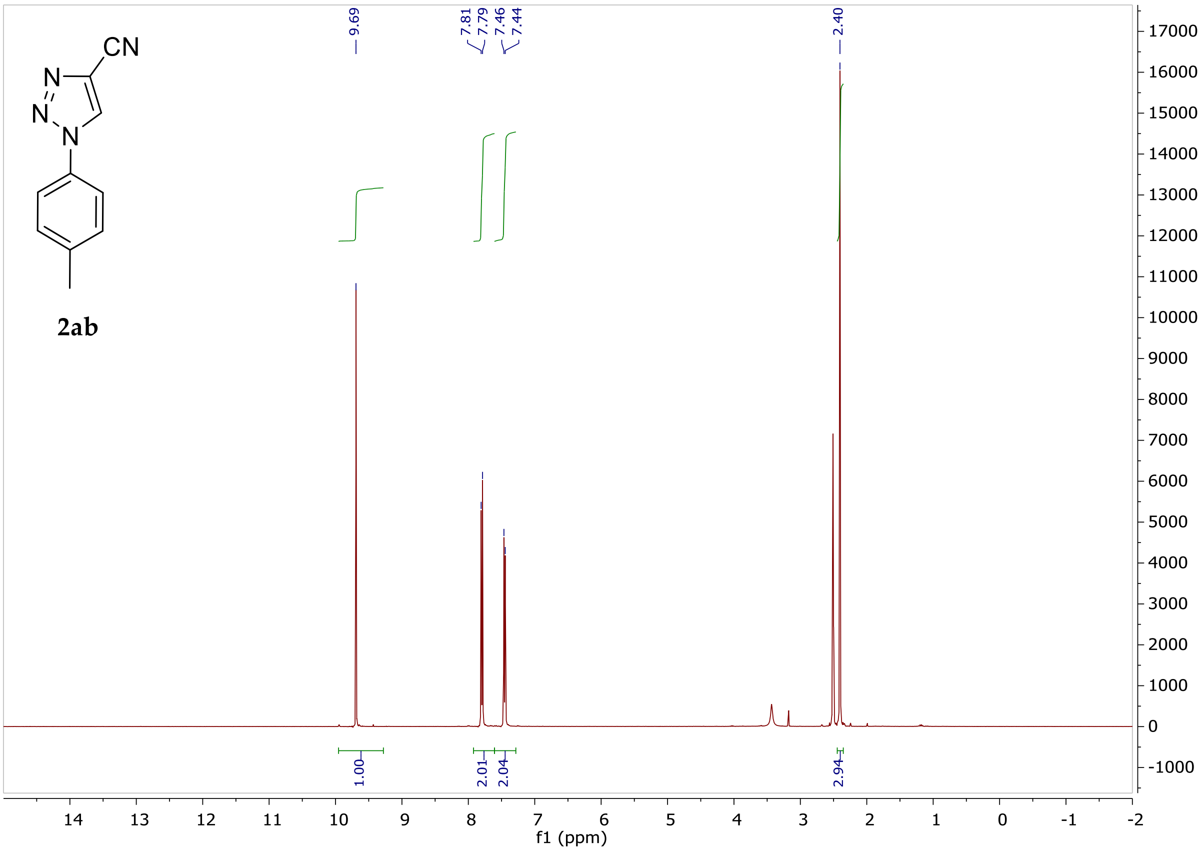
CN
N
N
N
2ab
9.69
7.81
7.79
7.46
7.44
2.40
1.00
2.01
2.04
2.94
f1 (ppm)

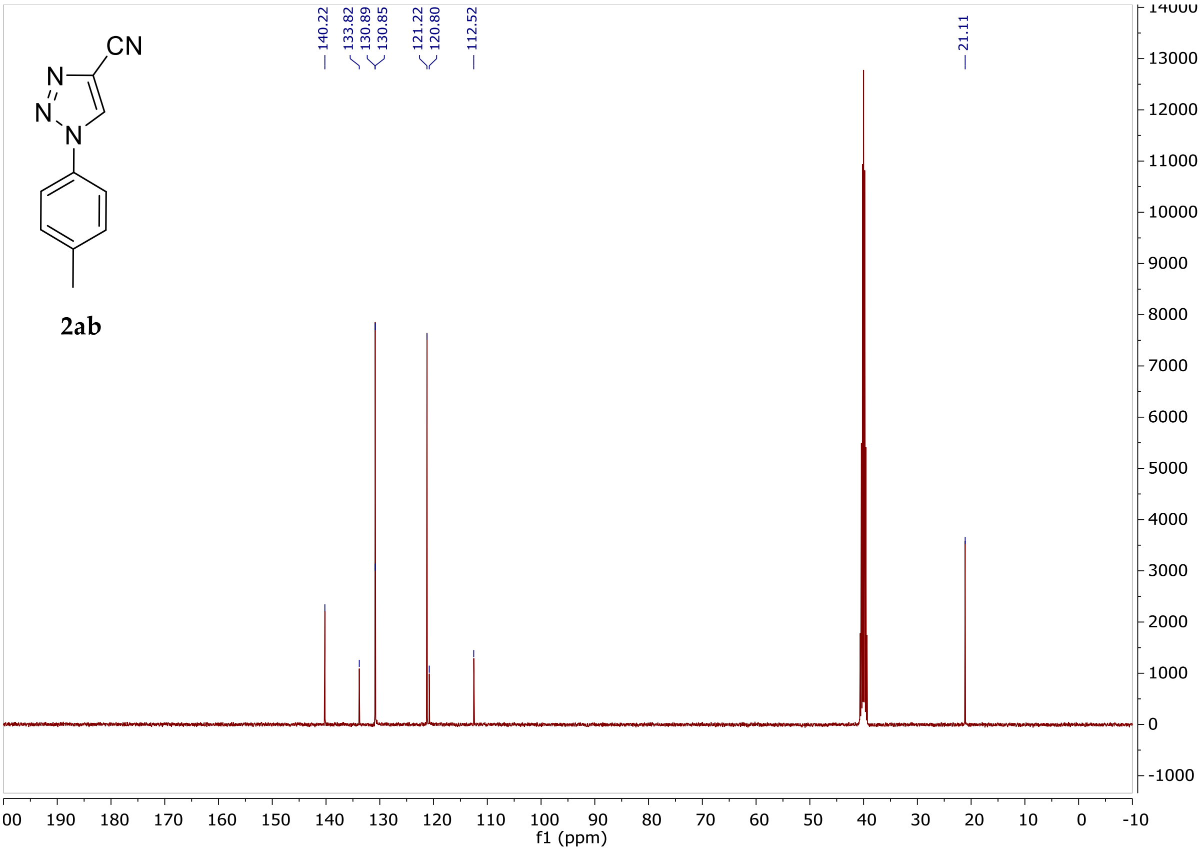
CN
N
N
N
2ab
140.22
133.82
130.89
130.85
121.22
120.80
112.52
21.11
f1 (ppm)

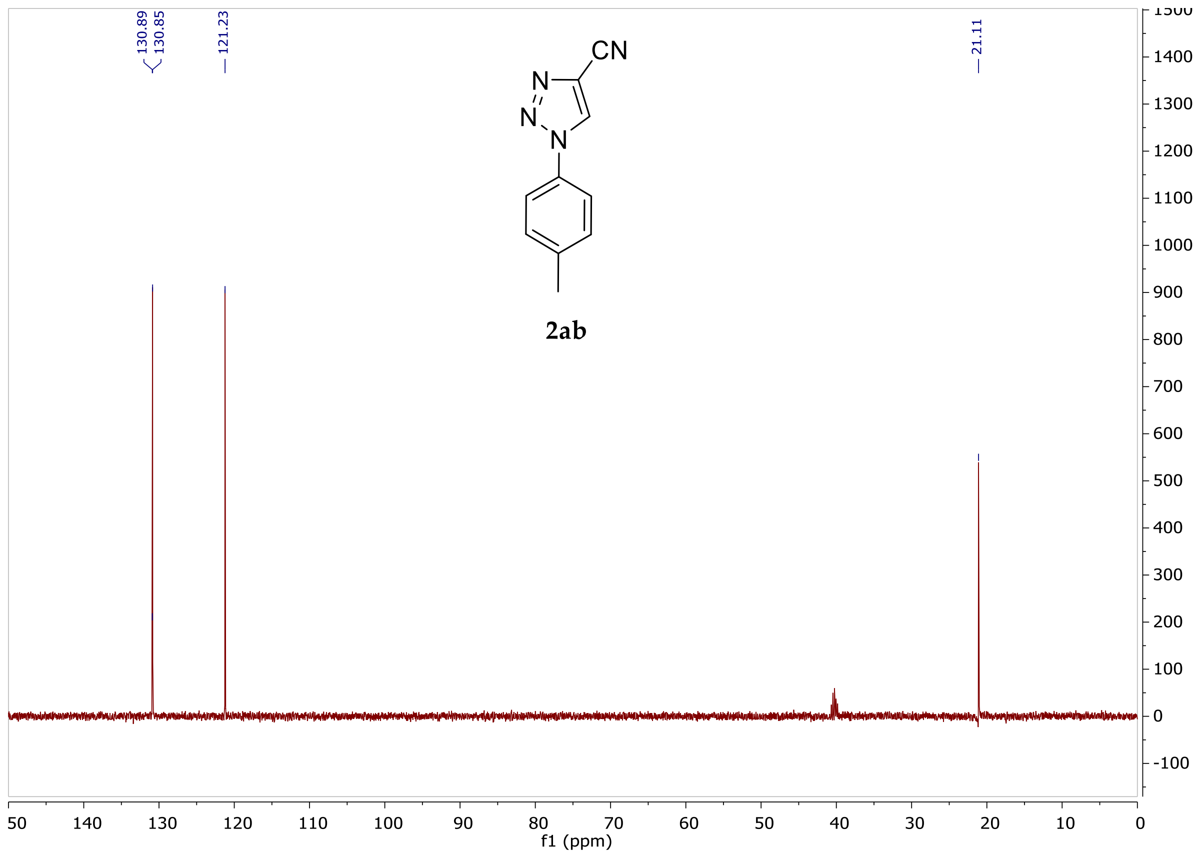
130.89
130.85
121.23
21.11
CN
N
N
N
2ab
f1 (ppm)

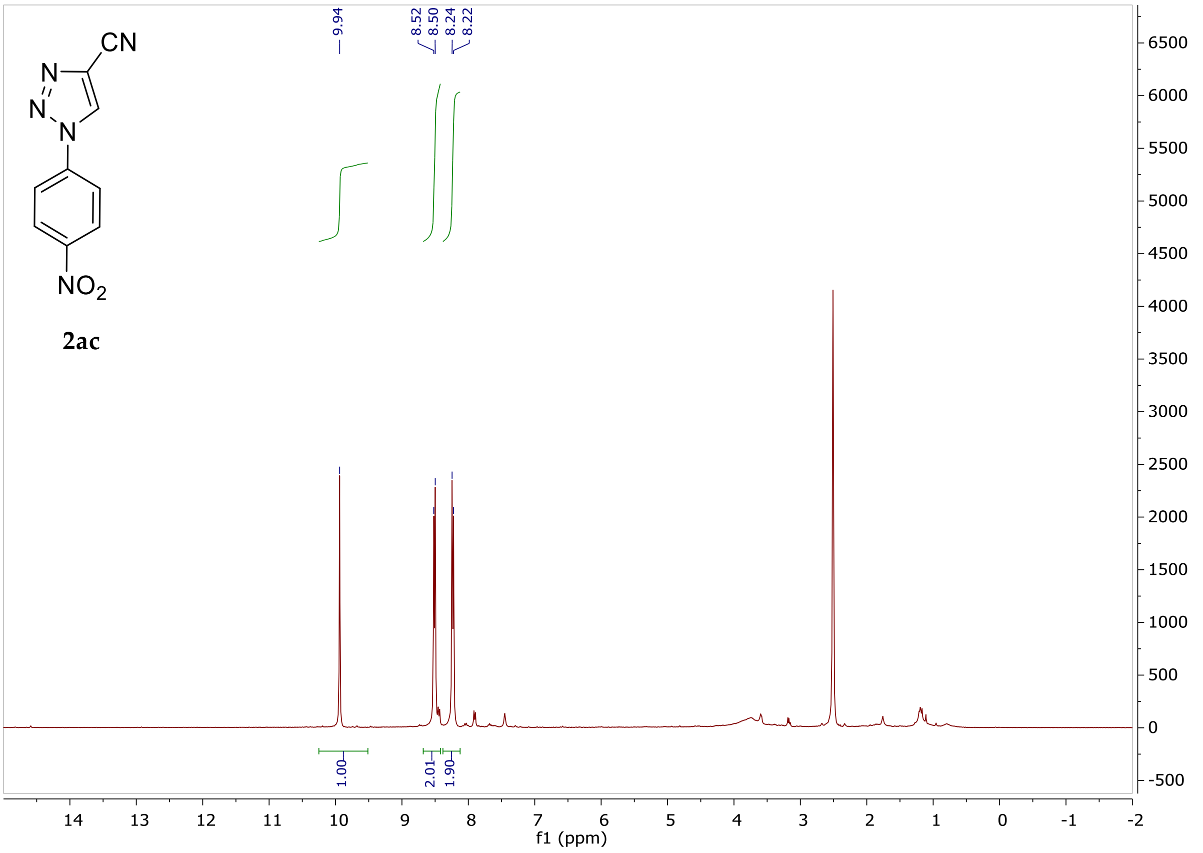

CN
N
N
N
NO2
2ac
9.94
8.52
8.50
8.24
8.22
1.00
2.01
1.90
f1 (ppm)

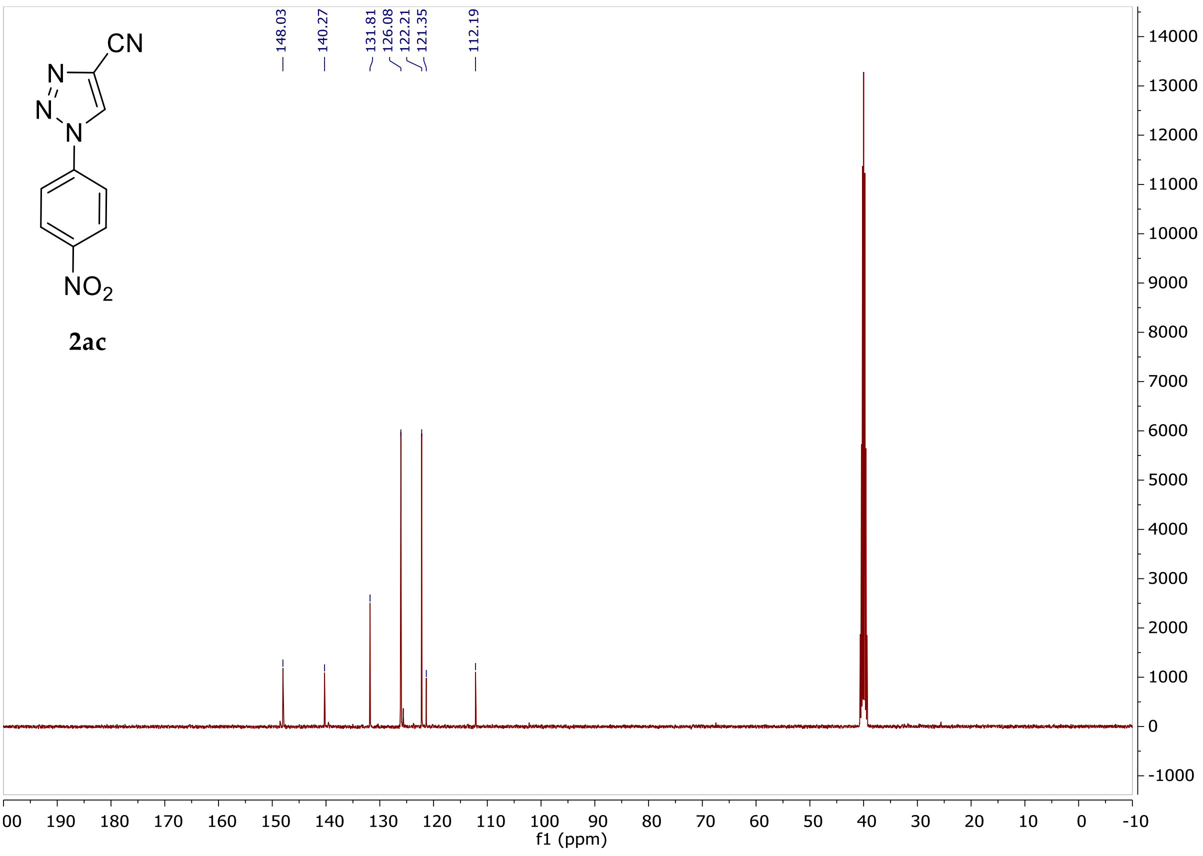

CN
N
N
N
NO2
2ac
148.03
140.27
131.81
126.08
122.21
121.35
112.19
f1 (ppm)

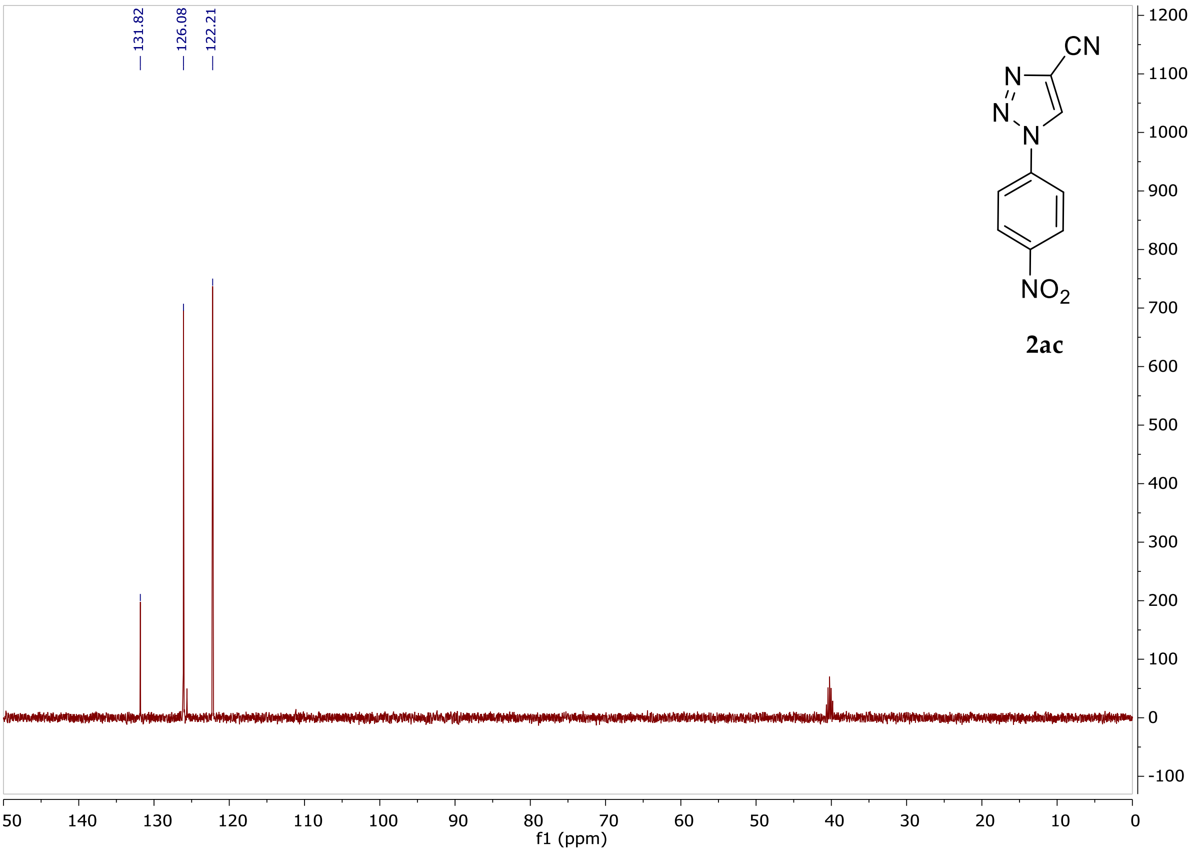

131.82
126.08
122.21
CN
N
N
N
NO2
2ac
f1 (ppm)

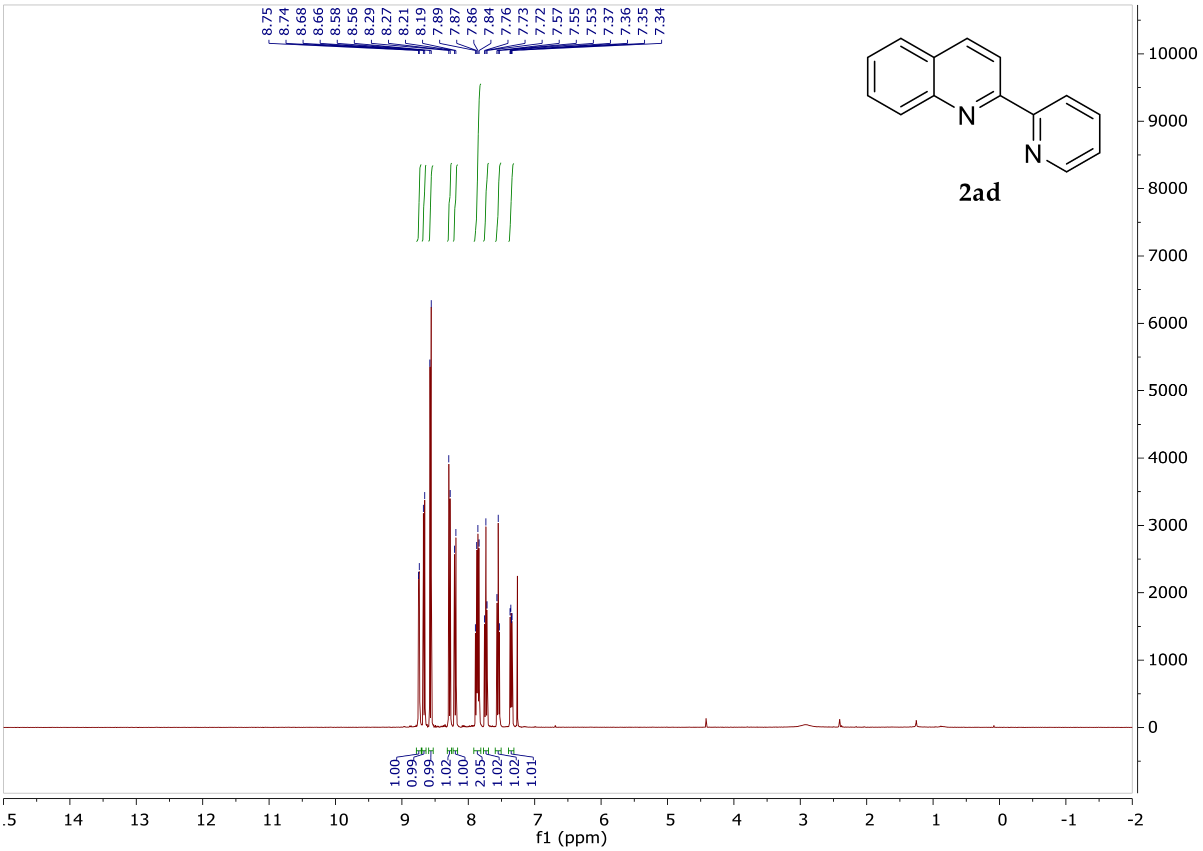
8.75
8.74
8.68
8.66
8.58
8.56
8.29
8.27
8.21
8.19
7.89
7.87
7.86
7.84
7.76
7.73
7.72
7.57
7.55
7.53
7.37
7.36
7.35
7.34
2ad
1.00
0.99
0.99
1.02
1.00
2.05
1.02
1.02
1.01
f1 (ppm)

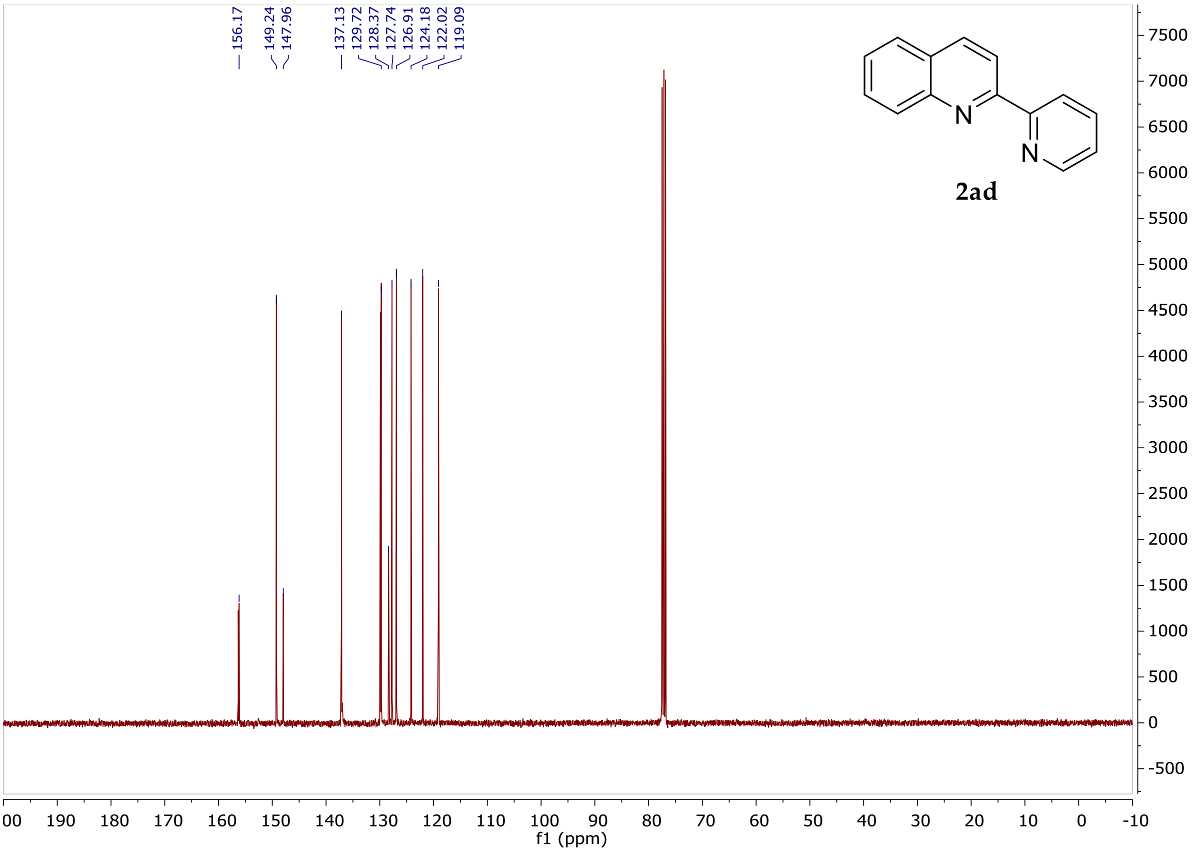
156.17
149.24
147.96
137.13
129.72
128.37
127.74
126.91
124.18
122.02
119.09
2ad
f1 (ppm)

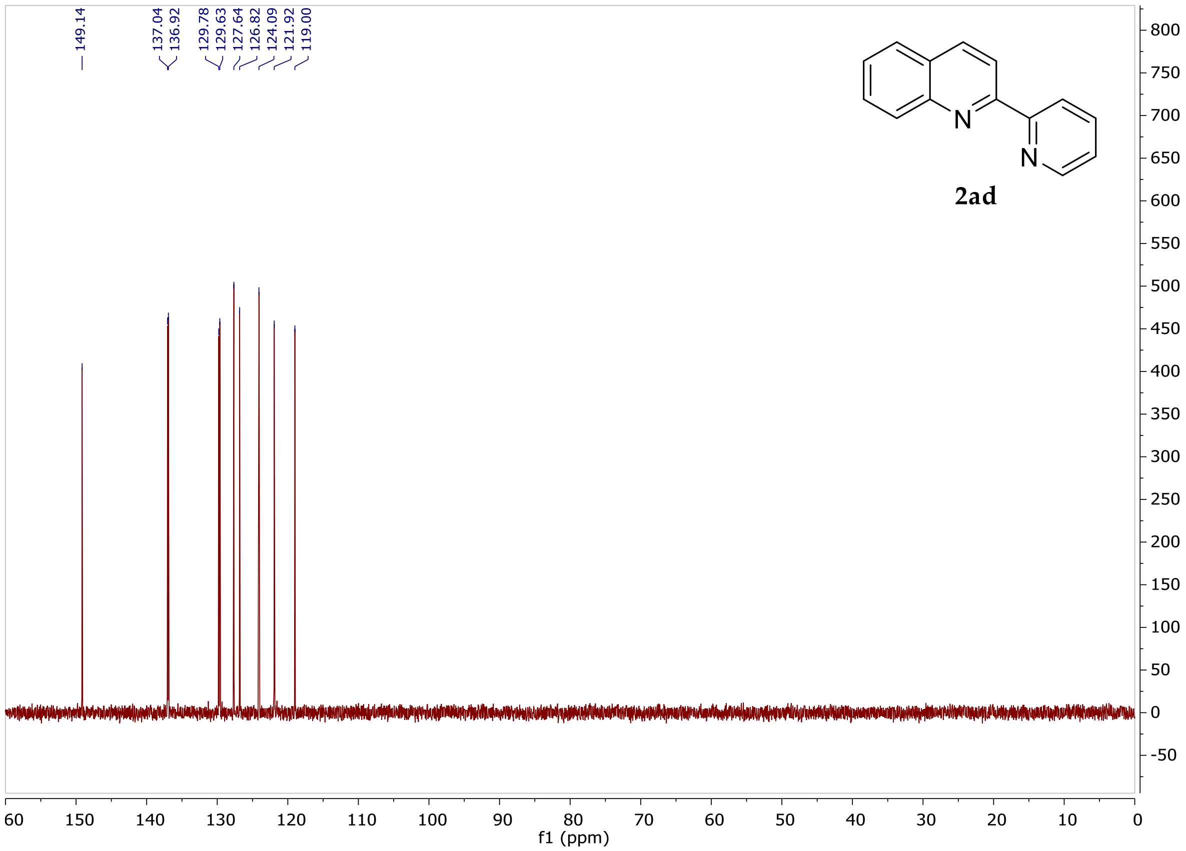

149.14
137.04
136.92
129.78
129.63
127.64
126.82
124.09
121.92
119.00
2ad
f1 (ppm)

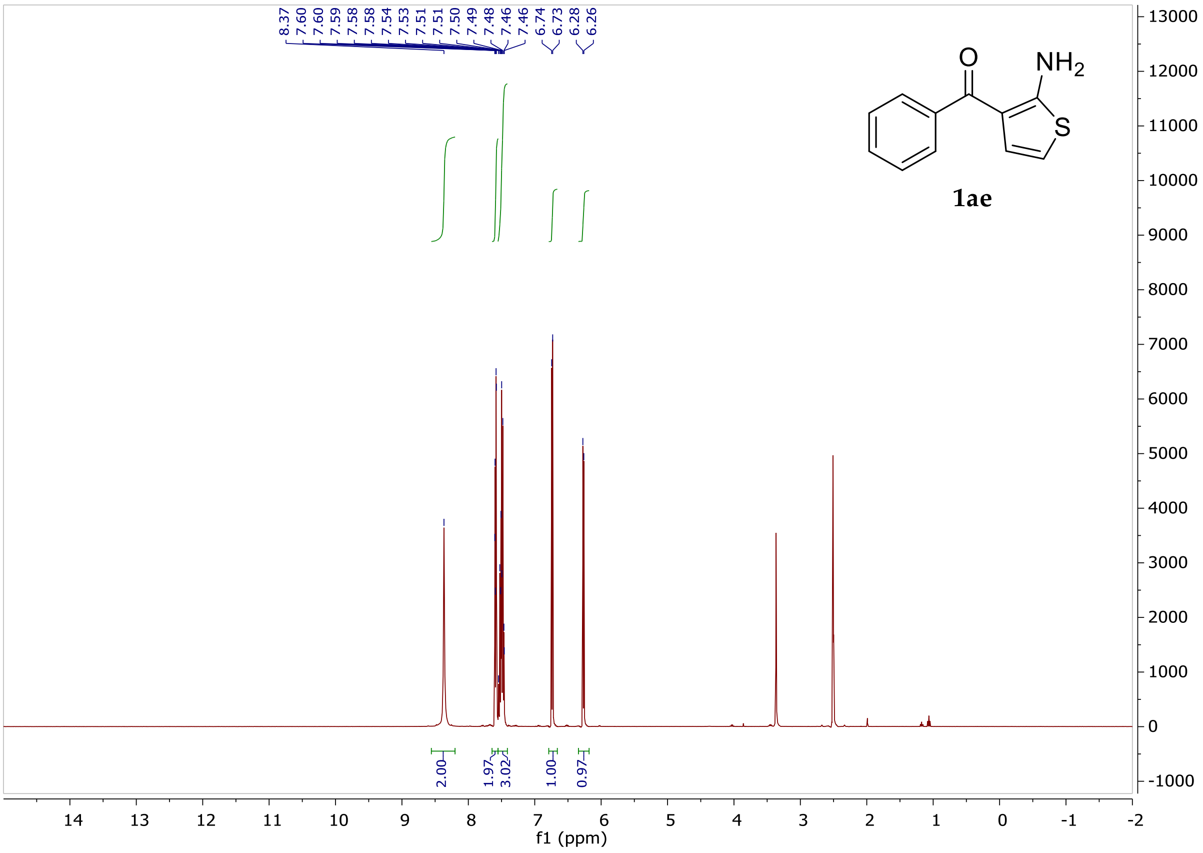
8.37
7.60
7.60
7.59
7.58
7.58
7.54
7.53
7.51
7.51
7.50
7.49
7.48
7.46
7.46
6.74
6.73
6.28
6.26
2.00
1.97
3.02
1.00
0.97
f1 (ppm)
NH2
O
S

1ae

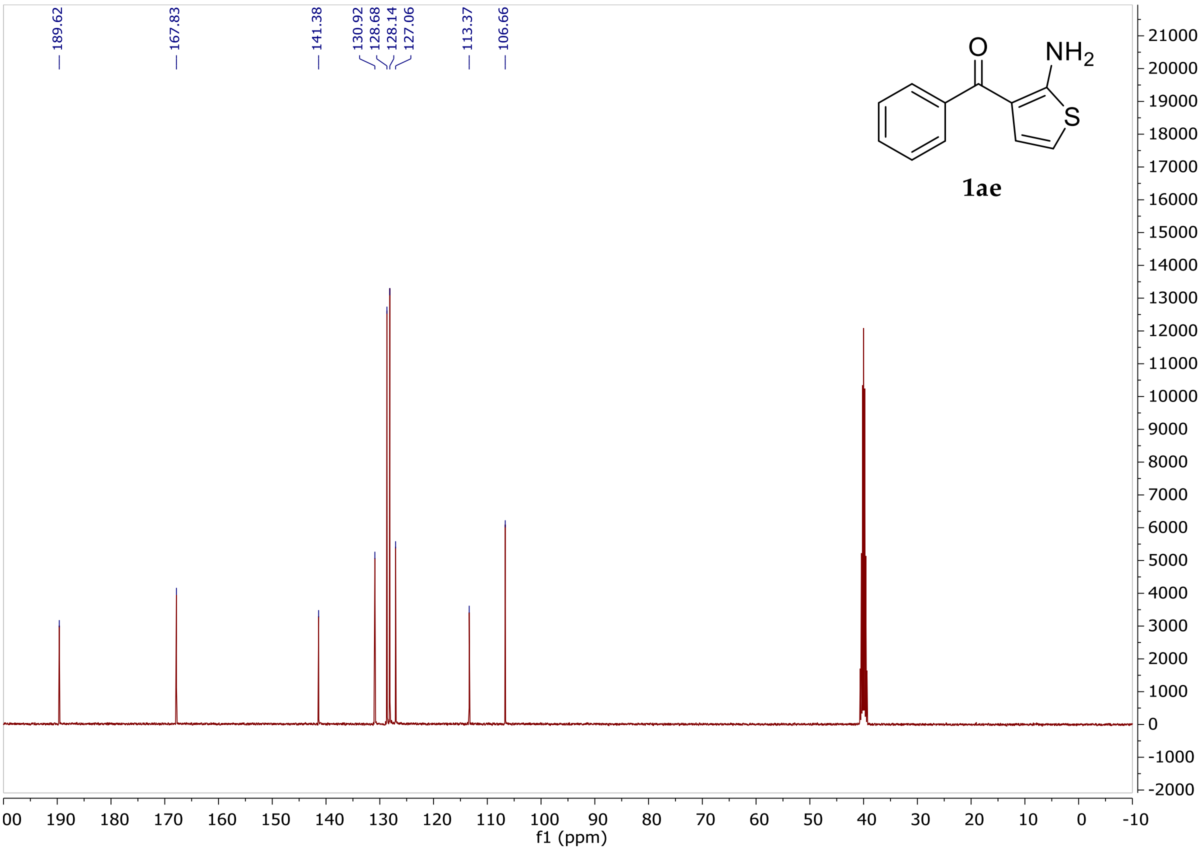
189.62
167.83
141.38
130.92
128.68
128.14
127.06
113.37
106.66
f1 (ppm)
NH2
O
S

1ae

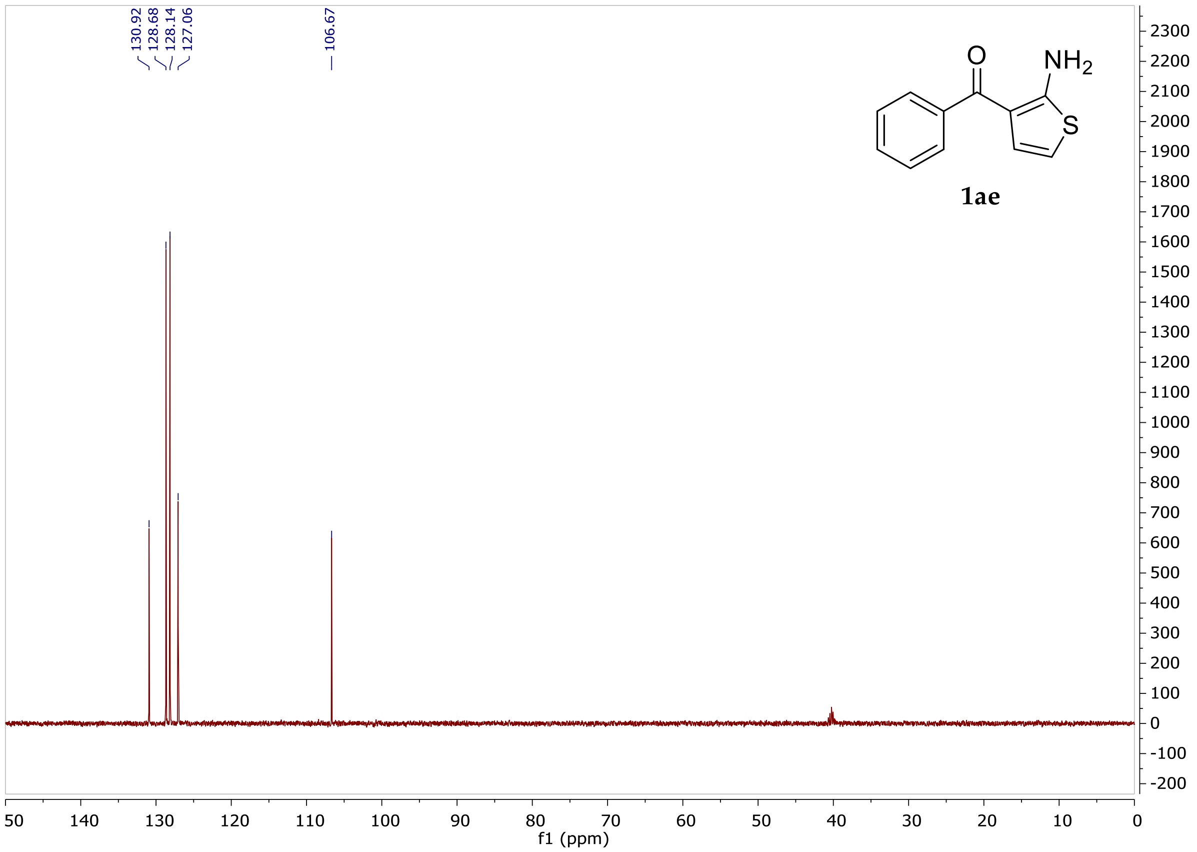
130.92
128.68
128.14
127.06
106.67
NH2
O
S
1ae
f1 (ppm)

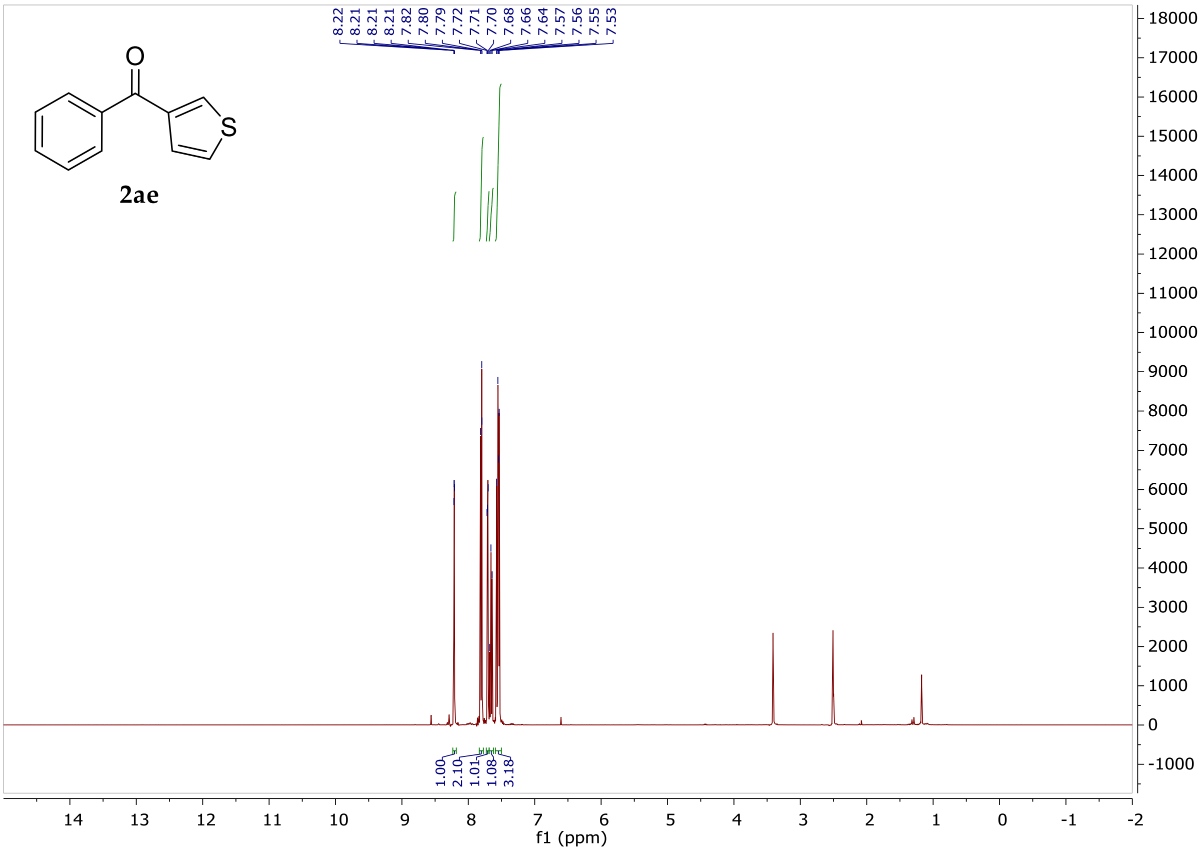
8.22
8.21
8.21
8.21
7.82
7.80
7.79
7.72
7.71
7.70
7.68
7.66
7.64
7.57
7.56
7.55
7.53
2ae
1.00
2.10
1.01
1.08
3.18
f1 (ppm)

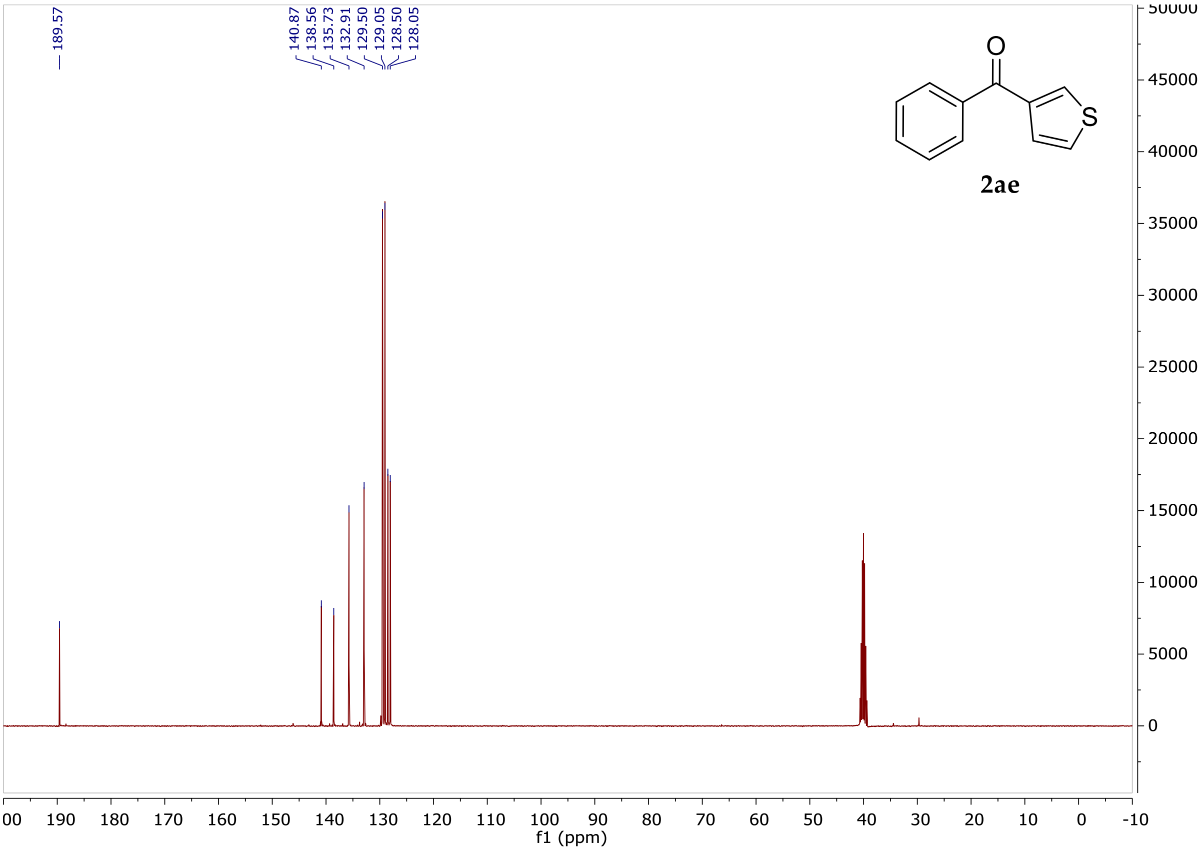
189.57
140.87
138.56
135.73
132.91
129.50
129.05
128.50
128.05
2ae
f1 (ppm)

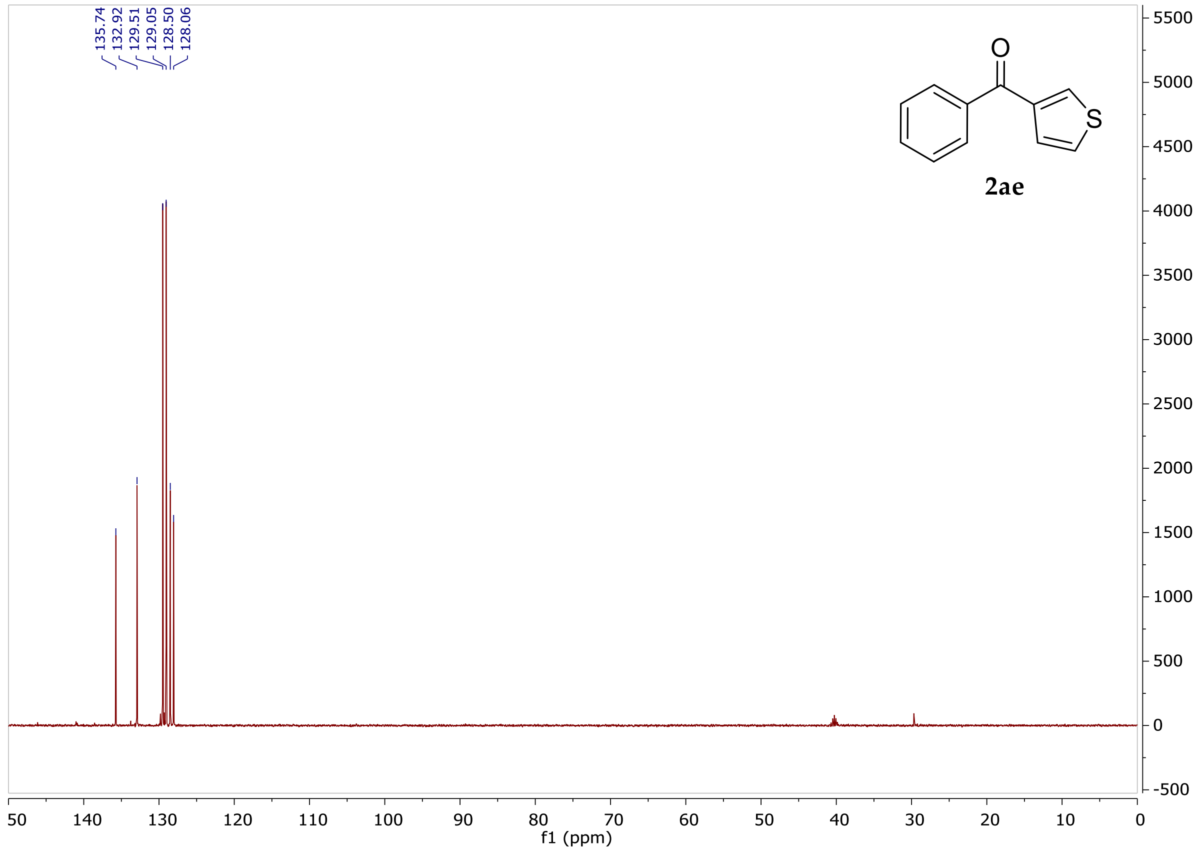

135.74
132.92
129.51
129.05
128.50
128.06
O
S
2ae
5500
5000
4500
4000
3500
3000
2500
2000
1500
1000
500
0
-500
50
140
130
120
110
100
90
80
70
60
50
40
30
20
10
0
f1 (ppm)

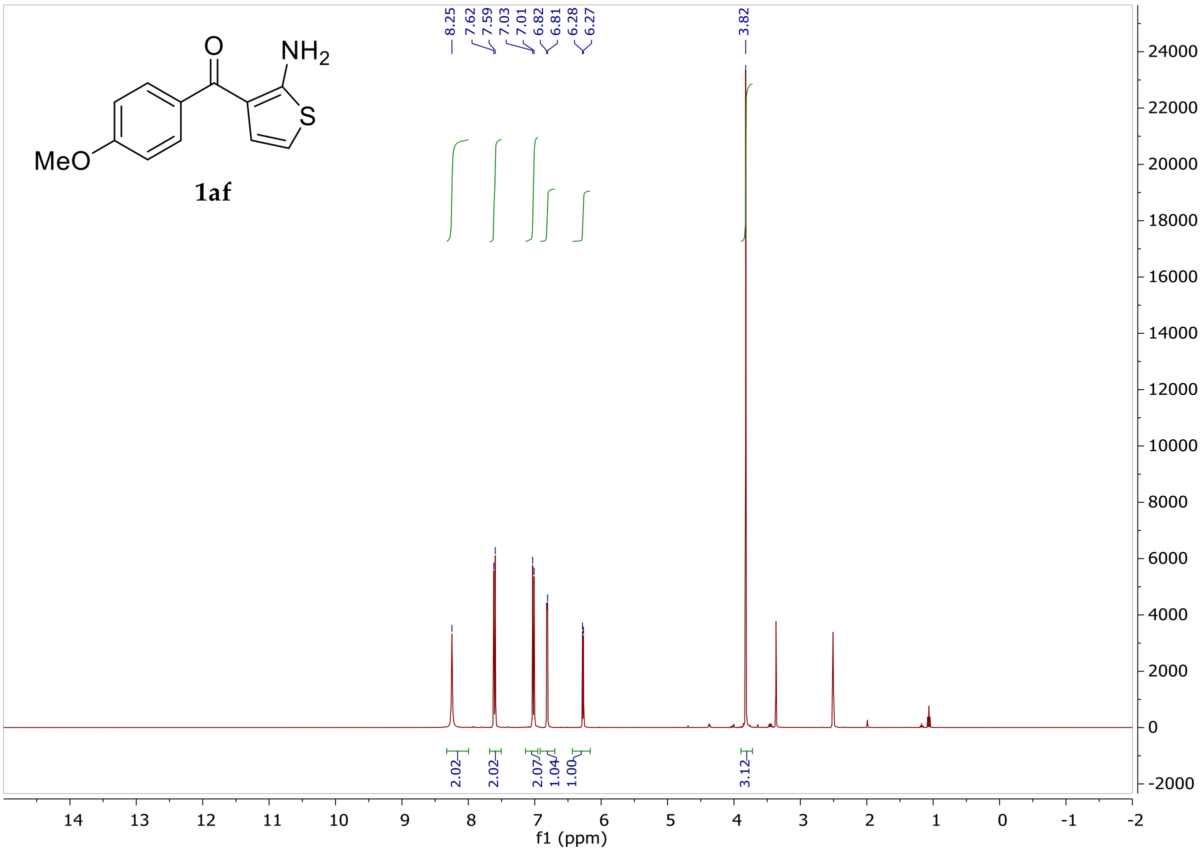

8.25
7.62
7.59
7.03
7.01
6.82
6.81
6.28
6.27
3.82
NH2
O
S
MeO
1af
2.02
2.02
2.07
1.04
1.00
3.12
f1 (ppm)

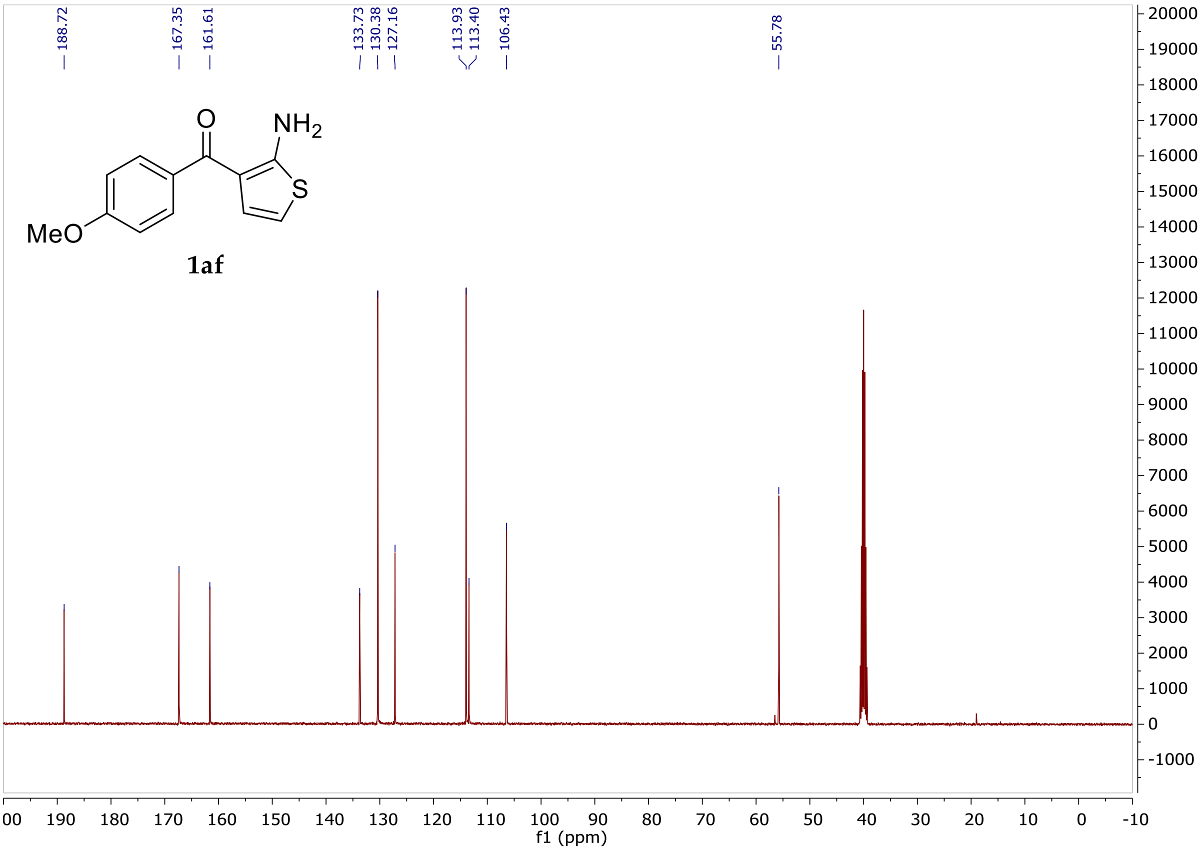

188.72
167.35
161.61
133.73
130.38
127.16
113.93
113.40
106.43
55.78
NH2
O
S
MeO
1af
f1 (ppm)

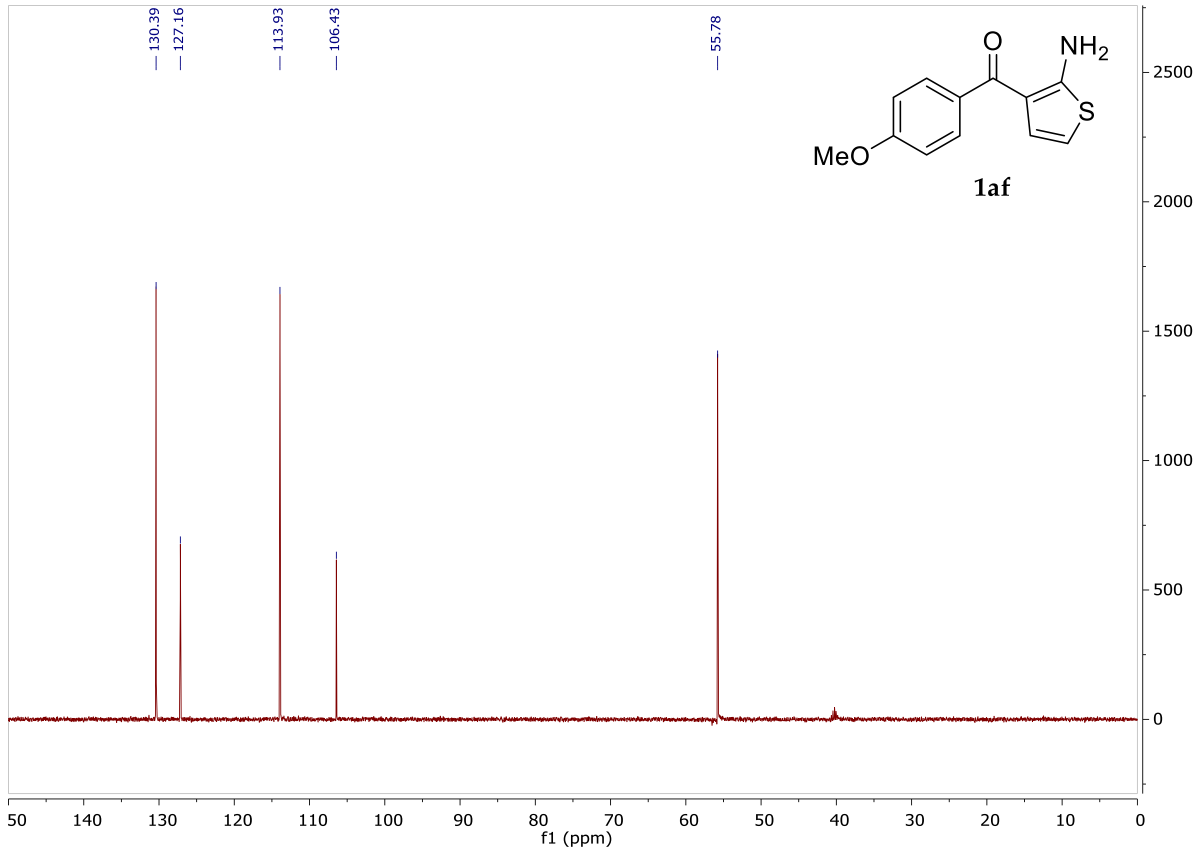

130.39
127.16
113.93
106.43
55.78
MeO
O
NH2
S
1af
f1 (ppm)

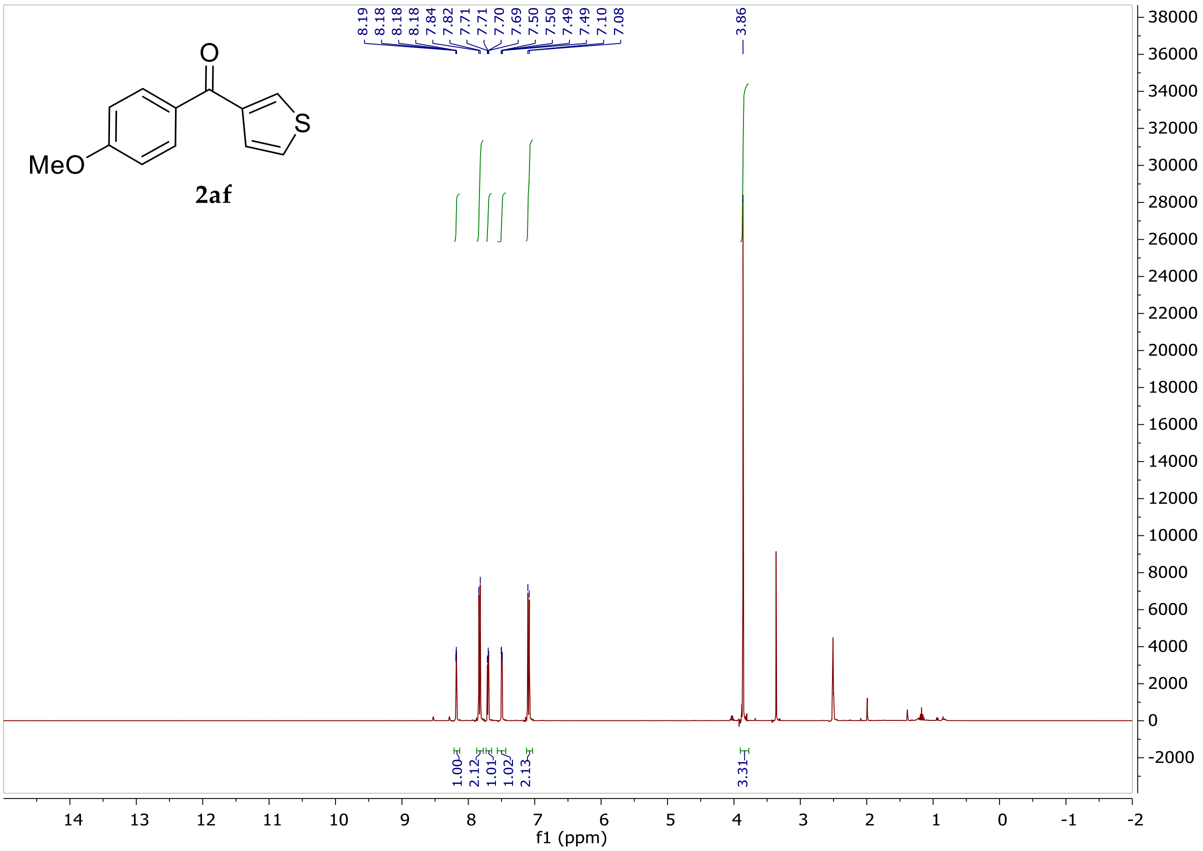
8.19
8.18
8.18
8.18
7.84
7.82
7.71
7.71
7.70
7.69
7.50
7.50
7.49
7.49
7.10
7.08
3.86
MeO
O
S
2af
1.00
2.12
1.01
1.02
2.13
3.31
f1 (ppm)

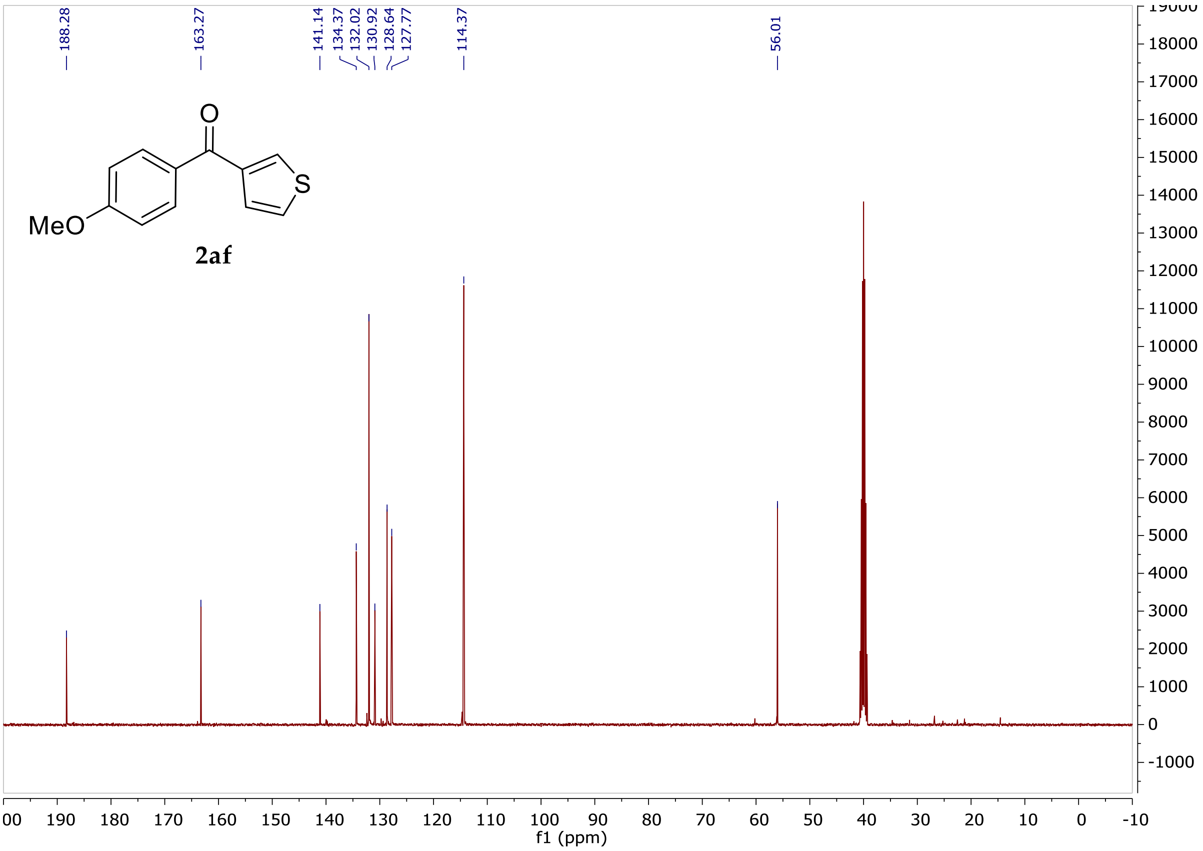
188.28
163.27
141.14
134.37
132.02
130.92
128.64
127.77
114.37
56.01
MeO
O
S
2af
f1 (ppm)

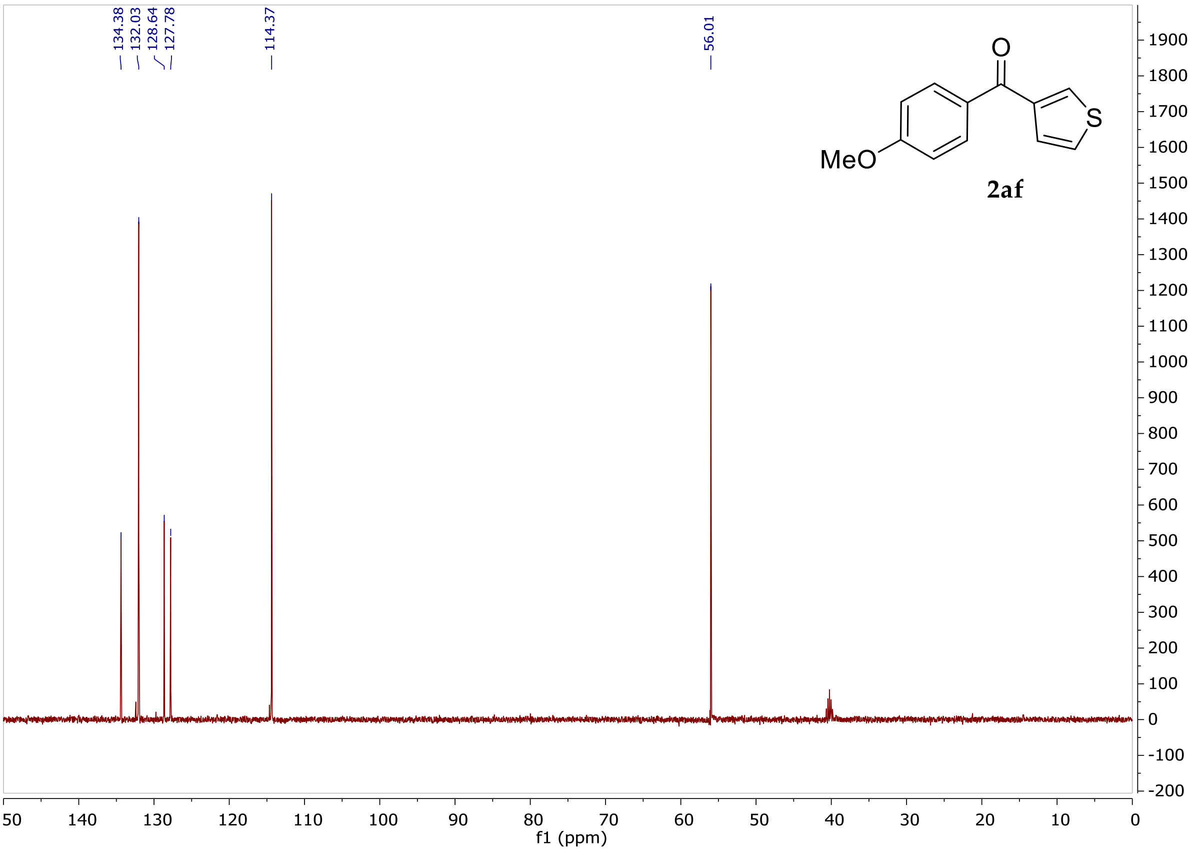
134.38
132.03
128.64
127.78
114.37
56.01
MeO
O
S
2af
1900
1800
1700
1600
1500
1400
1300
1200
1100
1000
900
800
700
600
500
400
300
200
100
0
-100
-200
50
140
130
120
110
100
90
80
70
60
50
40
30
20
10
0
f1 (ppm)

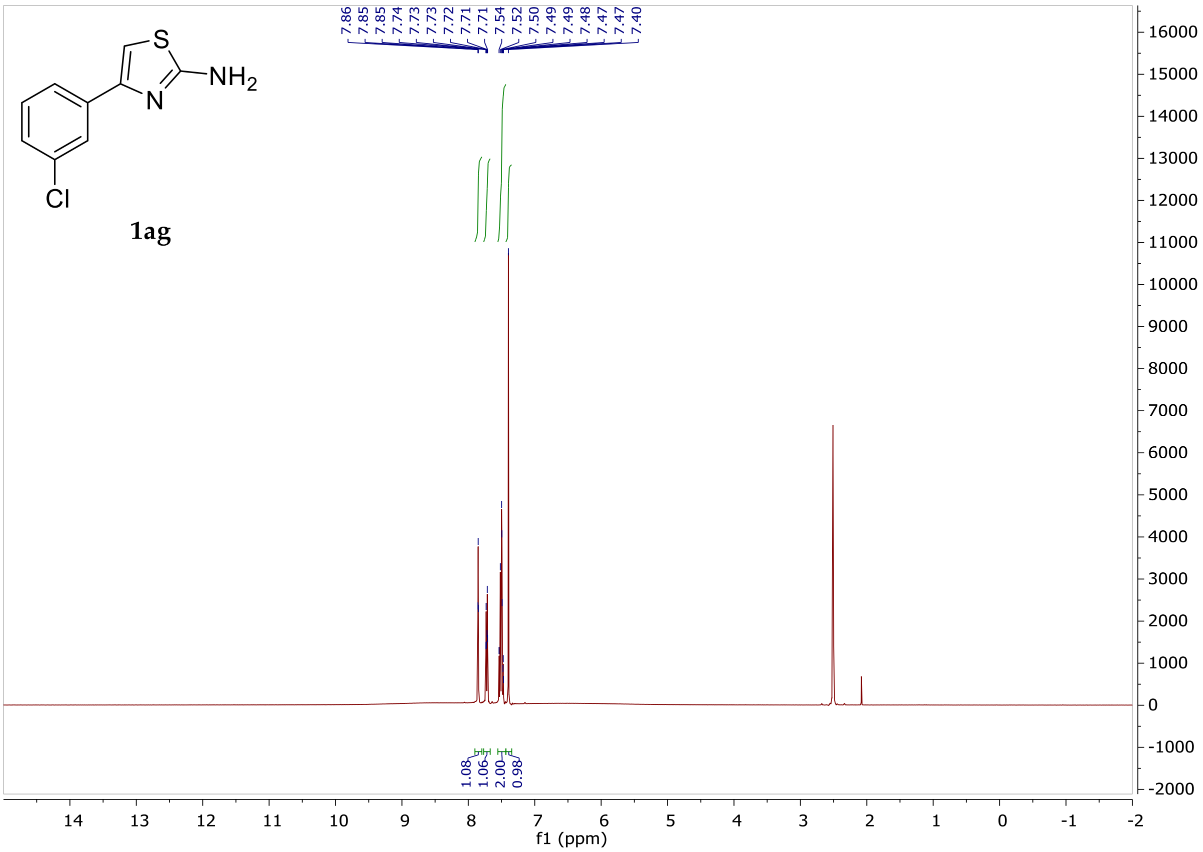
7.86
7.85
7.85
7.74
7.73
7.73
7.72
7.71
7.71
7.54
7.52
7.50
7.49
7.49
7.48
7.47
7.47
7.40
NH2
Cl
1ag
1.08
1.06
2.00
0.98
f1 (ppm)

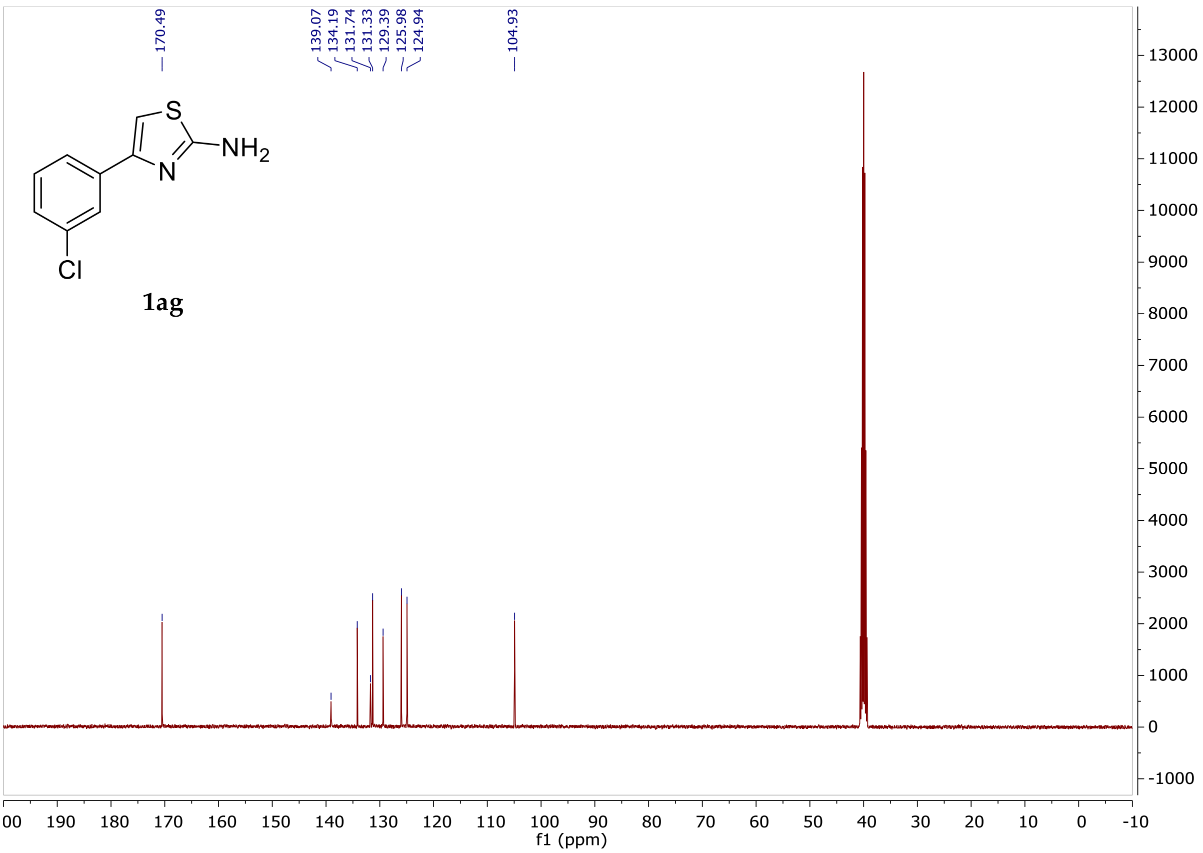
170.49
139.07
134.19
131.74
131.33
129.39
125.98
124.94
104.93
NH2
Cl
1ag
f1 (ppm)

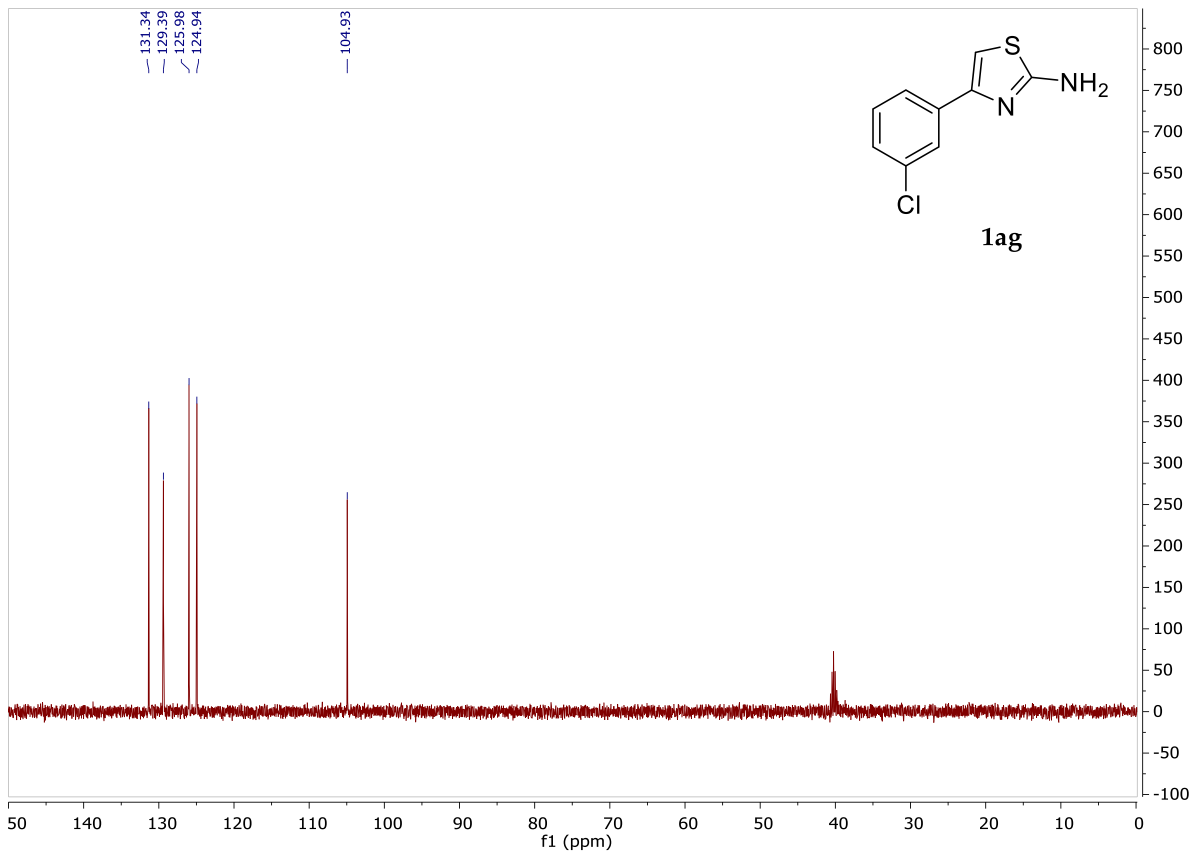

131.34
129.39
125.98
124.94
104.93
S
NH2
N
Cl
1ag
800
750
700
650
600
550
500
450
400
350
300
250
200
150
100
50
0
-50
-100
50
140
130
120
110
100
90
80
70
60
50
40
30
20
10
0
f1 (ppm)

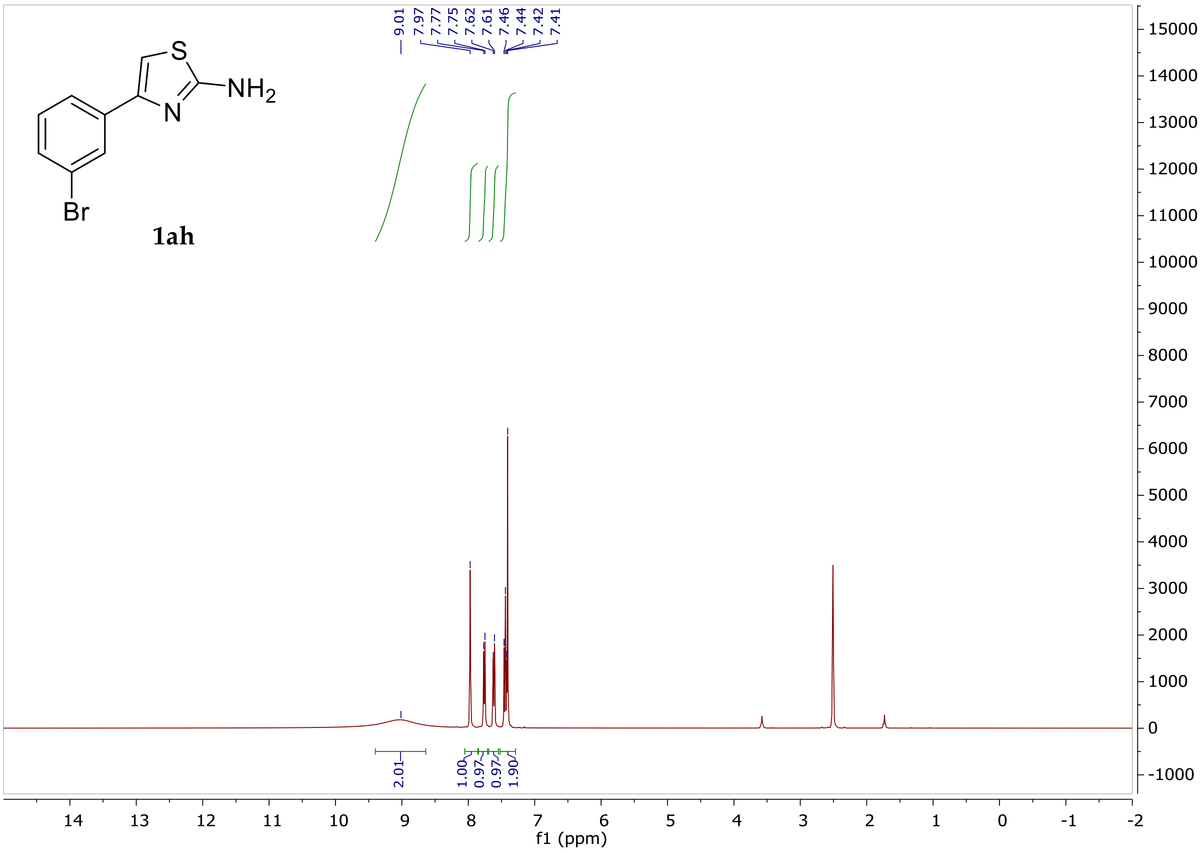
S
NH2
N
Br
1ah
9.01
7.97
7.77
7.75
7.62
7.61
7.46
7.44
7.42
7.41
2.01
1.00
0.97
0.97
1.90
f1 (ppm)

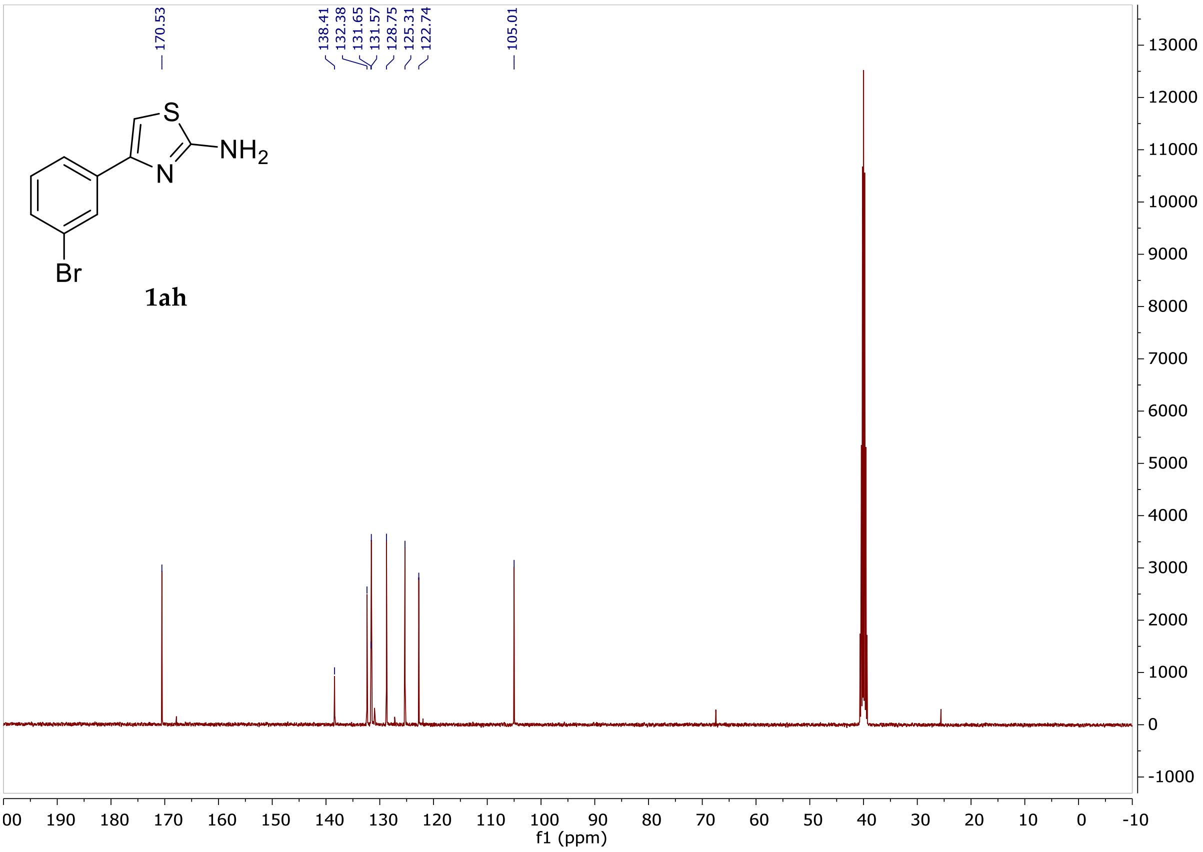
170.53
138.41
132.38
131.65
131.57
128.75
125.31
122.74
105.01
S
NH2
N
Br
1ah
f1 (ppm)

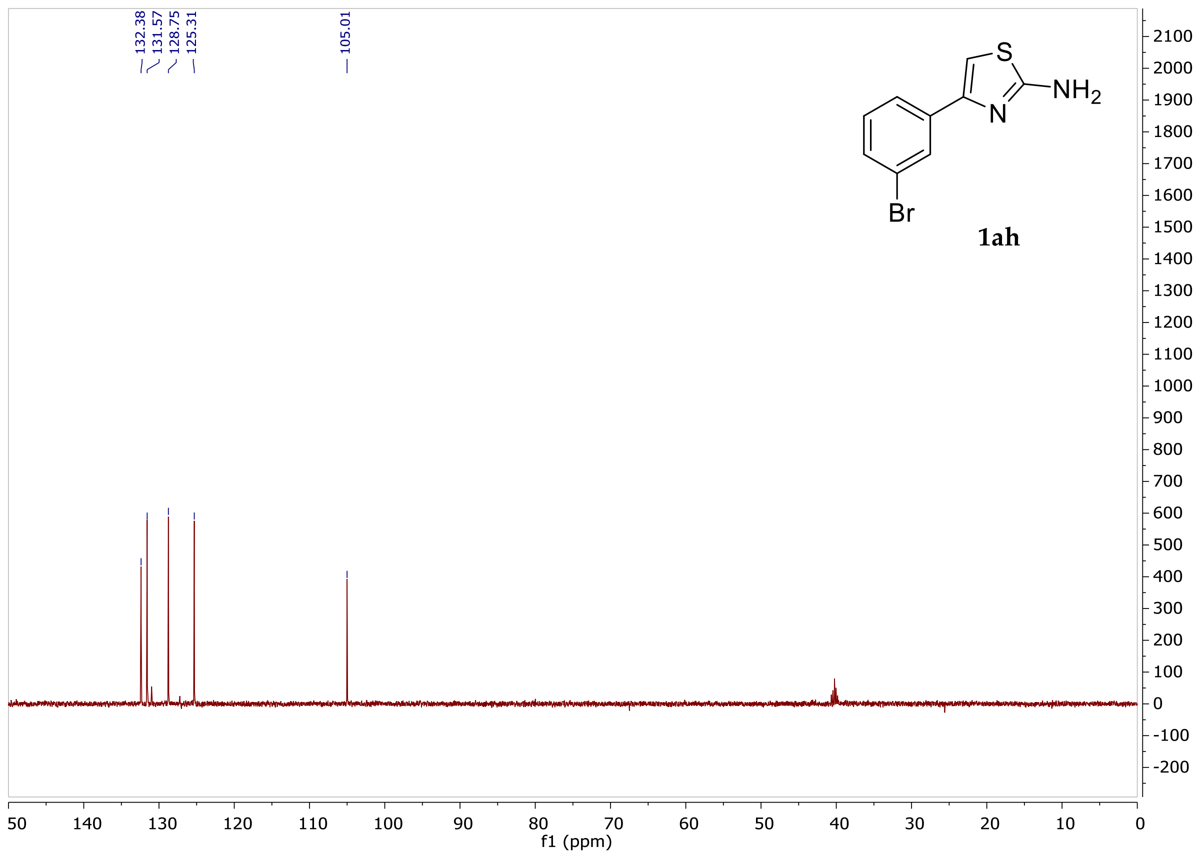
132.38
131.57
128.75
125.31
105.01
S
NH2
N
Br
1ah
f1 (ppm)

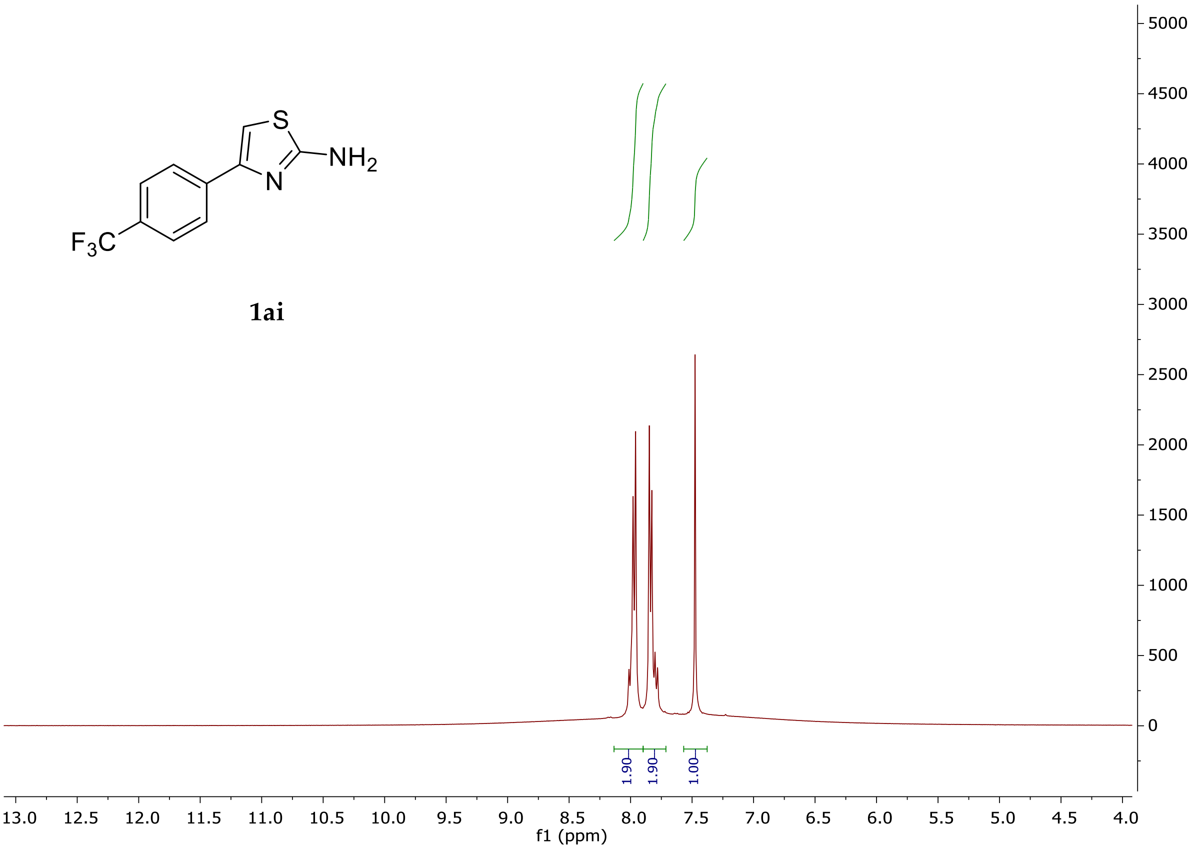

NH2
S
N
F3C
1ai
1.90
1.90
1.00
f1 (ppm)

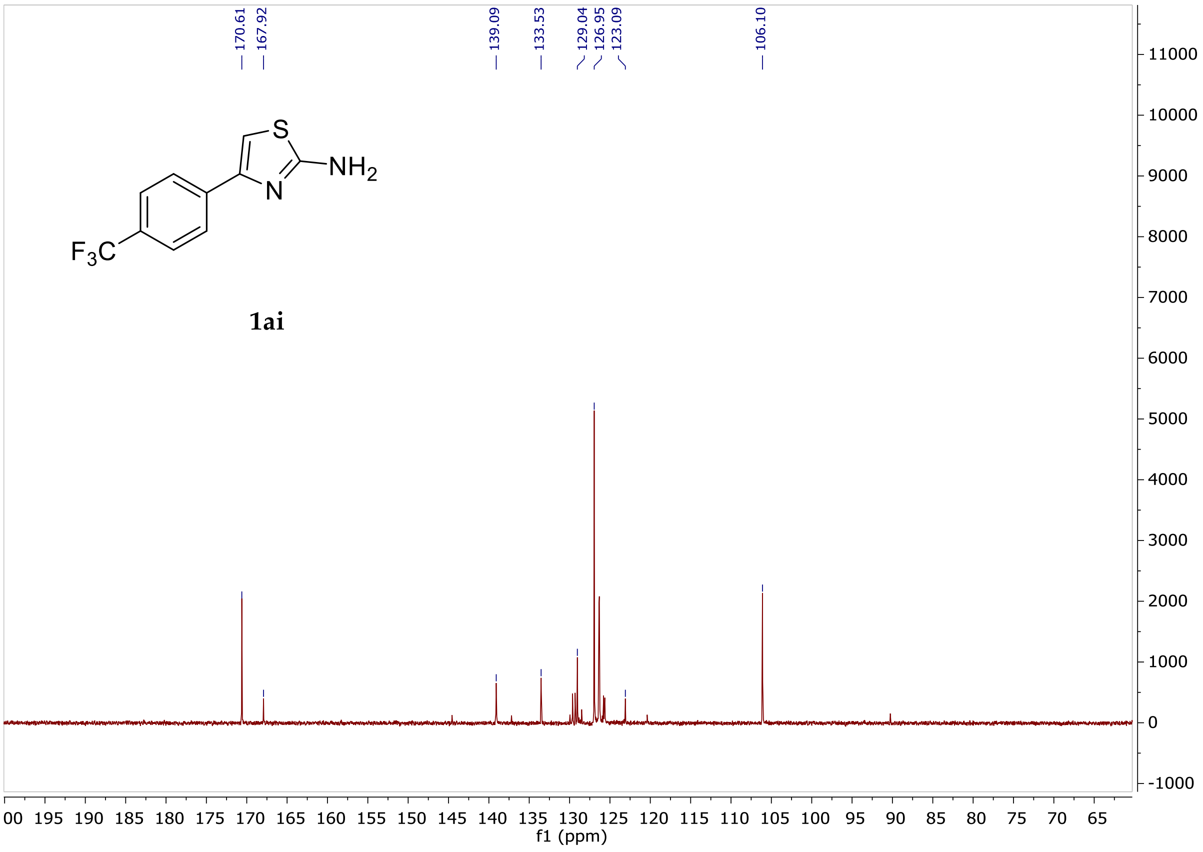

170.61
167.92
139.09
133.53
129.04
126.95
123.09
106.10
NH2
S
N
F3C
1ai
f1 (ppm)

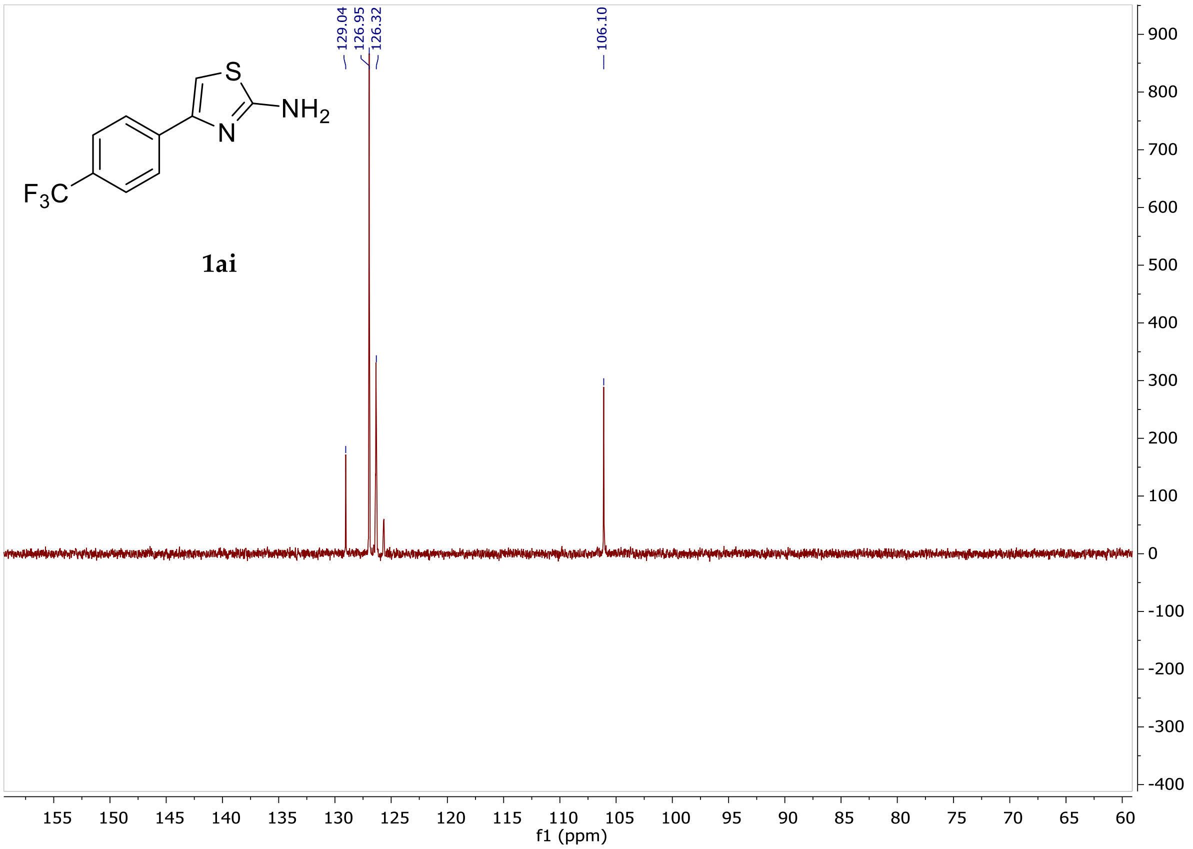

129.04
126.95
126.32
106.10
S
NH2
N
F3C
1ai
f1 (ppm)

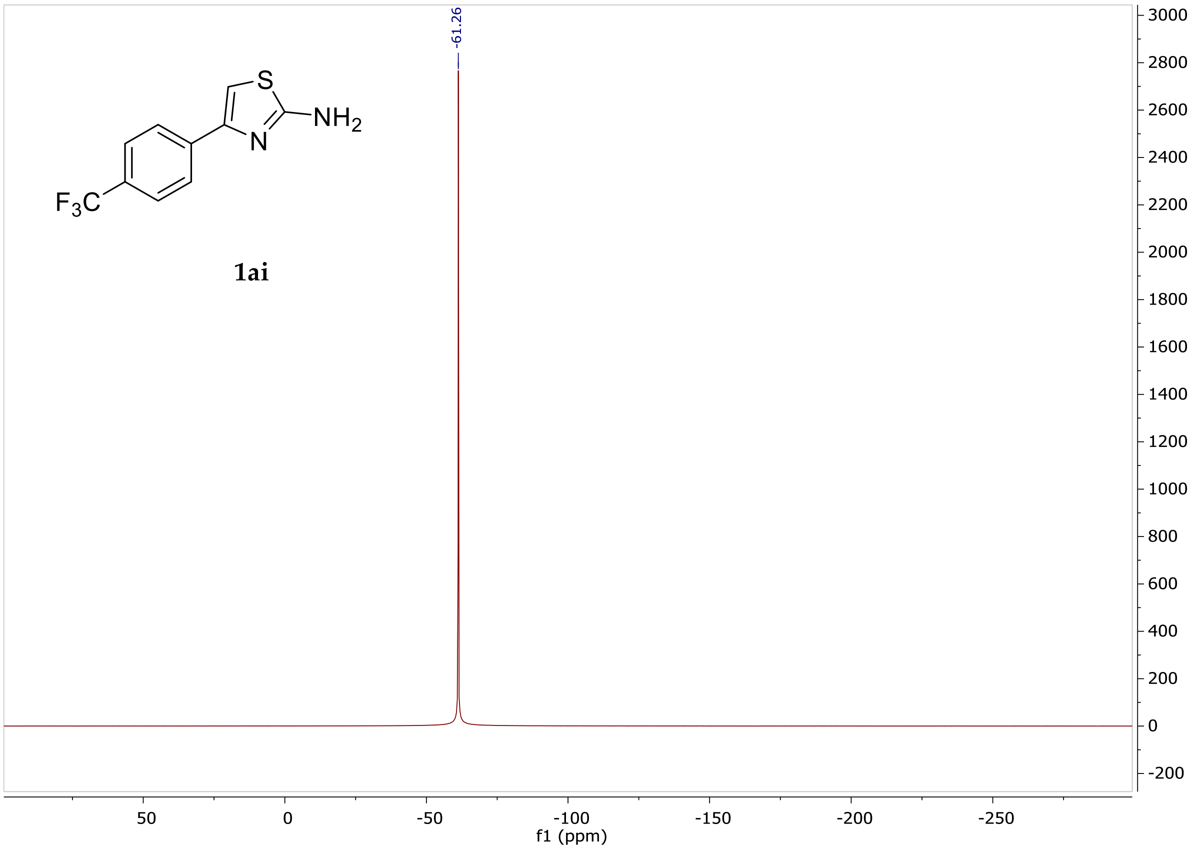

-61.26
S
NH2
N
F3C
1ai
f1 (ppm)

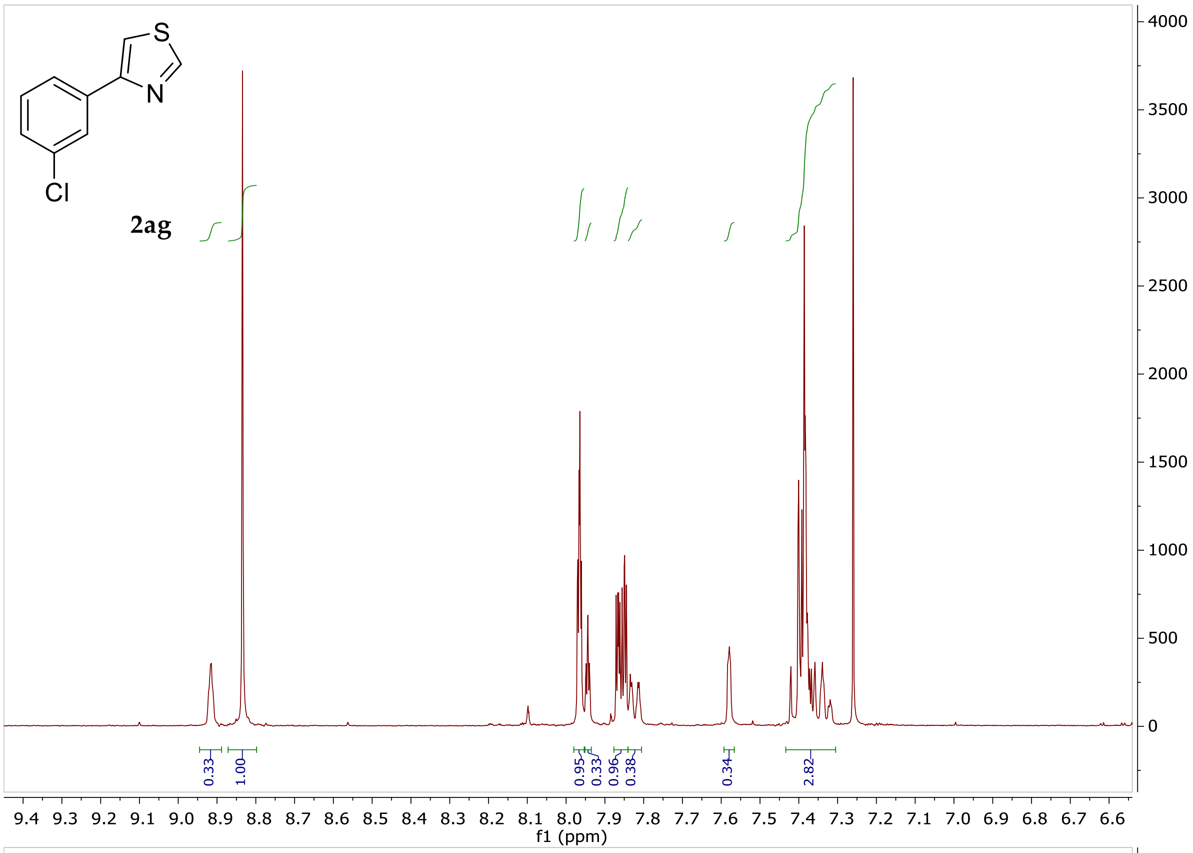
S
N
Cl
2ag
0.33
1.00
0.95
0.33
0.96
0.38
0.34
2.82
4000
3500
3000
2500
2000
1500
1000
500
0
9.4 9.3 9.2 9.1 9.0 8.9 8.8 8.7 8.6 8.5 8.4 8.3 8.2 8.1 8.0 7.9 7.8 7.7 7.6 7.5 7.4 7.3 7.2 7.1 7.0 6.9 6.8 6.7 6.6
f1 (ppm)

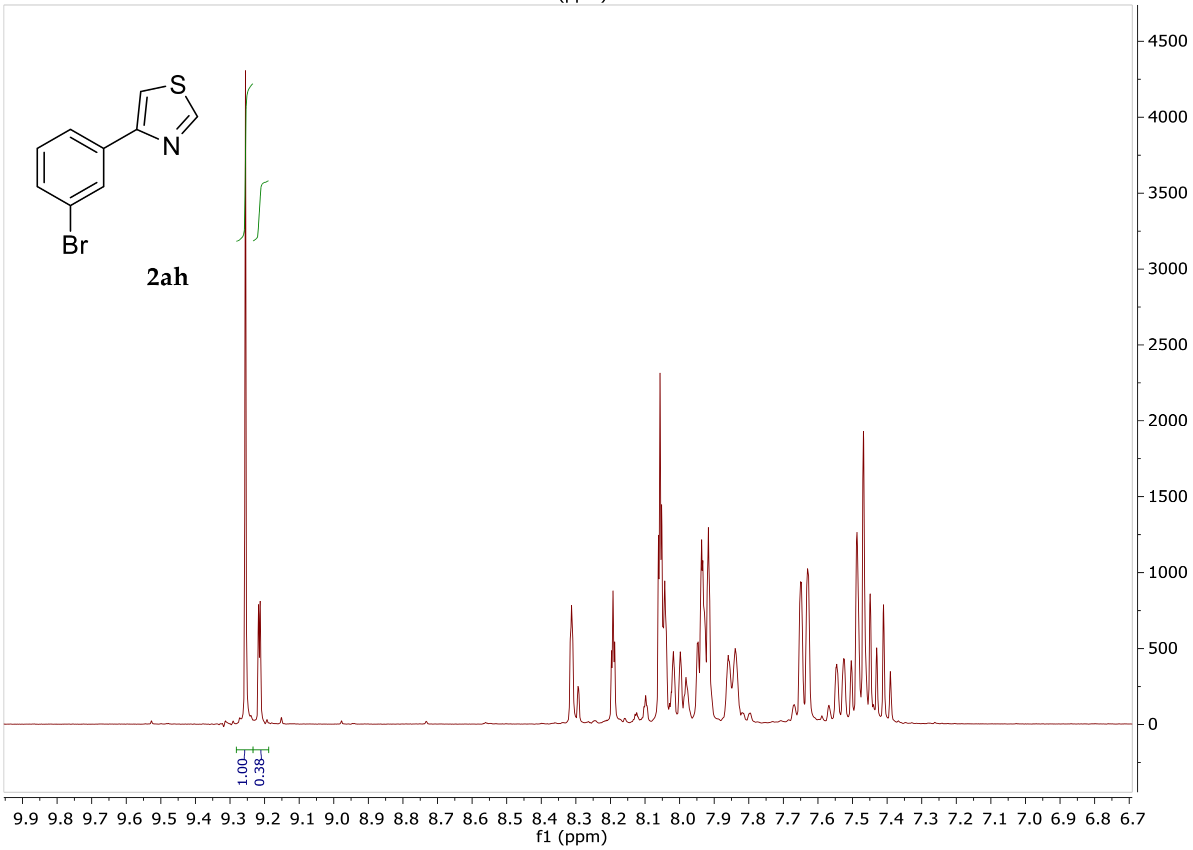
S
N
Br
2ah
1.00
0.38
4500
4000
3500
3000
2500
2000
1500
1000
500
0
9.9 9.8 9.7 9.6 9.5 9.4 9.3 9.2 9.1 9.0 8.9 8.8 8.7 8.6 8.5 8.4 8.3 8.2 8.1 8.0 7.9 7.8 7.7 7.6 7.5 7.4 7.3 7.2 7.1 7.0 6.9 6.8 6.7
f1 (ppm)

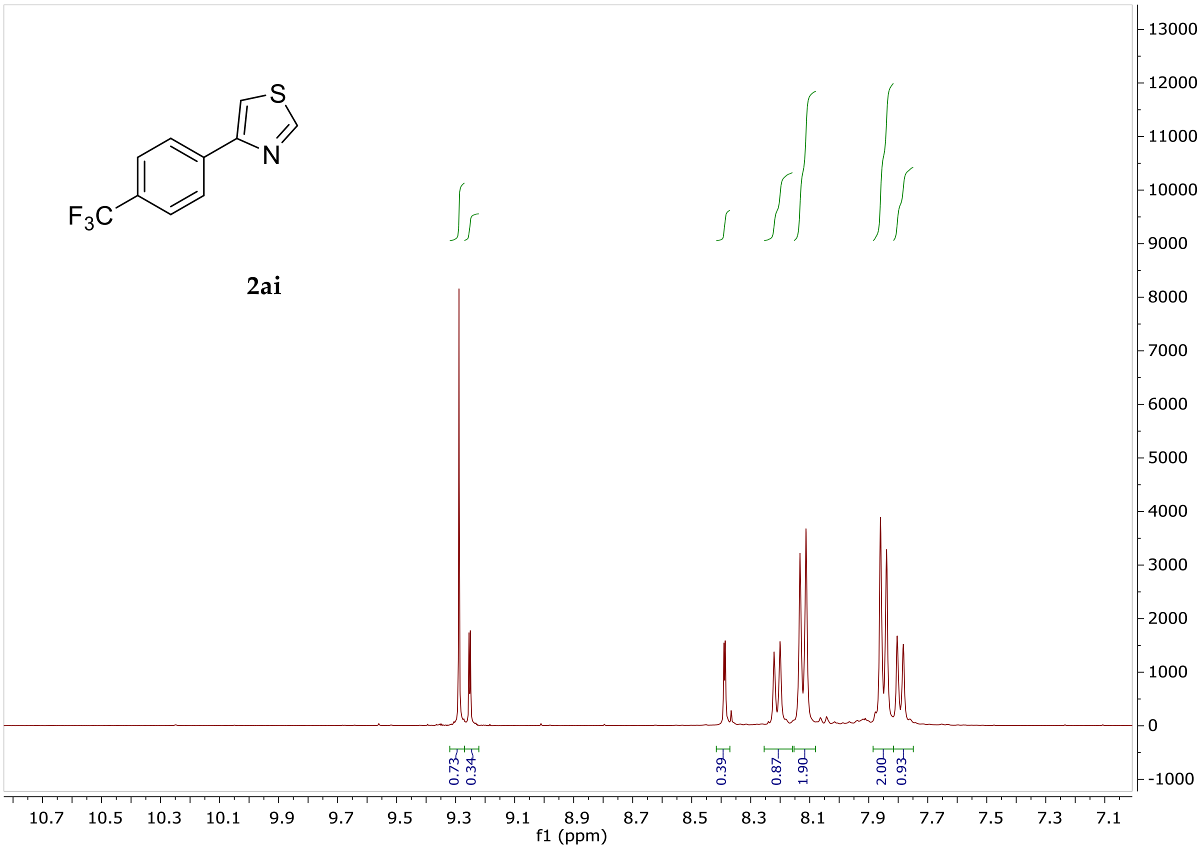
F3C
S
N
2ai
13000
12000
11000
10000
9000
8000
7000
6000
5000
4000
3000
2000
1000
0
-1000
0.73
0.34
0.39
0.87
1.90
2.00
0.93
10.7
10.5
10.3
10.1
9.9
9.7
9.5
9.3
9.1
8.9
8.7
8.5
8.3
8.1
7.9
7.7
7.5
7.3
7.1
f1 (ppm)

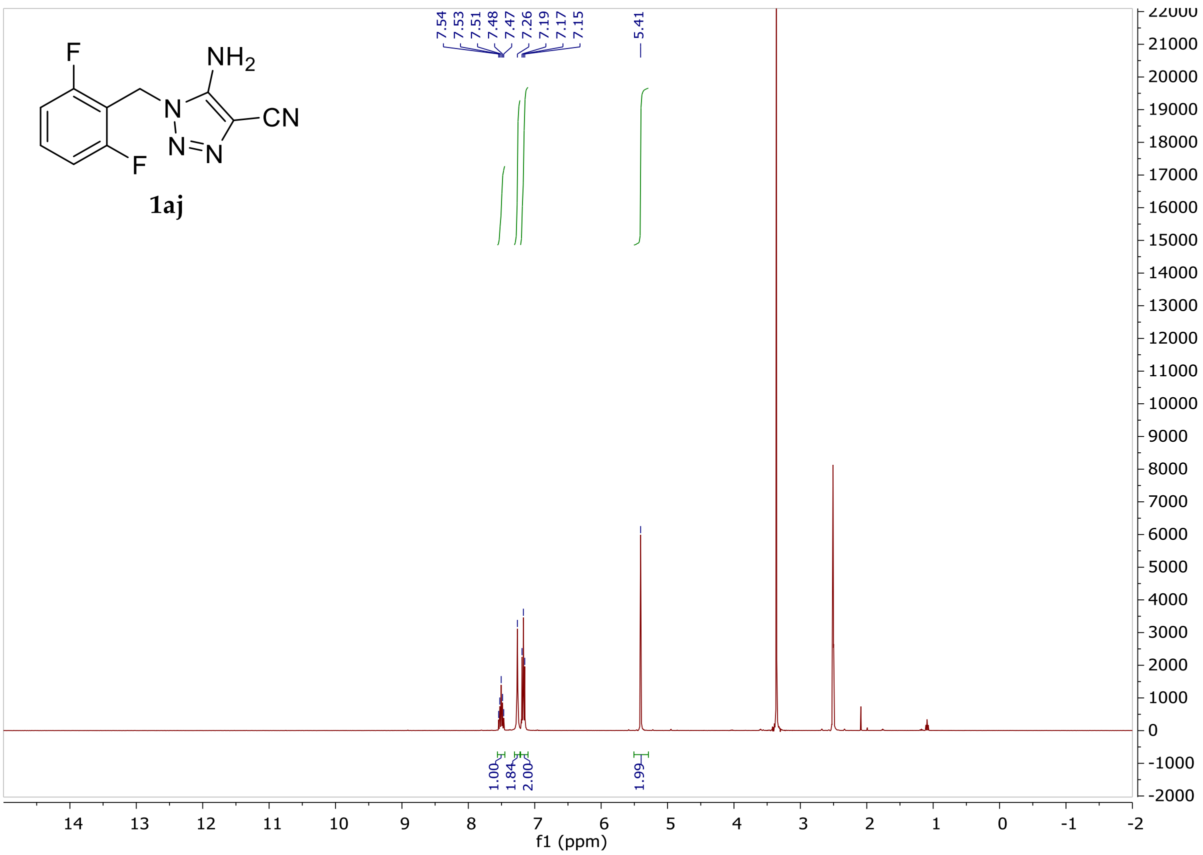

7.54
7.53
7.51
7.48
7.47
7.26
7.19
7.17
7.15
5.41
F
NH2
N
CN
N
N
F
1aj
1.00
1.84
2.00
1.99
f1 (ppm)

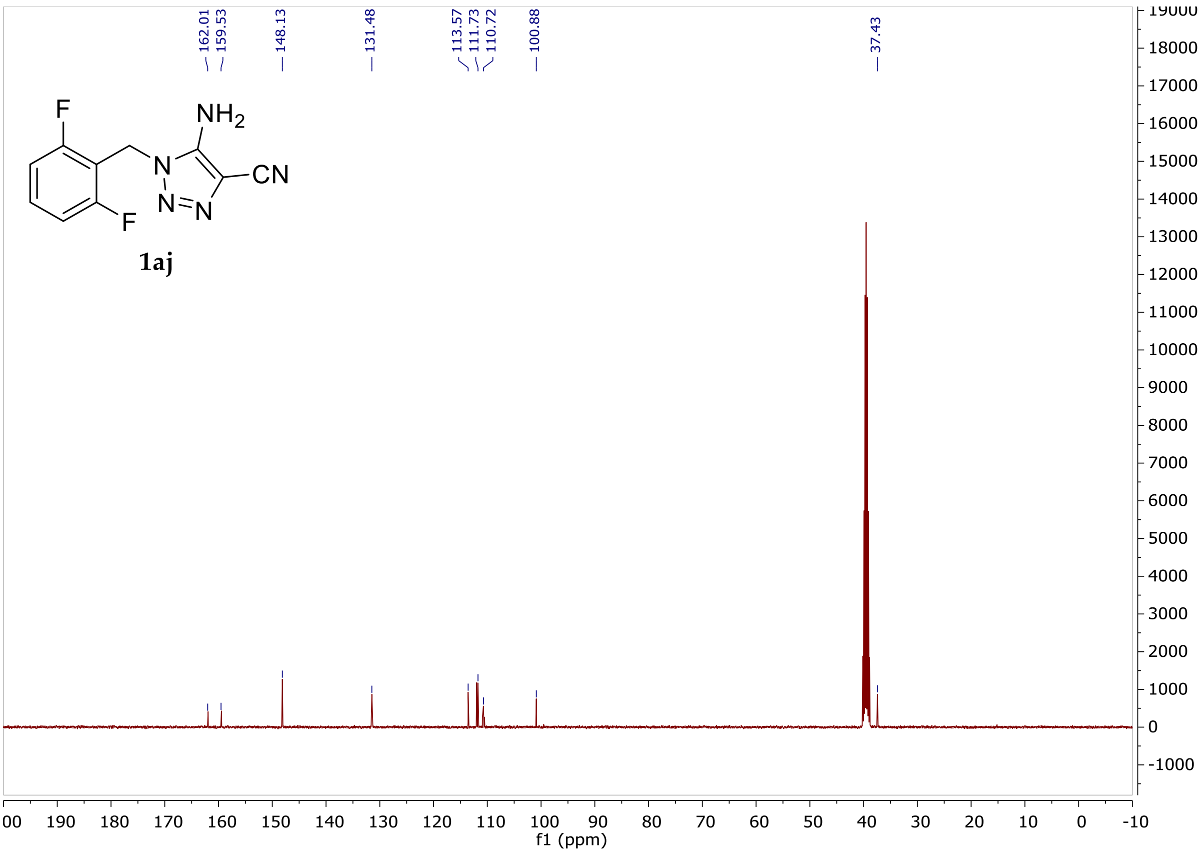

162.01
159.53
148.13
131.48
113.57
111.73
110.72
100.88
37.43
F
NH2
N
CN
N
N
F
1aj
f1 (ppm)

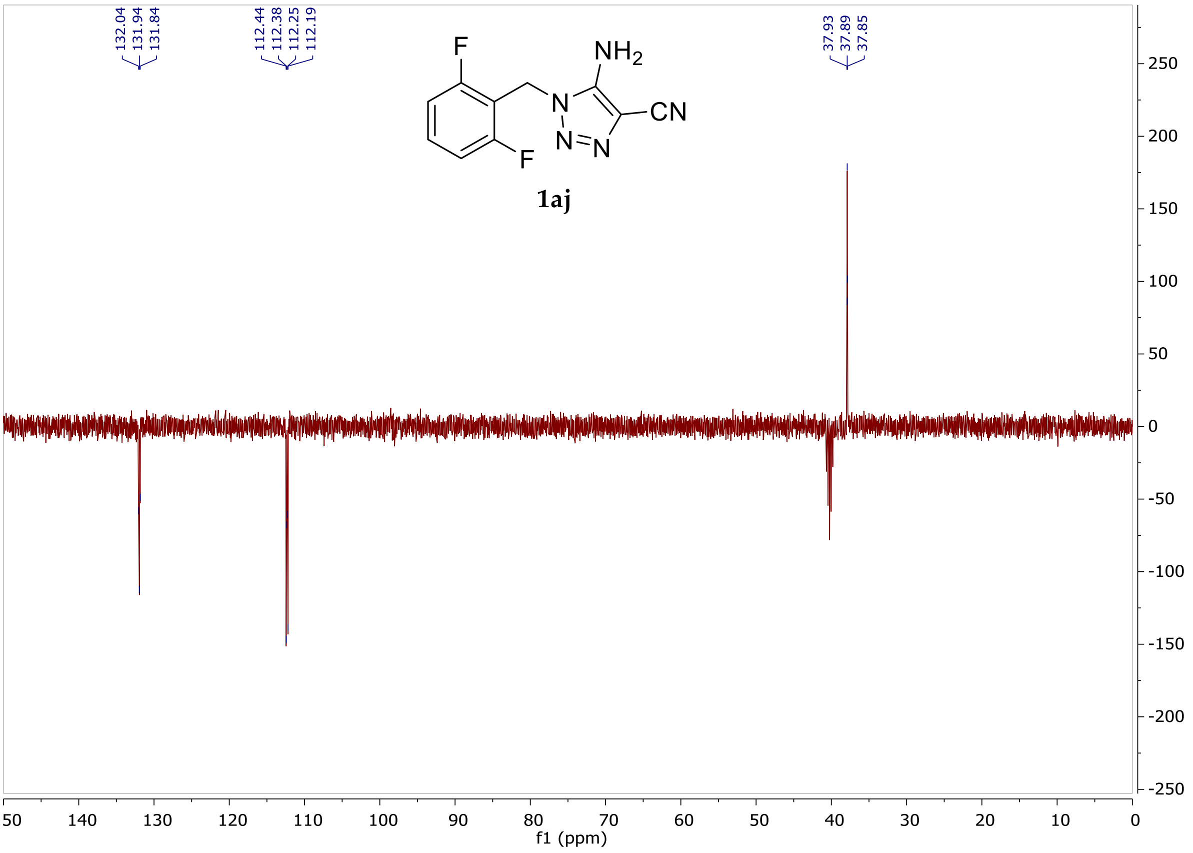
132.04
131.94
131.84
112.44
112.38
112.25
112.19
37.93
37.89
37.85
F
NH2
CN
N
N
N
F
1aj
250
200
150
100
50
0
-50
-100
-150
-200
-250
50
140
130
120
110
100
90
80
70
60
50
40
30
20
10
0
f1 (ppm)

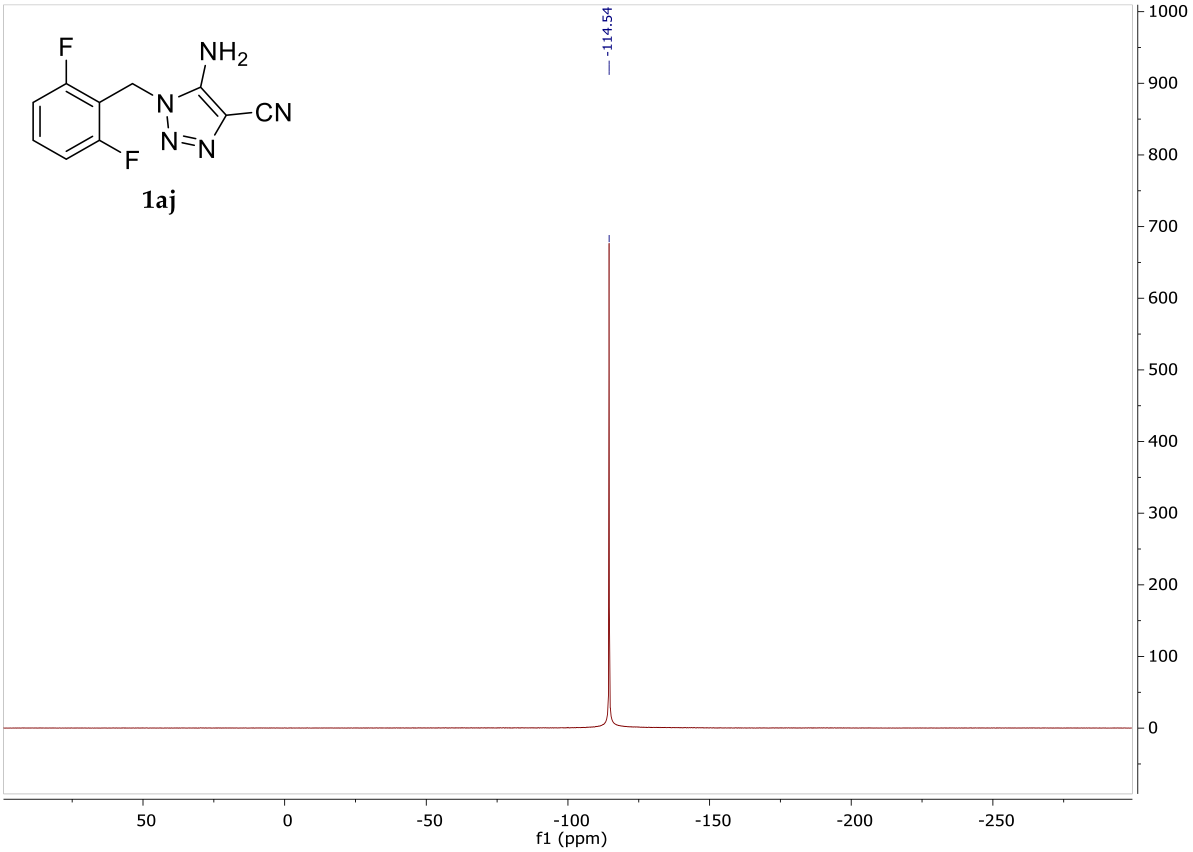
-114.54
F
NH2
CN
N
N
N
F
1aj
1000
900
800
700
600
500
400
300
200
100
0
50
0
-50
-100
-150
-200
-250
f1 (ppm)

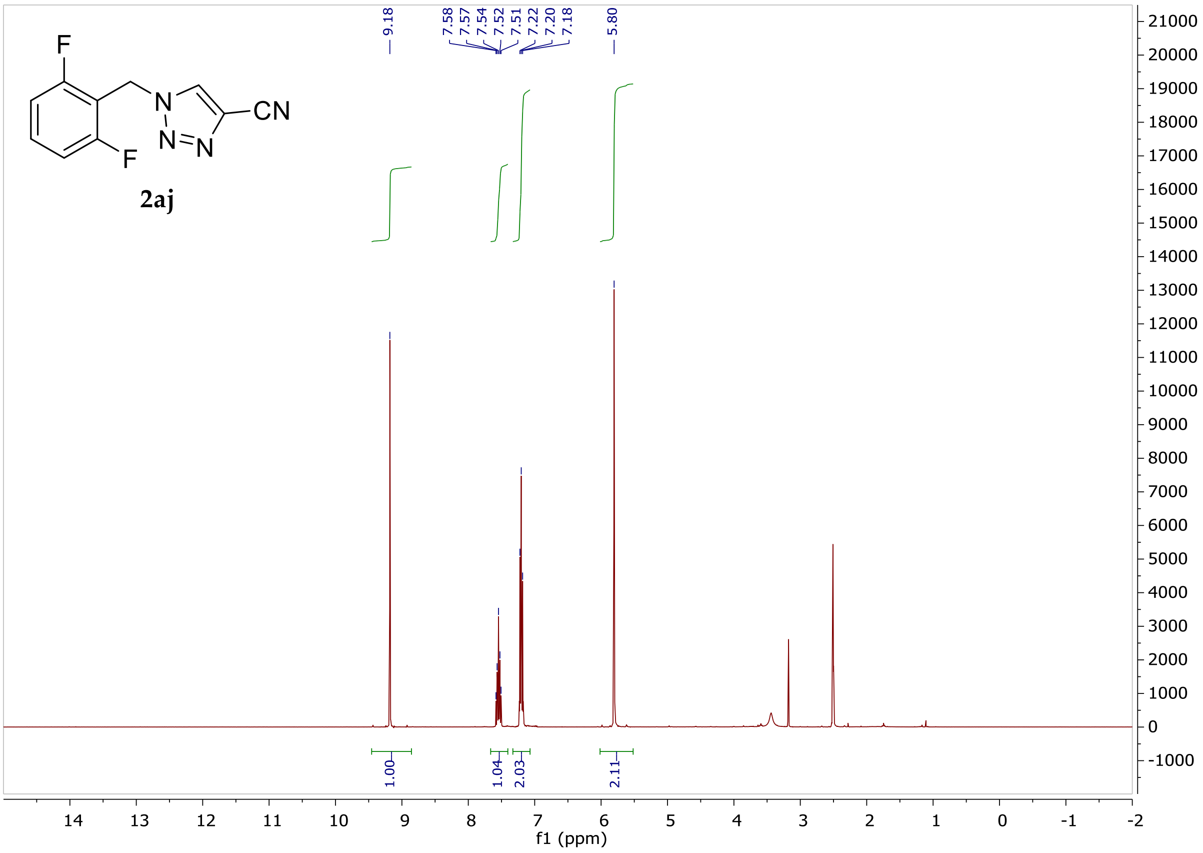

2aj
CN
F
N
9.18
7.58
7.57
7.54
7.52
7.51
7.22
7.20
7.18
5.80
1.00
1.04
2.03
2.11
f1 (ppm)

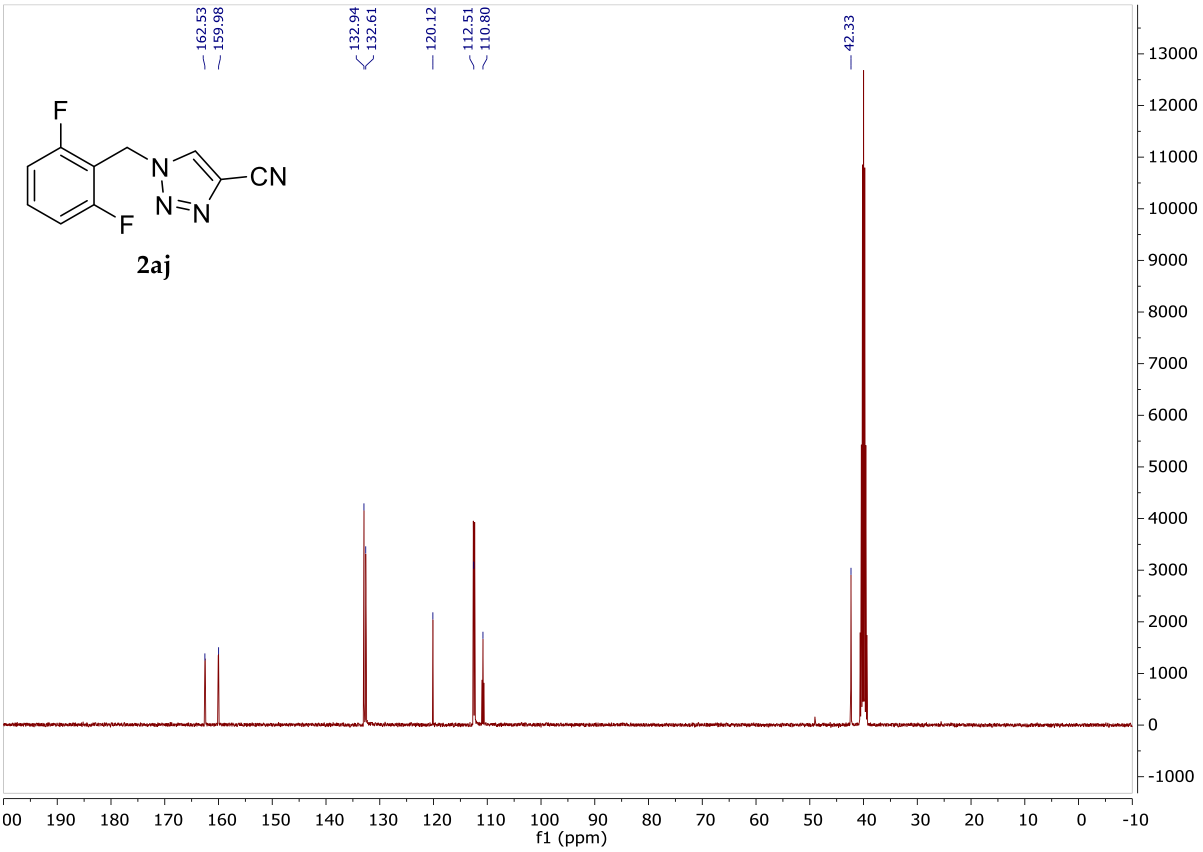

2aj
CN
F
N
162.53
159.98
132.94
132.61
120.12
112.51
110.80
42.33
f1 (ppm)

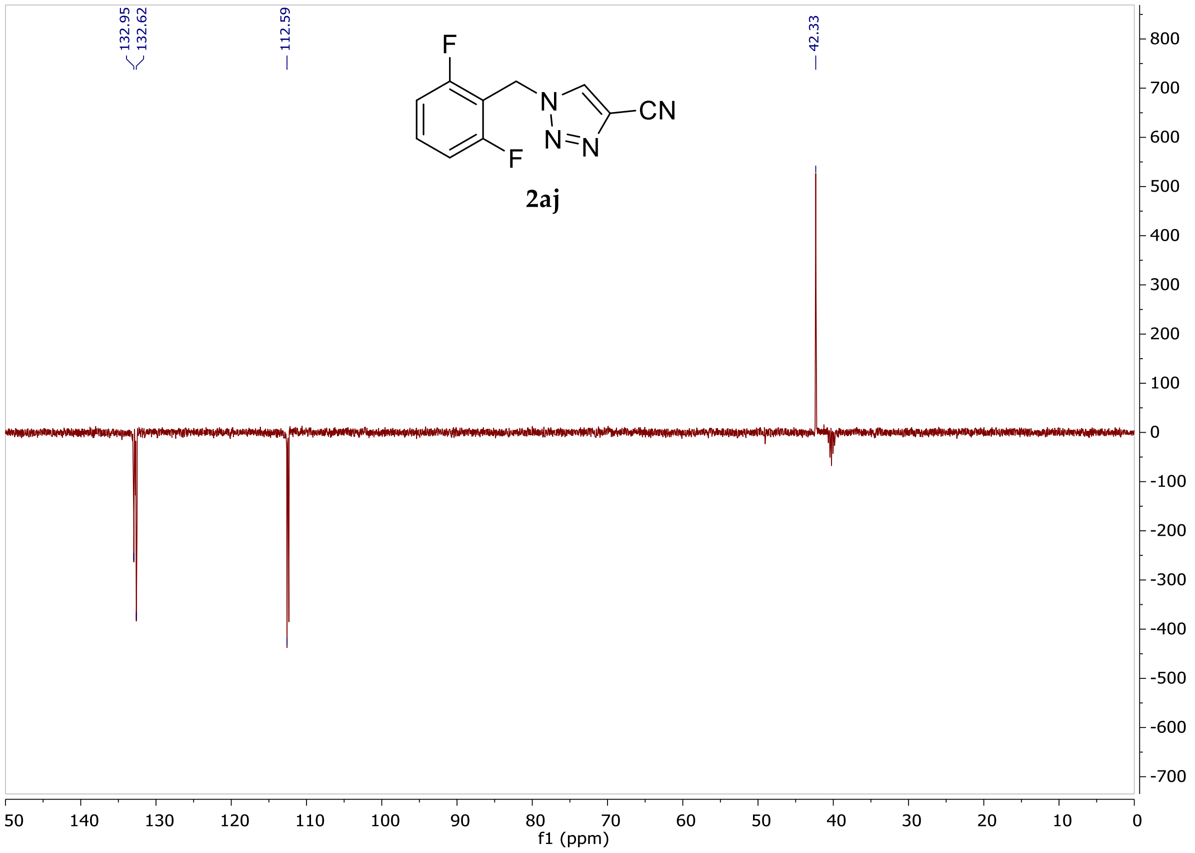
132.95
132.62
112.59
42.33
F
N
CN
N
N
F
2aj
f1 (ppm)

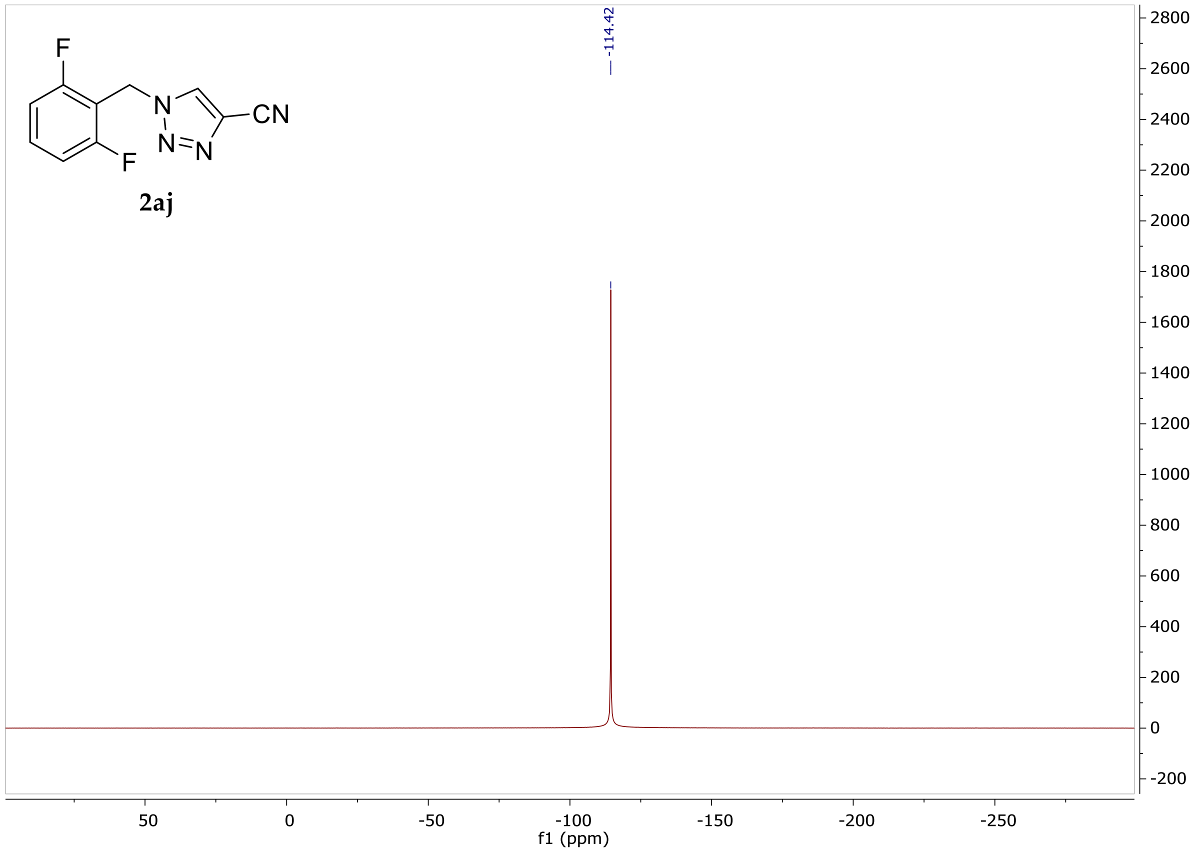
-114.42
F
N
CN
N
N
F
2aj
f1 (ppm)

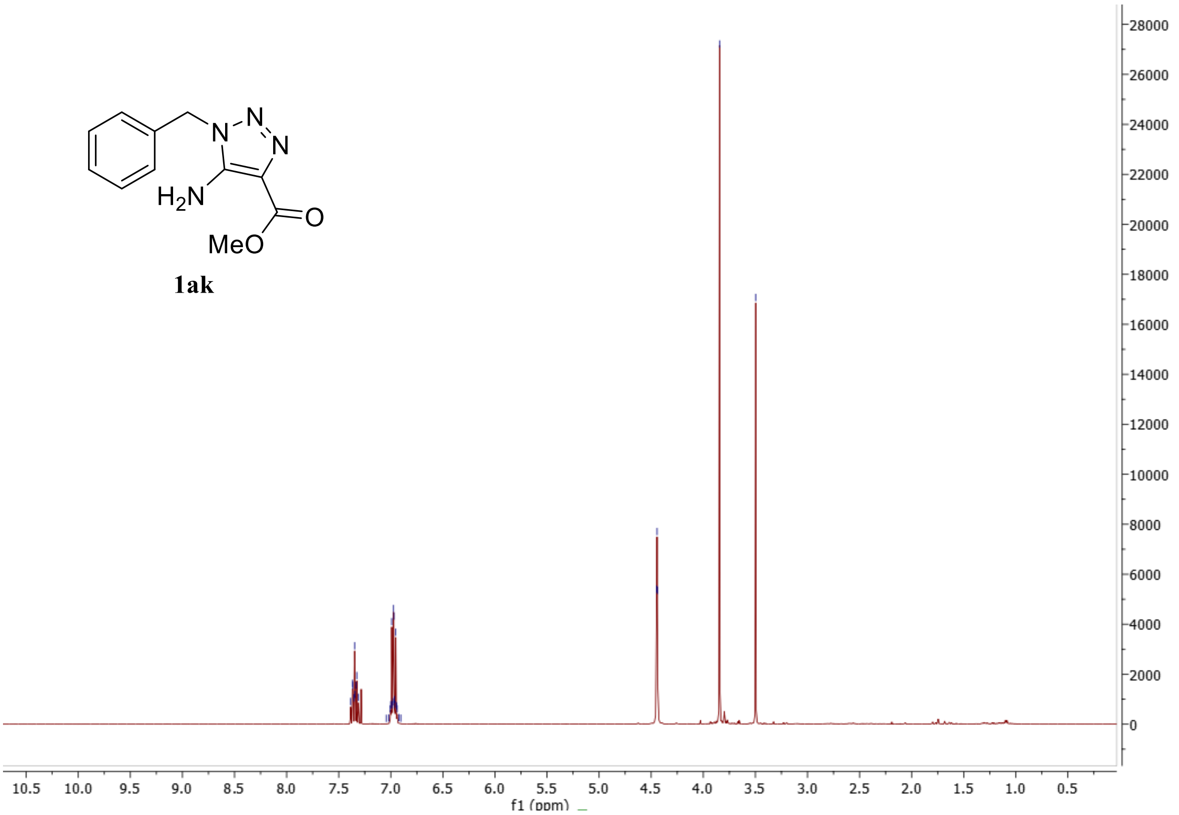
1ak
f1 (ppm)

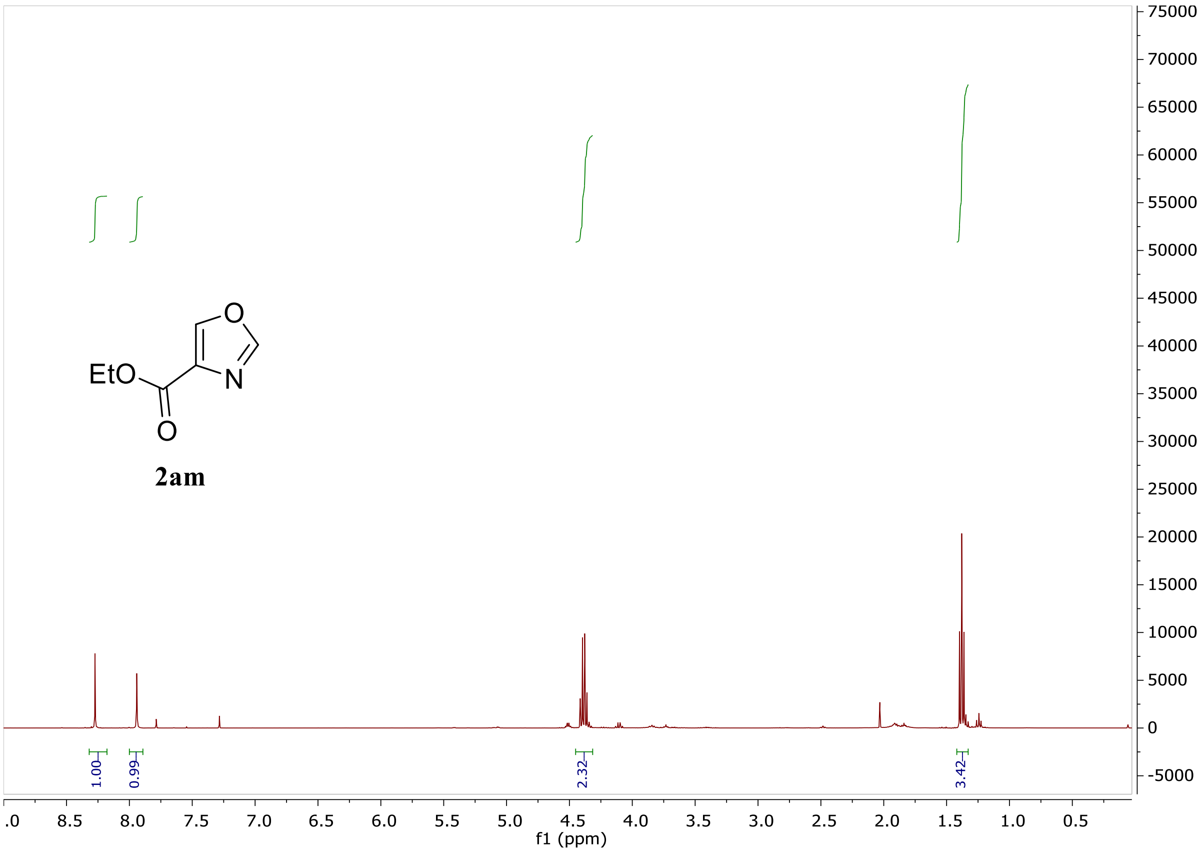
2am
f1 (ppm)

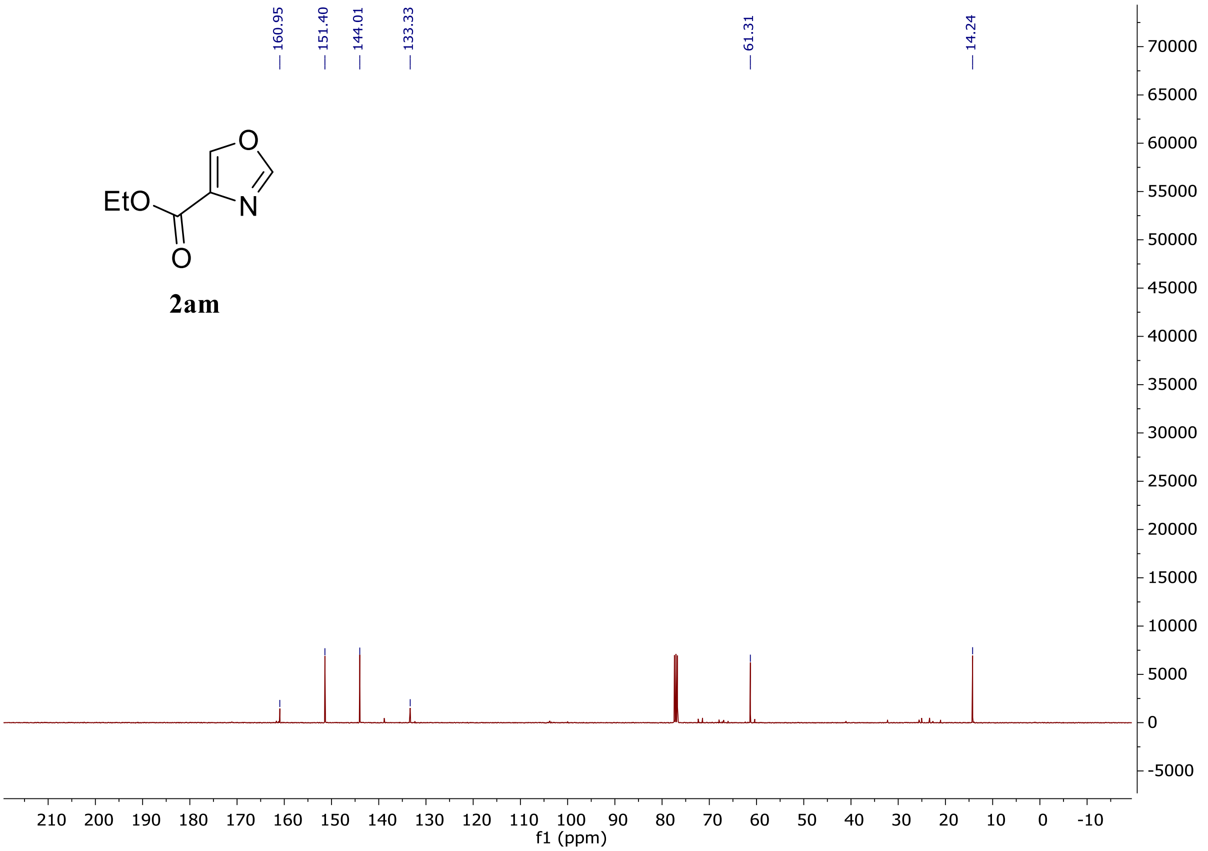
160.95
151.40
144.01
133.33
61.31
14.24
EtO
O
N
O
f1 (ppm)

2am

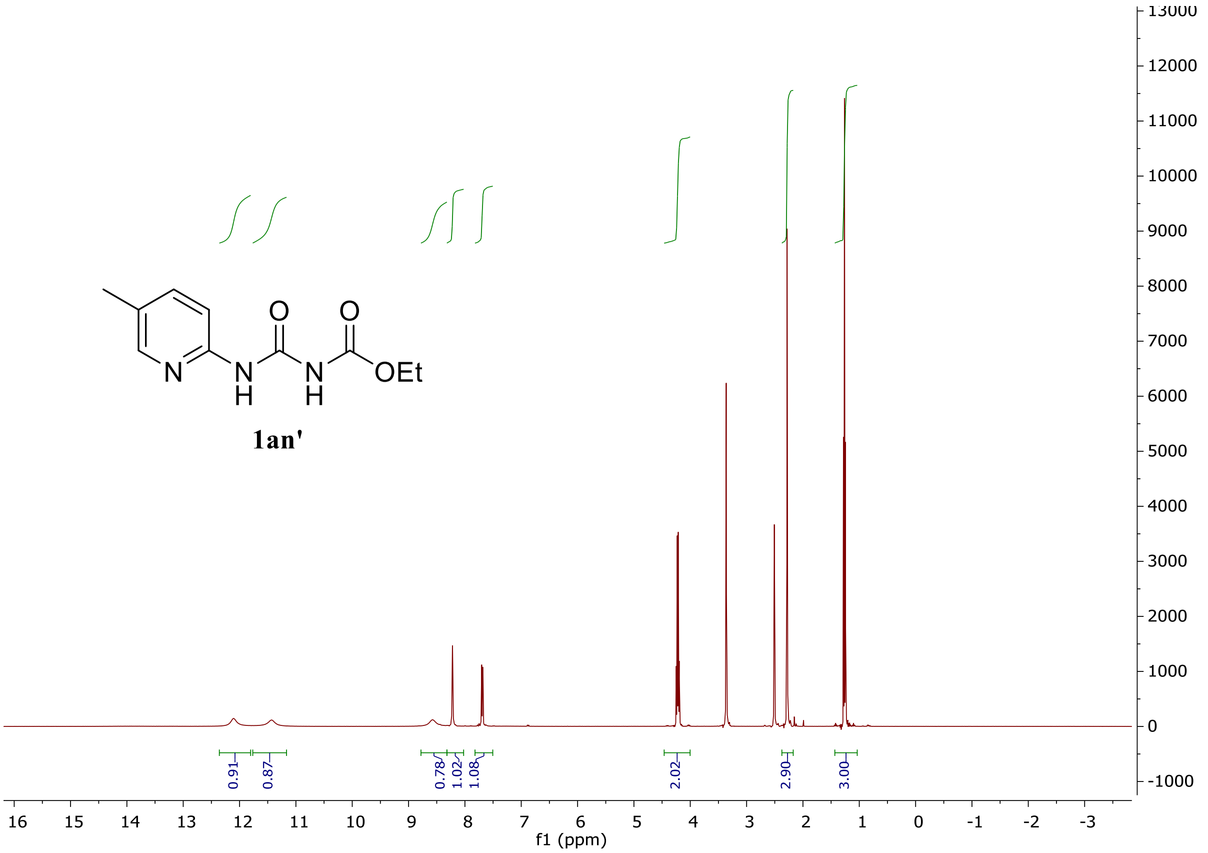
N
N
H
O
N
H
O
OEt
0.91
0.87
0.78
1.02
1.08
2.02
2.90
3.00
f1 (ppm)

1an'

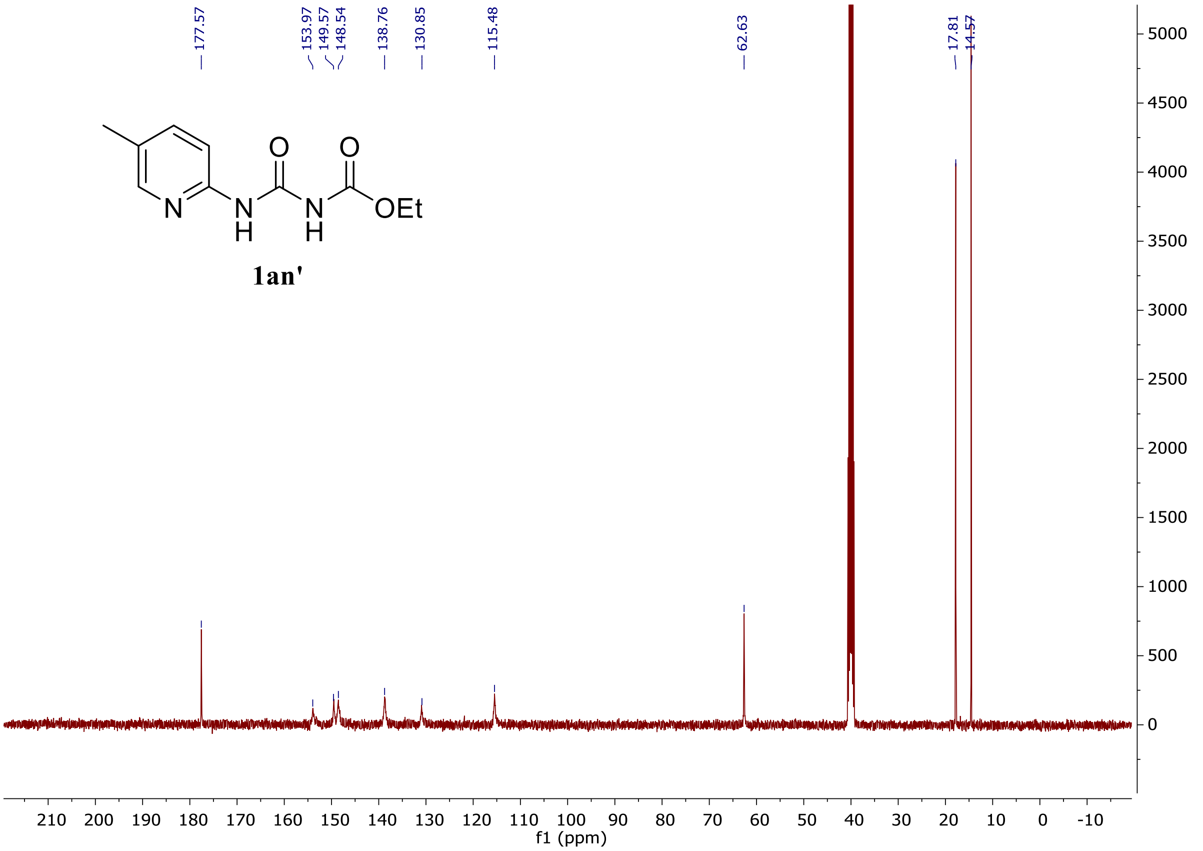
177.57
153.97
149.57
148.54
138.76
130.85
115.48
62.63
17.81
14.57
O
O
N
N
H
N
H
OEt
1an'
f1 (ppm)

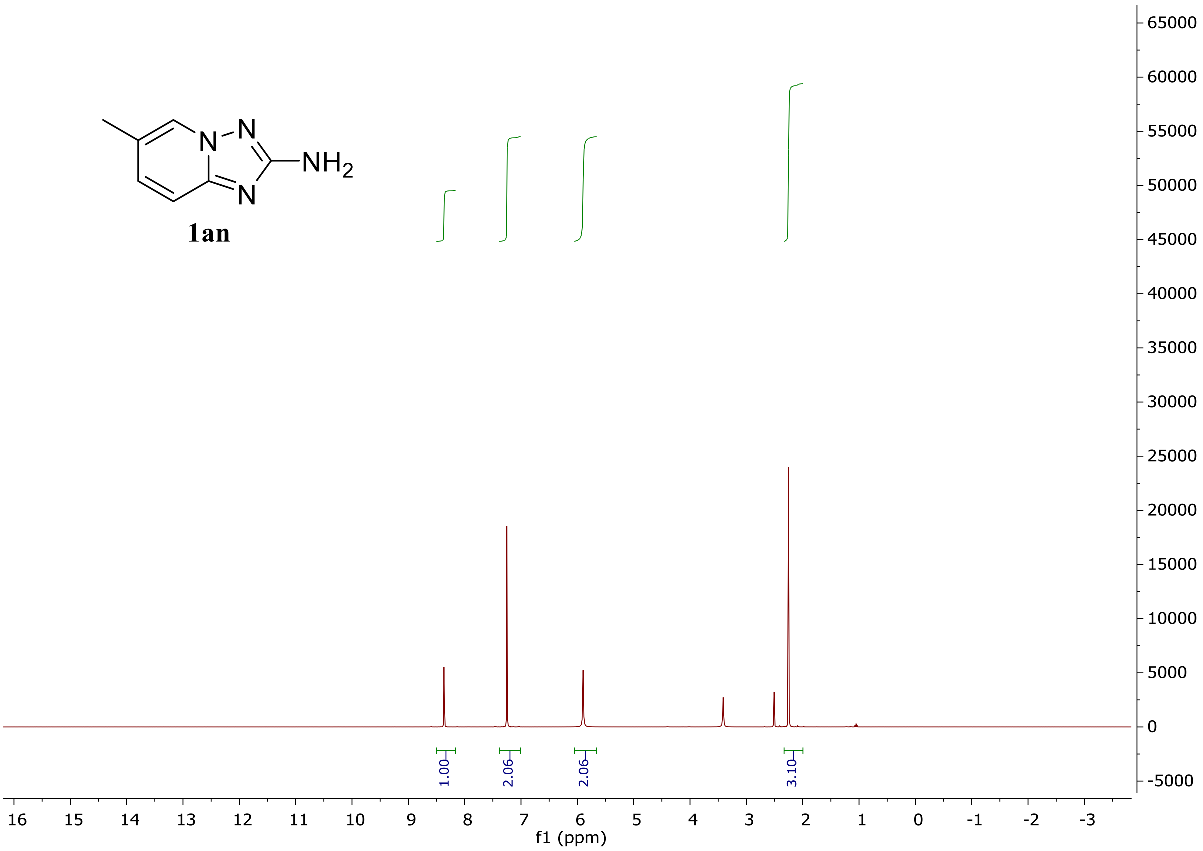
N
N
N
NH2
1an
1.00
2.06
2.06
3.10
f1 (ppm)

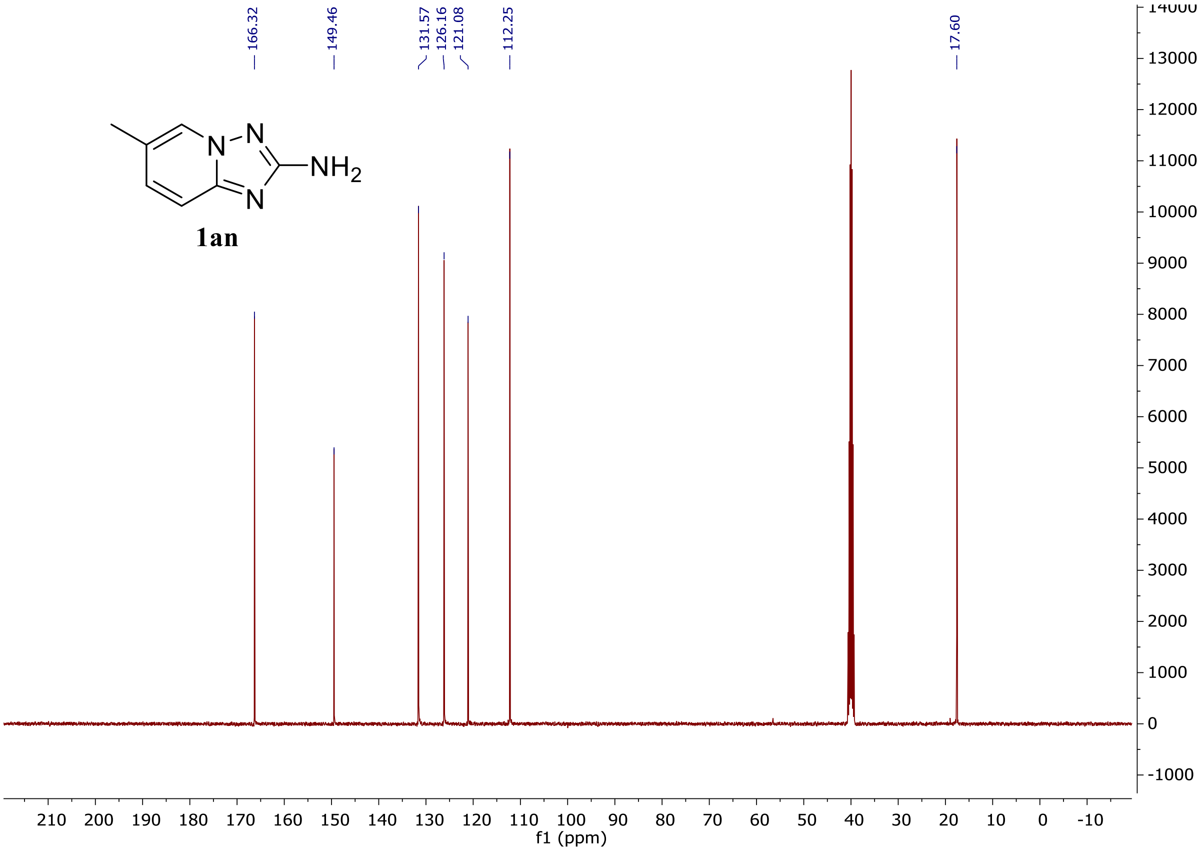
166.32
149.46
131.57
126.16
121.08
112.25
17.60
NH2
N
N
N
1an
14000
13000
12000
11000
10000
9000
8000
7000
6000
5000
4000
3000
2000
1000
0
-1000
210 200 190 180 170 160 150 140 130 120 110 100 90 80 70 60 50 40 30 20 10 0 -10
f1 (ppm)

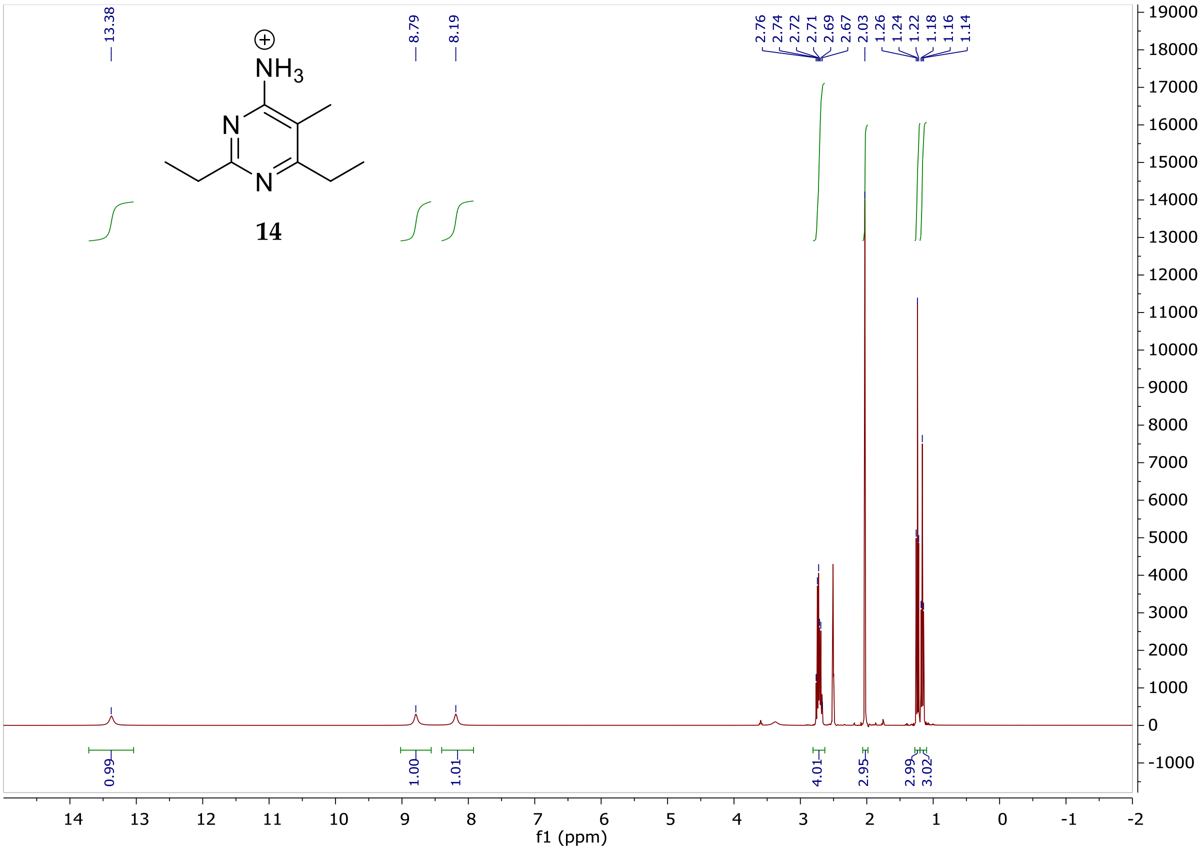
13.38
8.79
8.19
2.76
2.74
2.72
2.71
2.69
2.67
2.03
1.26
1.24
1.22
1.18
1.16
1.14
NH3
N
N
14
0.99
1.00
1.01
4.01
2.95
2.99
3.02
f1 (ppm)

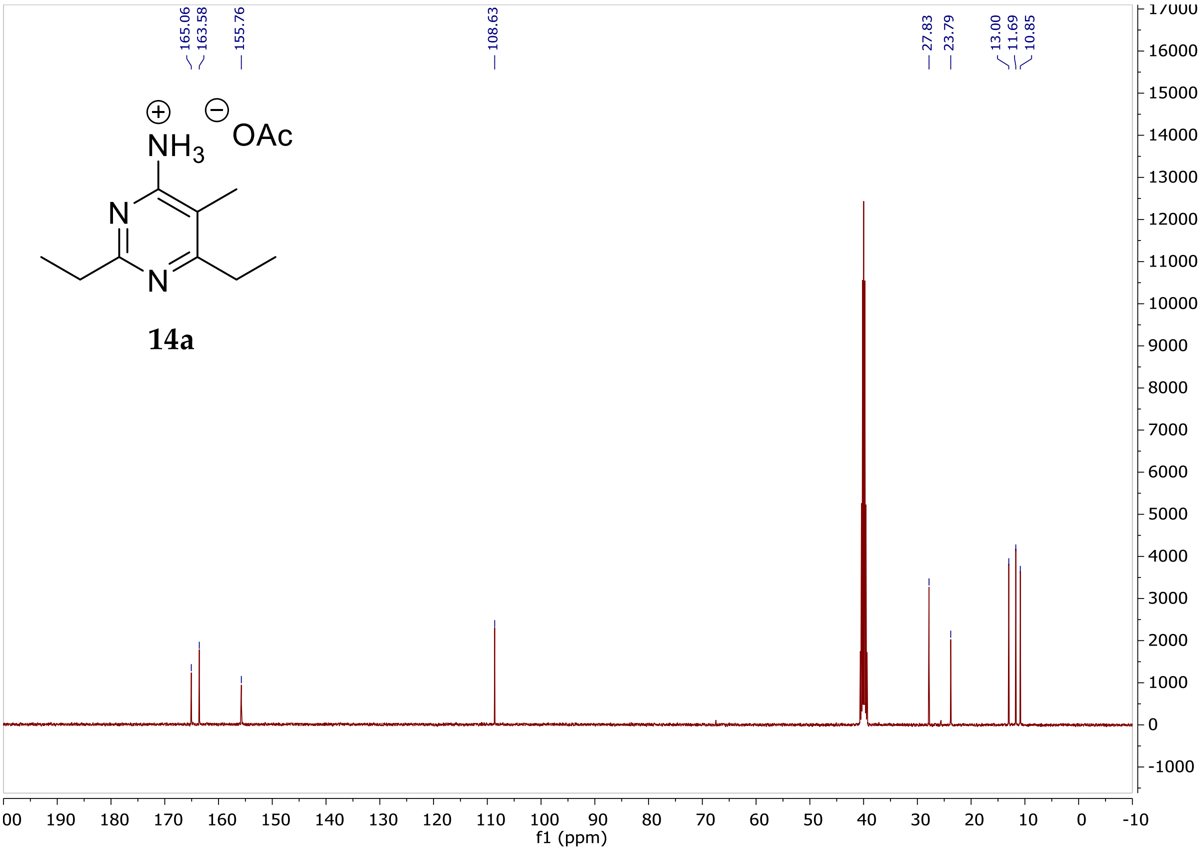
165.06
163.58
155.76
108.63
27.83
23.79
13.00
11.69
10.85
NH3
OAc
N
N
14a
f1 (ppm)

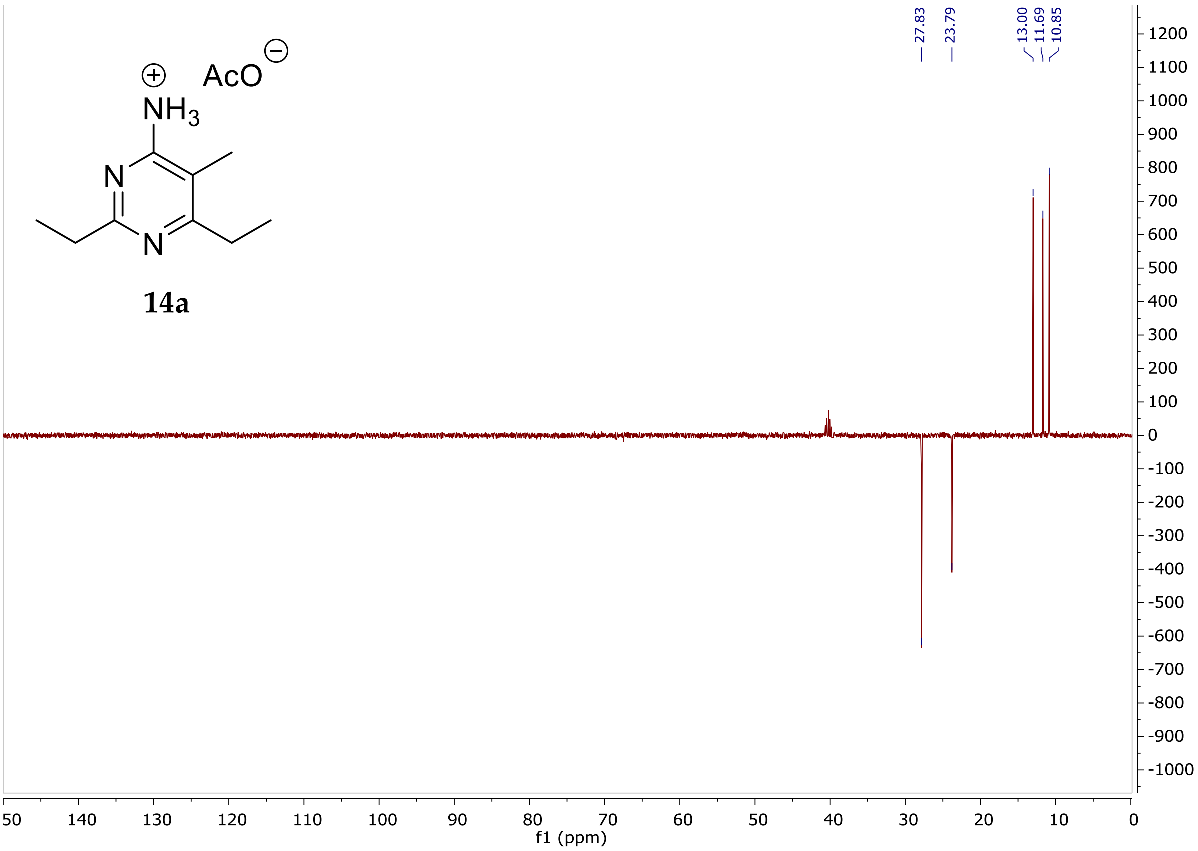

AcO
NH3
N
N
14a
27.83
23.79
13.00
11.69
10.85
f1 (ppm)
